# Functional contribution of the intestinal microbiome in autism spectrum disorder, attention deficit hyperactivity disorder, and Rett syndrome: a systematic review of pediatric and adult studies

**DOI:** 10.3389/fnins.2024.1341656

**Published:** 2024-03-07

**Authors:** Valentina Caputi, Lee Hill, Melanie Figueiredo, Jelena Popov, Emily Hartung, Kara Gross Margolis, Kanish Baskaran, Papiha Joharapurkar, Michal Moshkovich, Nikhil Pai

**Affiliations:** ^1^Poultry Production and Product Safety Research Unit, Agricultural Research Service, United States Department of Agriculture, Fayetteville, AR, United States; ^2^Department of Pediatrics, Faculty of Health Sciences, McMaster University, Hamilton, ON, Canada; ^3^Department of Pediatrics, Research Institute of the McGill University Health Centre, Montreal, QC, Canada; ^4^Temerty Faculty of Medicine, University of Toronto, Toronto, ON, Canada; ^5^Harvard Medical School, Boston, MA, United States; ^6^Boston Children’s Hospital, Boston, MA, United States; ^7^Department of Biochemistry and Biomedical Sciences, Faculty of Science, McMaster University, Hamilton, ON, Canada; ^8^Department of Pediatrics, New York University Grossman School of Medicine, New York, NY, United States; ^9^New York University Pain Research Center, New York, NY, United States; ^10^New York University College of Dentistry, New York, NY, United States; ^11^Division of Gastroenterology, Hepatology and Nutrition, McMaster Children’s Hospital, Hamilton, ON, Canada; ^12^Department of Pediatrics, Perelman School of Medicine at the University of Pennsylvania, Philadelphia, PA, United States; ^13^Division of Gastroenterology, Hepatology, and Nutrition, the Children’s Hospital of Philadelphia, Philadelphia, PA, United States

**Keywords:** autism spectrum disorder, attention deficit hyperactivity disorder, Rett syndrome, pediatric, children, microbiota, metabolites, neurodevelopment

## Abstract

**Introduction:**

Critical phases of neurodevelopment and gut microbiota diversification occur in early life and both processes are impacted by genetic and environmental factors. Recent studies have shown the presence of gut microbiota alterations in neurodevelopmental disorders. Here we performed a systematic review of alterations of the intestinal microbiota composition and function in pediatric and adult patients affected by autism spectrum disorder (ASD), attention-deficit/hyperactivity disorder (ADHD), and Rett syndrome (RETT).

**Methods:**

We searched selected keywords in the online databases of PubMed, Cochrane, and OVID (January 1980 to December 2021) with secondary review of references of eligible articles. Two reviewers independently performed critical appraisals on the included articles using the Critical Appraisal Skills Program for each study design.

**Results:**

Our systematic review identified 18, 7, and 3 original articles describing intestinal microbiota profiles in ASD, ADHD, and RETT, respectively. Decreased Firmicutes and increased Bacteroidetes were observed in the gut microbiota of individuals affected by ASD and ADHD. Proinflammatory cytokines, short-chain fatty acids and neurotransmitter levels were altered in ASD and RETT. Constipation and visceral pain were related to changes in the gut microbiota in patients affected by ASD and RETT. Hyperactivity and impulsivity were negatively correlated with *Faecalibacterium* (phylum Firmicutes) and positively correlated with *Bacteroides* sp. (phylum Bacteroidetes) in ADHD subjects. Five studies explored microbiota-or diet-targeted interventions in ASD and ADHD. Probiotic treatments with *Lactobacillus* sp. and fecal microbiota transplantation from healthy donors reduced constipation and ameliorated ASD symptoms in affected children. Perinatal administration of *Lactobacillus* sp. prevented the onset of Asperger and ADHD symptoms in adolescence. Micronutrient supplementation improved disease symptomatology in ADHD without causing significant changes in microbiota communities’ composition.

**Discussion:**

Several discrepancies were found among the included studies, primarily due to sample size, variations in dietary practices, and a high prevalence of functional gastrointestinal symptoms. Further studies employing longitudinal study designs, larger sample sizes and multi-omics technologies are warranted to identify the functional contribution of the intestinal microbiota in developmental trajectories of the human brain and neurobehavior.

**Systematic review registration:**

https://clinicaltrials.gov/, CRD42020158734.

## Introduction

Over the last decade, the field of neuroscience has benefited from the rapid evolution of microbiology and microbiome science. This includes new insights into the pathophysiology of neurodevelopmental disorders. The Developmental Origins of Health and Disease (DOHaD) hypothesis proposes that early life influences can dramatically impact the health of infants. A combination of genetic predisposition and environmental triggers may have deleterious effects on infants’ neurodevelopmental programming, predisposing individuals to long-lasting behavioral and cognitive alterations. This has been described in children with conditions such as autism spectrum disorders (ASD) (Santos et al., 2022), attention deficit hyperactivity disorder (ADHD) (Hare et al., 2022; Makris et al., 2022) and Rett syndrome (RETT) (Hoffbuhr et al., 2002).

ASD comprises a spectrum of neurodevelopmental disorders characterized by deficits in social interactions and communication skills. Affected individuals are often male and may present with unusual repetitive behaviors, restricted interests, difficulties with communication and marked impairments in daily functioning. Personal, social, school, and work life are often affected (Harris, 2018). The spectrum of ASD includes autism (AD), Asperger’s Syndrome (AS) and, prior to the 2013 edition of Diagnostic and Statistical Manual of Mental Disorders (DSM) (American Psychiatric Association, 2013), Pervasive Developmental Disorder Not Otherwise Specified (PDD-NOS) (Haglund and Källén, 2011; Tateno et al., 2011).

ADHD is a frequently diagnosed condition in children and adults. ADHD affects approximately 7.2% of children worldwide and up to 3-10% of school-aged children (Lange et al., 2010; Polanczyk et al., 2014; Thomas et al., 2015). The underlying pathophysiology of this neurodevelopmental disorder involves disturbances in neural responses to dopamine and noradrenaline stimulation and deficits in reward processing via the ventral striatum. Genome-wide association studies have identified pathognomonic abnormalities in serotonin, dopamine, and noradrenaline-related genes (Guimarães et al., 2007; Ghosh et al., 2013; Abraham et al., 2020).

RETT is a rare genetic disorder, principally affecting females, caused by loss-of-function mutations of the X-linked methyl-CpG binding protein 2 (MeCP2) gene (Borghi and Vignoli, 2019). Individuals with RETT are characterized by loss of purposeful hand use and spoken communication skills, as well as the development of stereotypic hand movements and gait abnormalities after a period of apparently normal development. RETT shares several common features with ASD, yet clear differences exist between the two diseases (Percy, 2011).

Patients with ASD, ADHD and RETT have a high prevalence of functional gastrointestinal disorders (FGID) (Adams et al., 2011; Motil et al., 2012; Severance et al., 2015; Bermudez et al., 2019; Kedem et al., 2020), now referred as disorders of gut brain interaction (DGBI) (Camilleri, 2023; for the purposes of consistency with literature assessed in our systematic review, we will continue to use FGDI) and stereotypic dietary habits (Wong et al., 2021; Bourne et al., 2022; Salvat et al., 2022). These observations have led to increased research toward identifying differences in the gastrointestinal (GI) system across these neurodevelopmental disorders with a particular focus on the microbiota. Gut microbes and their collective genome (the microbiome) may be considered an essential organ for maintaining the homeostasis of intestinal and extraintestinal body sites throughout the lifespan (Cryan et al., 2020). This complex microbial community has been recognized to influence brain neurophysiology and behavior by communicating with the central nervous system (CNS) through multiple neuronal, immune, and endocrine signaling pathways involved in the microbiota-gut-brain axis (MGBA) (Luczynski et al., 2016; Cryan et al., 2019). In response, the brain establishes appropriate physiological responses to direct mental and physical functions (Fung et al., 2017; Cryan et al., 2019; Bioque et al., 2021).

Human perinatal and early neurodevelopment are characterized by sensitive periods or “critical windows” in which the biological systems involved in brain development (i.e., neurogenesis, neuronal differentiation and migration, synaptic rearrangement) show significant plasticity. These periods are uniquely susceptible and responsive to environmental stimuli (i.e., hypoxia, trauma, infection or inflammation; Cowan et al., 2020; Lopatina et al., 2021). The gut microbiota changes and evolves rapidly during these same temporal windows, and this parallels numerous physiologic trajectories (immunity, metabolism, stress responsivity, and neuroglial formation) within the MGBA (Borre et al., 2014; Cowan et al., 2020; Bioque et al., 2021). Thus, these changes may have long-lasting consequences for brain function, behavior and psychiatric well-being (Cryan et al., 2019; Margolis et al., 2021).

The central nervous system’s maturation begins in the first trimester of gestation with the formation of the neural tube, which becomes organized into the brain and spinal cord. Concurrent development of the blood brain barrier (BBB) begins at gestational week eight and continues throughout pregnancy. In the third trimester of pregnancy, the human brain doubles in size and oligodendrocytes proliferate to start the process of myelination (Borre et al., 2014). Critical events for normal brain development, such as hippocampal neurogenesis and gliogenesis, occur during these early gestation phases and continue through the postnatal period up to 3 years of age (Borre et al., 2014; Lopatina et al., 2021). The BBB, while functionally active by week 14 of gestation, continues to undergo maturation into an individuals’ third decade (Saili et al., 2017).

The assembly of gut microbial communities in early life then occurs as a dynamic process that is significantly influenced by diet as babies shift from exclusive breastmilk or formula intake to complementary solid foods (Ratsika et al., 2021). By the age of 3 years, the microbiota composition reaches a more stable, adult-like phenotype (Tanaka and Nakayama, 2017).

Emerging evidence shows that host-microbiota crosstalk through the immune system is fundamental to preserving the integrity of the intestinal and peripheral epithelial-immune-endothelial barriers (such as the BBB). Within the central nervous system, bacterial microbes help regulate the development of the microglia and other resident immune cells which help sustain and protect neuronal function (Fung et al., 2017). Intestinal microbes can produce a variety of neurotransmitters, as well as microbial-derived neuroactive compounds such as short-chain fatty acids (SCFA) that modulate the peripheral and central nervous system, and immune system (Fung et al., 2017; Erny et al., 2021; Popov et al., 2021).

Disturbances of the microbiota-immune axis in early life affect intestinal barrier permeability, leading to systemic translocation of bacterial-derived products that may cross the BBB. The infant BBB is still immature until at least 4 months of age, leading to microglia hyperactivation within the CNS and neuroinflammation (Hill et al., 2021). In the setting of intestinal inflammation and increased intestinal permeability, the translocation of bacterial products into the systemic circulation further increases. Ordinarily, endothelial structures outside the BBB (e.g., choroid plexus) block the passage of substrates from the periphery to the brain, thus protecting the CNS from the progressive increase of pro-inflammatory factors (Carloni et al., 1979). This response compromises the bidirectional flow of key signaling molecules and hormones between the gut-brain components of the MGBA (Carloni et al., 1979). Pathogens that have molecular mimicry with host antigens can exert effects by causing immunological cross-reactions and autoimmunity. These have been associated with both neuroinflammation and behavioral affective disorders (Hill et al., 2021).

Causal relationships between changes in the microbiome and behavioral outcomes remain unclear (Laue et al., 2021; Yap et al., 2021). Studies of the microbiome in pediatric neurodevelopmental disorders face persistent questions of temporal dynamics of behavioral symptoms, and the independent impacts of key variables known to alter microbiota composition, including dietary intake, GI comorbidities, and/or sex differences (Baikie et al., 2014; Laue et al., 2021; Yap et al., 2021; Tarui et al., 2022). Nevertheless, recent advances in sequencing technologies and bioinformatics are facilitating the concomitant examination of subtle microbiome changes with behavioral outcomes in animal models or human subjects affected by pediatric neurodevelopmental disorders (Berg et al., 2020; Morais et al., 2021; Socała et al., 2021).

In this systematic review, we provide a comprehensive overview of research on the diverse presence and potential roles of the gut microbiome in ASD, ADHD and RETT. Our protocol identified case–control and cohort studies investigating the intestinal microbiome in pediatric patients affected by ASD, and pediatric and adult patients’ affected by ADHD (Hill et al., 2021) and Rett syndrome. We also summarize other critical factors that play essential roles in brain-gut crosstalk including molecular metabolites, immunologic markers, and neurodevelopmental and GI symptoms. We discuss potential changes in host-microbe immune and endocrine interactions, alterations in microbial-derived metabolite production and inflammatory and other biochemical pathways associated with these relevant disorders. Finally, we explore microbiota-targeted interventions that have been shown to improve symptoms.

## Methods

### Study design

We have previously published our protocol for this systematic review, consistent with the Preferred Reporting Items for Systematic review and Meta-analysis (PRISMA) protocols checklist (Liberati et al., 2009; Shamseer et al., 2015). The systematic review protocol (Hill et al., 2021) is registered on PROSPERO (ID: CRD42020158734).

### Data sources and search strategy

We appraised available evidence surrounding the gut microbiota in ASD, ADHD, and RETT (Hill et al., 2021). Relevant studies were identified using PubMed, Cochrane Library, and OVID electronic databases. Articles published between January 1980 and December 2021 were considered for inclusion and analysis.

We implemented a rigorous search strategy that involved two rounds of literature review, using a three-step method in each round (Hill et al., 2021). The first round involved a review of articles from the database search results; the second round involved a review of works cited by those articles identified in the first round. Titles, abstracts, and full texts were independently screened by at least two authors (LH, JP, MF, VC, EH, MM, or NP). Articles were excluded if they were unrelated or met exclusion criteria (Supplementary Table S1). Inclusion/exclusion criteria were slightly modified for ADHD and RETT studies. The age of the patients was >18 for three of the seven included ADHD studies. In all included studies on RETT, the age range was between 5 and 36 years. We decided to include these studies as they met other inclusion criteria, and they were the only available studies that included human participants (Supplementary Table S1). Two reviewers (MF and LH) completed data extraction using a pre-piloted Microsoft Excel data extraction form. Data extraction was subsequently checked for accuracy and completeness by members of the study team (NP, JP, EH, MM, and VC). One further study was included upon consultation with external content experts (KM and EA) (Luna et al., 2017).

### Evidence quality assessment

We assessed quality of the included studies using the appropriate Critical Appraisal Skills Program (CASP) checklist based on study methodology. CASP was selected for its quality of appraisal of health-related studies, assessment of transparency of research practice and reporting standards (Long et al., 2020). Level of evidence was assessed using established methods and a “level of evidence score” was established (Obremskey et al., 2005). Two independent reviewers assessed each study and, if discrepancies occurred, a third independent reviewer was included. We applied a semi-quantitative appraisal of CASP checklists based on an approach by Nichols et al. (2020) where study quality was scored out of 20.

## Results

### Study selection and quality assessment

**Figure 1:**
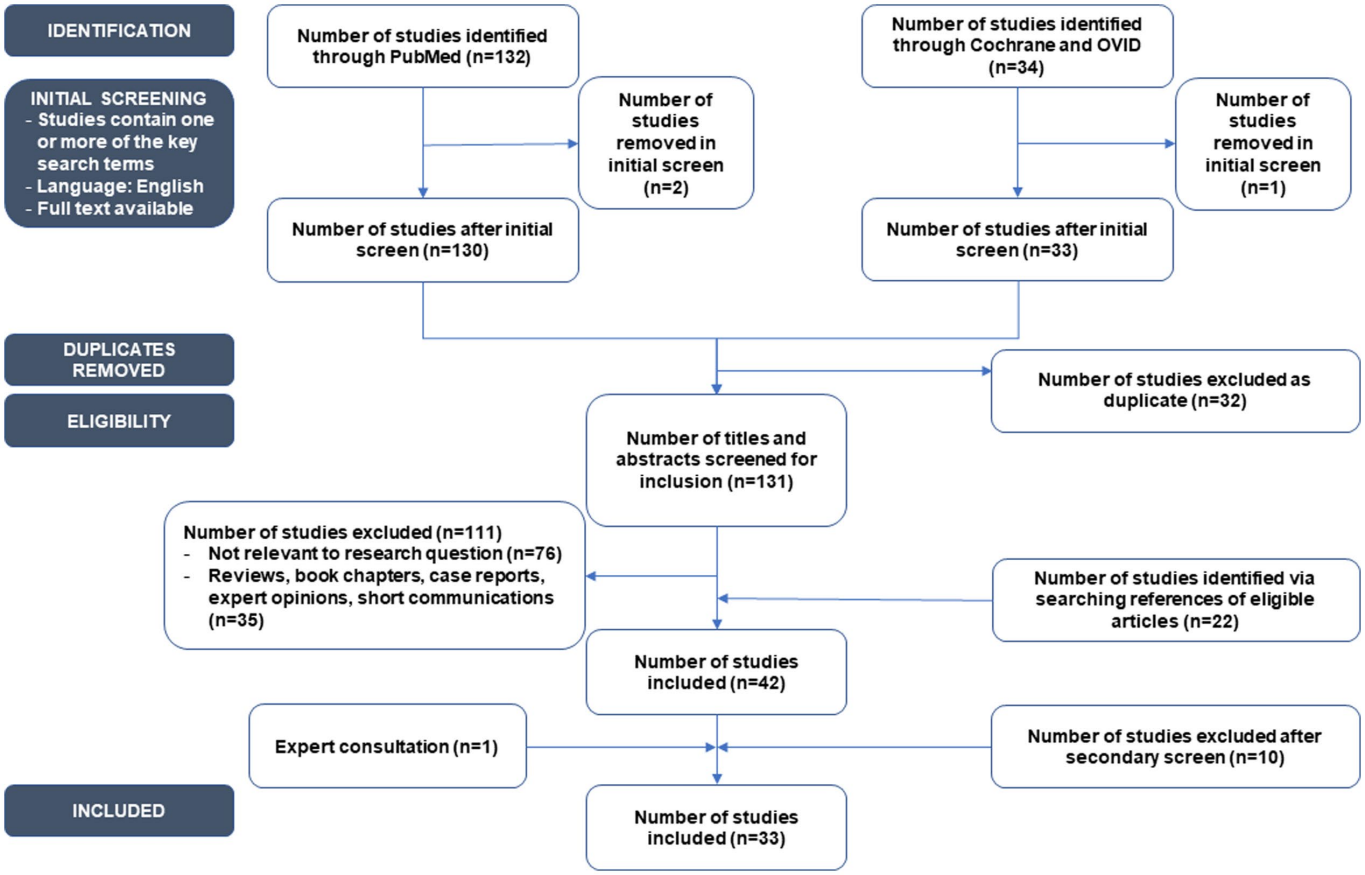
PRISMA-ScR systematic flowchart of the systematic review process.

The initial search strategy outlined in our protocol (Hill et al., 2021) identified 166 articles. After initial screening and removal of duplicates, 131 articles were screened for inclusion. Finally, after review with external content experts 33 original studies that described the association between intestinal microbiota and ASD, ADHD or RETT in children and adults were included (Figure 1). Twenty-six studies were assessed using the CASP Checklist for Case–Control Studies (Supplementary Table S2), five using the CASP Checklist for Cohort Studies (Supplementary Table S3), and two for RCT studies (Supplementary Table S4).

Scores for each study ranged from 7 (low methodological quality) to 20 (high methodological quality). Two studies (6.9%) had a total score below 10, suggesting weak methodology (Zhang et al., 2018; Ahmed et al., 2020). The majority of studies (75.9%) had a total score between 15 and 20, and eight studies achieved the highest score for methodological quality (De Angelis et al., 2013; Pärtty et al., 2015; Tomova et al., 2015; Strati et al., 2016, 2017; Kang et al., 2018; Zhai et al., 2019; Wan et al., 2020).

### Characteristics of studies investigating the intestinal microbiota in ASD, ADHD, and Rett syndrome

Eighteen case–control studies assessed the intestinal microbiota in ASD, seven in ADHD (four cohort studies, three case–control), and three in RETT (one cohort, two case–control; Tables 1–4; Supplementary Tables S5–S8). Geographically, the studies included patients from different countries including the United States, China, India, Australia, Egypt, Germany, Italy, and The Netherlands. The studies included in our review encompassed a total of 2,251 subjects with ASD (among which 10 subjects were diagnosed with PDD-NOS; Supplementary Tables S5, S6), 272 subjects with ADHD (Supplementary Table S7) and 101 subjects with RETT (Supplementary Table S8). Control groups included 1,059 sex- and age- matched neurotypical (NT) subjects in studies of ASD and PDD-NOS, 321 NT subjects in studies of ADHD, and 60 NT subjects in studies of RETT (Supplementary Tables S5–S8). Ages of cases and controls spanned 2 to 18 years old for studies of ASD (Tables 1, 2; Supplementary Tables S5, S6), 6 to 44 years for studies of ADHD (Table 3; Supplementary Table S7), and 5 to 36 years for studies of RETT (Table 4; Supplementary Table S8). Subjects with ASD, PDD-NOS, ADHD and respective NT controls were males and females, while subjects with RETT and their NT controls were all females.

**Table 1:** Evidence of microbiota alterations in ASD compared to unrelated NT controls.

Study characteristics	Microbiota assessment	Increasing microbial taxa	Decreasing microbial taxa	Metabolites/Immune markers	Functional pathways
Zhang et al. (2018) Case–control (CASP Score: 7) Sample size: *N* = 47 Age range (years): 3-8 Dietary habits: Not investigated GI symptoms: Not investigated	16S rRNA sequencing (Stool samples) Alpha diversity No differences (Shannon index) Beta diversity ASD microbiota is different from NT ↑Bacteroidetes/Firmicutes ratio	Phylum ↑Bacteroidetes Genus No increase was observed	Phylum ↓Firmicutes Genus ↓*Streptococcus*, *Veillonella* and *Escherichia*	Microbial-derived metabolites: Not investigated ↑abundance of butyrate-and lactate-producers’ bacteria in NT (*Not statistically significant*) ↑abundance of mucin-degraders- and other SCFAs-producers- bacteria in ASD (*Not statistically significant*)	↑D − Arginine and D − ornithine metabolism, ether lipid metabolism, bacterial chemotaxis, phosphotransferase system and flagellar assembly genes in ASD ↑meiosis-yeast, steroid hormone biosynthesis, glycosaminoglycan degradation and lipoic acid metabolism in NT (*Not statistically significant*) (PICRUSt-KEGG database)
Liu et al. (2019) Case–control (CASP Score: 15) Sample size: *N* = 50 Age range (years): 2.5-18 Dietary habits: Not investigated GI symptoms: ↑constipation in ASD	16S rRNA sequencing (Stool samples) Alpha diversity ↓species diversity and evenness (Shannon index) in ASD Beta diversity ASD microbiota is different from NT; ASD-C microbiota is different from NT and ASD	Phylum ↑Acidobacteria Family ↑*Veillonellaceae* *Enterobacteriaceae* *Pseudomonadaceae* *Enterococcaceae* Genus ↑*Megamonas*	Phylum ↓Firmicutes Family ↓Oscillospiraceae* Streptococcaceae *Peptostreptococcaceae* *Erysipelotrichaceae* Genus ↓*Eubacterium and Lachnospiraceae-NC2004*	Microbial-derived metabolites: ↓acetic acid and butyrate in ASD ↑valeric acid in ASD Positive correlations: Acidobacteria with valeric acid *Desulfovibrionaceae* and *Streptococcaceae* with propionic acid. *Desulfovibrionaceae* with acetic acid Butyrate producing bacteria with butyrate	Not investigated
Kang et al. (2013) Case–control (CASP Score: 17) Sample size: *N* = 40 Age range (years): 3-16 Dietary habits: Documented and included in the analysis GI symptoms: ↑GI symptoms in ASD	16S rRNA sequencing (Stool samples) Alpha diversity ↓number of observed species (Chao1 index) in ASD ↓phylogenetic diversity in ASD (no influence of the diet) Beta diversity Not investigated Negative correlation between bacterial richness and GI symptoms ↓bacterial richness in ASD with severe GI symptoms	Family No increase was observed Genus ↑*Akkermansia*	Family ↓*Veillonellaceae* *Prevotellaceae* Genus ↓*Prevotella* *Coprococcus*	Microbial-derived metabolites: Not investigated Possible outcomes: ↓*Veillonellaceae* in ASD- > less ability to ferment lactate ↓*Coprococcus* in ASD - > less butyrate production	Not investigated
Kang et al. (2018) Case–control (CASP Score: 14) Sample size: *N* = 44 Age range (years): 4-17 Dietary habits: Documented; not included in the analysis GI symptoms: ↑severe GI symptoms in ASD	16S rRNA sequencing (Stool samples) Alpha diversity ↓bacterial diversity (observed OTUs) in ASD Beta diversity ASD microbiota is different from NT	Genus No increase was observed	Genus ↓*Prevotella*, *Coprococcus* *Fecalibacterium* *Haemophilus* *Streptococcus*	Microbial-derived metabolites: no changes in propionate and butyrate ↑isopropanol in ASD stool (potential increase in isopropanol-producing bacteria) ↑p-cresol (toxic for colonic epithelial cells) in ASD stool ↑caprate and aspartate in ASD stool (potential alteration in NMDA receptor’s function) ↓GABA levels in ASD stool-no changes in glutamate ↓nicotinate, glutamine, and thymine in ASD stool (potential changes in CNS neurotransmitter production)	-no differences in bacterial pathways between ASD and NT -no differences in genes of the metabolic pathways associated with p-cresol, vitamin K, and GABA -Isopropanol producing and degrading enzymes were not investigated, since they are not listed in the database (PICRUSt-KEGG database)
Rose et al. (2018) Case–control (CASP Score: 16) Sample size: *N* = 91 (stool) *N* = 87 (blood) Age range (years): 3-12 Dietary habits: Documented; not included in the analysis GI symptoms: NT and ASD with/without GI symptoms (4 groups)	16S rRNA sequencing (Stool samples) Alpha diversity Not investigated Beta diversity 3 clusters: (i) Bacteroides enriched (ii) ASD vs. NT (iii) *Prevotella* enriched (NT and ASD without GI symptoms)	Family ↑*Bacteriodaceae*, *Lachnospiraceae*, *Prevotellaceae* in ASD-GI (compared to NT-GI) No difference at family level between ASD-No GI and NT-No GI	Family ↓Oscillospiraceae* in ASD-GI (compared to NT-GI) No difference at family level between ASD-No GI and NT-No GI	Immune-inflammatory markers: *TLR4 stimulation in PBMC*: ↑IL-1α, TNFα, IL-1β in ASD-NoGI ↑cytokines production in ASD-GI *NOD stimulation in PBMC:* ↑cytokines production in ASD-GI ↓ cytokines production in ASD-No-GI *PHA stimulation in PBMC* ↑IL-15 in ASD-No-GI ↑IFNɣ in ASD-GI ↓TGFβ1 in ASD-GI	↑the amino sugars and nucleotide sugars pathways in ASD-GI compared to ASD-NoGI ↓starch and sucrose metabolism pathways in ASD-NoGI, but slightly upregulated in ASD-GI (PICRUSt-KEGG database)
Finegold et al. (2017) Case–control (CASP Score: 14) Sample size: *N* = 46 Age range (years): 2-9 Dietary habits: Not investigated GI symptoms: ↑beta2-toxin from *C. perfringens* in ASD with GI symptoms	No sequencing. PCR or cultured bacteria (Stool sample) Alpha diversity Not investigated Beta diversity Not investigated	Species ↑*C. perfringens* in ASD	Species No decrease was observed	Not investigated Possible outcomes: Immune system potentially compromised by the ↑ *C. perfringens* and beta-2-toxin	Not investigated
Zhai et al. (2019) Case–control (CASP Score: 20) Sample size: *N* = 136 Age range (years): 3-6 Dietary habits: Not investigated GI symptoms: 80% ASD with GI symptoms	16S rRNA sequencing (Stool samples) Alpha diversity ↑richness in ASD compared to NT Beta diversity ASD microbiota is different from NT ↑Bacteroidetes/Firmicutes ratio	Phylum ↑Bacteroides Genus ↑*Bacteroides* *Parabacteroides* *Sutterella* *Lachnospira* *Bacillus* *Bilophila* *Lactococcus* *Lachnobacterium* *Oscillospira*	Phylum No decrease was observed Genus No decrease was observed	Trace elements: ↑levels of Pb, As, Cu, Zn, Mg, Ca and Hg in ASD female and male (in males Hg levels were comparable to NT) Possible outcomes: These elements are altered in neurodegenerative disorders (i.e., PD) -positive correlation between *Oscillospira* and *Parabacteroides* with As, Hg (risk factors in ADHD and cerebral palsy-neurotoxic element) Possible outcomes: These are neurotoxic elements and risk factors for ADHD and cerebral palsy	Positive correlation of *Bacteroides, Oscillospira* and *Sutterella* with citrate cycle, carbon fixation (prokaryotes), gas degradation (all *downregulated pathways* in ASD) -negative correlation of *Parabacteroides* with ether lipid metabolism and sporulation -negative correlation of *Bacteroides*, *Oscillospira and Sutterella* with porphyrin and chlorophyll metabolism -negative correlation of *Bacteroides*, and *Sutterella* with carotenoid biosynthesis metabolism (All *upregulated pathways* in ASD) (PICRUSt-KEGG database)
Strati et al. (2017) Case–control (CASP Score: 20) Sample size: *N* = 80 Age range (years): 2-16 Dietary habits: Not investigated GI symptoms: ASD with/without constipation	16S rRNA sequencing Fungal ITS1 rDNA region (Stool samples) Bacterial diversity: Alpha diversity No differences Beta diversity ASD microbiota is different from NT ↑Firmicutes/Bacteroidetes ratio (already observed in IBDs, obesity) Fungal diversity: Alpha diversity No differences Beta diversity ASD mycobiome is different from NT	Genus (Bacteria) ↑*Collinsella* *Corynebacterium* *Dorea* *Lactobacillus* Genus (Fungi) *↑Candida*	Genus (Bacteria) ↓*Prevotella* *Alistipes* *Bilophila* *Dialister* *Parabacteroides* *Veillonella* Genus (Fungi) No decrease was observed	Inflammatory markers: No difference in ESR, fecal calprotectin levels and serum IgA among constipated and non-constipated ASD and among ASD and NT ↑*Candida* in ASD Possible outcomes: potential changes in the abundance of *Bacteriodetes*, *Lactobacillaceae*, *Ruminococcaceae, Lachnospiraceae* (these bacteria produce IL-22 and IL-17, anti-*Candida* cytokines)	Not investigated
Hughes and Ashwood (2018) Case–control (CASP Score: 18) Sample size: *N* = 78 Age range (years): 3-13 Dietary habits: Not investigated GI symptoms: 40% ASD with GI symptoms	No microbiota sequencing. anti-Candida IgG levels (in plasma samples)	Not investigated	Not investigated	Immune/inflammatory markers: ↑anti-*C. albicans* IgG in the blood of ASD Possible outcomes: Potential ↑relative abundance of *C. albicans* in ASD Potential increase of IL-17 (yet implicated in the MIA model of ASD)	Not investigated
Kantarcioglu et al. (2016) Case–control (CASP Score: 14) Sample size: *N* = 1958 Age range (years): 1-18 Dietary habits: Not investigated GI symptoms: Not investigated	No microbiota sequencing Microbial cultures (*Candida* species isolated only from ASD and not NT)	Species (Fungi) ↑*C. albicans* *C. krusei* *C. glabrata*	Species No decrease was observed	Not investigated	Not investigated
Kushak et al. (2017) Case–control (CASP Score: 15) Sample size: *N* = 40 Age range (years): 14-16 Dietary habits: Documented; not included in the analysis GI symptoms: Present in both ASD and NT	16S rRNA sequencing (Duodenal mucosa samples) Alpha diversity No differences Beta diversity No differences	Genus ↑*Burkholderia* *Oscillospira*, *Actinomyces*, *Peptostreptococcus*, *Ralstonia*	Genus ↓*Neisseria* *Devosia*, *Prevotella*, *Bacteroides*, *Streptococcus*	Mucosal enzymes: In ASD subjects: -positive correlation between *Bacteroides, Faecalibacterium, Clostridium* and disaccharidase and lactase activities -positive correlation of *Clostridium* with maltase, palatinase, and sucrase activities -positive correlation of *Bacteroides* sp. with lactase activity -positive correlation of *Clostridium* sp. with lactase, maltase, palatinase, and sucrase activities In NT subjects: -positive correlation of *Porphyromonas, Barnesiella, Gemella*, and *Leptotrichia* with lactase activity	Not investigated
Luna et al. (2017) Case–control (CASP Score: 15) Sample size: *N* = 80 Age range (years): 3-18 Dietary habits: Not investigated GI symptoms: FGID in all ASD and some NT	16S rRNA sequencing (Rectum mucosa samples) Alpha diversity Not investigated Beta diversity ASD-FGID microbiota is different from NT-FGID or NT	Species ↑*Clostridium lituseburense*, *Lachnoclostridium bolteae, Lachnoclostridium hathewayi* *Clostridium aldenense Flavonifractor plautii* In ASD-FGID compared to NT-FGID and NT ↑*Turicibacter sanguinis* *C. aldenense* *C. lituseburense* *F. plautii* *C. disporicum* *C. tertium* In ASD with pain compared to ASD without pain, NT with and without pain	Species ↓*Dorea formicigenerans* *Blautia luti* *Sutterella species*	Microbial/host metabolites: ↓tryptophan in ASD-FGID compared with NT with/without FGID ↑5-HIAA levels in ASD-FGID compared to NT; this increase was associated with visceral pain Immune/inflammatory markers: ↓GROα, IFN-a2 ↑MCP-1 and eotaxin levels in the blood of ASD-FGID compared with NT with/without FGID Positive correlations: ↑in MCP-1 and eotaxin with abdominal pain in ASD-FDIG *C disporicum* and MIP-1a, MIP-1β, VEGF, IFN-g, IL-12p70, IL-17A, IL-5, IL-6, IP-10\ *C. tertium* and IL-1RA, IFN-g, IL-12p70, IL-17A, IL-1a, IL-5, IL-6, MIP-1a, MIP-1b	Not investigated

ADHD, attention deficit hyperactivity disorder; ASD, Autism Spectrum Disorder; ASD-GI, Autism Spectrum Disorder with GI symptoms; CASP, Critical Appraisal Skills Program; CNS, central nervous system; FGID, functional gastrointestinal disorders (according to ROME III criteria); GABA, Gamma-aminobutyric acid; GI, gastrointestinal; GROα, growth-related oncogene alpha; 5-HIAA, hydroxyindoleacetic acid; IL, Interleukin; IFN, Interferon; IgA, Immunoglobulin A; IgG, Immunoglobulin G; KEGG, Kyoto Encyclopedia of Genes and Genomes; MIA, Maternal immune activation; NMDA, N-methyl-D-aspartate; MCP-1, monocyte chemoattractant protein; MIP, macrophage inflammatory protein; NT, neurotypical controls; NT-GI, neurotypical with GI symptoms; No-GI, no GI symptoms; NOD, Nucleotide-binding oligomerization domain; PBMC, Peripheral blood mononuclear cells; qPCR, quantitative polymerase chain reaction; PD, Parkinson’s Disease; PHA, phytohemagglutinin (a T-cell activator); PICRUST, Phylogenetic Investigation of Communities by Reconstruction of Unobserved States; rRNA, ribosomal ribonucleic acid; TGF, Transforming growth factor; Th-17, T helper 17 cells; TLR4, Toll-like receptor 4; TNF, Tumor necrosis factor; VEGF, vascular endothelial growth factor. *Ruminococcaceae renamed to Oscillospiraceae in 2019.

**Table 2:** Evidence of microbiota alterations in ASD compared to NT siblings.

Study characteristics	Microbiota assessment	Increasing microbial taxa	Decreasing microbial taxa	Metabolites/Immune markers	Functional pathways
Son et al. (2015) Case–control (CASP Score: 14) Sample size: *N* = 103 Age range (years): 7-14 Dietary habits: Documented; included in the analysis GI symptoms: ↑pain and constipation in ASD-FGID symptoms in ASD and NT (4 groups)	16S rRNA sequencing (Stool samples) Alpha diversity No differences Beta diversity No differences	↑*Cyanobacteria/Chloroplast* in ASD-GI compared to ASD-No-GI, NT-GI, and NT-No-GI siblings (probably due to chia seeds intake in two ASD-GI with constipation)	No decrease was observed	Not investigated	Not investigated
Gondalia et al. (2012) Case–control (CASP Score: 13) Sample size: *N* = 104 Age range (years): 2-12 Dietary habits: Not investigated GI symptoms: ↑GI symptoms in ASD	GS FLX Titanium sequencing platform (Roche®, USA) (Stool samples) Alpha diversity No differences Beta diversity No differences	No increase was observed	No decrease was observed	Not investigated	Not investigated
De Angelis et al. (2013) Case–control (CASP Score: 20) Sample size: *N* = 30 Age range (years): 4-10 Dietary habits: Not investigated GI symptoms: Not investigated	16S rRNA sequencing (bTEFAP) and bacterial cultures (Stool samples) Alpha diversity ↑ number of observed species and evenness (Chao1 and Shannon indexes) in ASD and PDD-NOS Beta diversity Microbiota composition is different between ASD, PDD-NOS and NT	Phylum ↑Bacteroidetes in ASD ↑active Firmicutes in PDD-NOS Genus *In ASD*: ↑*Clostridium* *Roseburia* *Caloramator* *Sarcina* *Akkermansia* *Shigella* *In PDD-NOS:* ↑*Ruminococcus* *Collinsella* *Faecalibacterium* *In ASD and PDD-NOS:* ↑*Anaerofilum* *Dorea* *Caloramator* *Bacteroides* in ASD (and species *B. fragilis, B. vulgatus*) *Alistipes*	Phylum ↓total and active Firmicutes in ASD ↓Fusobacteria, Verrucomicrobia in ASD and PDD-NOS Genus In *ASD:* ↓*Faecalibacterium* (and species *F. prausnitzii*) *Prevotella* species *Bifidobacterium* (and species *B.* sp. and *B. adolescentis*) *Collinsella* *In PDD-NOS:* ↓*Coprococcus* *Lachnospira* in *Prevotella* (and both species *P.* sp. And *P. copri*) *In ASD and PDD-NOS:* ↓*Oscillospira* *Sporobacter* *Subdoligranulum* *Escherichia* *Fusobacterium*	Microbial-derived metabolites: *Free amino acids (FAA):* ↑total FAA in ASD compared to NT and PDD-NOS ↑Glu, Ala, Asp., Lys, Val, Ile, Phe, His, Trp, Lys and Pro in ASD and PDD-NOS compared to NT Volatile organic compounds (i.e., aldehydes, esters, sulfur compounds) in stool samples are different in ASD compared to PDD-NOS and NT *SCFAs:* ↓total medium and SCFAs in PDD-NOS and ASD ↑acetic and propionic acids in ASD and PDD-NOS Positive correlations: *Clostridium* species and methyl esters (of the butanoic acid, acetic acid and pentanoic acid) and indoles *Faecalibacterium, Ruminococcus* and *Bifidobacterium* genera with total SCFAs	Not investigated
Pulikkan et al. (2018) Case–control (CASP Score: 17) Sample size: *N* = 54 Age range (years): 3-16 Dietary habits: Documented; not included in the analysis GI symptoms: ↑GI symptoms in ASD	16S rRNA sequencing (Stool samples) Alpha diversity No differences Beta diversity No differences between ASD and NT Correlations of the covariates showed autism as a significant component in differentiating the gut microbiome of ASD and NT ↑Firmicutes/Bacteroidetes ratio in ASD	Family ↑*Veillonelleaceae* *Lactobacillaceae* *Bifidobacteriaceae* *Erysipelotrichaceae* *Enterococcaceae* *Desulfovibrionaceae* Genus ↑*Bifidobacterium* *Lactobacillus (and L. ruminis)* *Megasphaera* *Mitsuokella* *Ruminococcus* *Coprococcus* *Butyrivibrio* *Klebsiella*	Family ↓*Prevotellaceae,* Genus *↓Prevotella* *Faecalibacterium* *Roseburia*	Possible outcomes: ↑*Lactobacillus*- > potential inhibition of IDO1 which can affect the Th17 cell functioning leading to inflammatory conditions ↓*Faecalibacterium* and *Roseburia* - > potential reduction in the production of SCFAs	Not investigated
Ahmed et al. (2020) Case–control (CASP Score: 8) Sample size: *N* = 131 (NT are both siblings and unrelated-2 groups) Age range (years): 2-12 Dietary habits: Documented; not included in the analysis GI symptoms: 80% of ASD with GI symptoms	16S rRNA sequencing (Stool samples) Alpha diversity No differences Beta diversity No differences ↓Firmicutes/Bacteroidetes ratio in ASD and the NT siblings Firmicutes/Bacteroidetes ratio is different between ASD and NT unrelated Firmicutes/Bacteroidetes ratio is not different between ASD and NT siblings	Phylum ↑Firmicutes in severe ASD compared to moderate and mild Genus ↑*Bacteroides* *Ruminococcus* Species ↑*Bifidobacterium* sp. in NT siblings compared to ASD and NT unrelated	Phylum No decrease was observed Genus *↓Prevotella* *↓Prevotella* to *Bacteroides* Ratio in ASD and NT siblings compared to NT unrelated Species No decrease was observed	Not investigated	Not investigated
Yap et al. (2021) Case–control (CASP Score: 18) Sample size: *N* = 247 (NT are both siblings and unrelated-2 groups) Age range (years): 2-17 Dietary habits: Documented and included in the microbiota analysis: *Dietary diversity* ↓dietary alpha diversity in ASD compared to NT siblings and NT unrelated -Positive correlation between microbial taxonomic and dietary diversity in all the groups ↓dietary diversity is associated with ↓microbiome diversity in ASD but not in NT siblings or NT unrelated GI symptoms: *↓*stool consistency in ASD	Metagenomics sequencing (Stool sample) *Bacteria* Alpha diversity No differences Beta diversity No differences *Virome:* No differences	Species No increase was observed	Species ↓*Romboutsia timonensis* in ASD compared to NT siblings and NT unrelated	Not investigated	↓ microbial genes responsible for the metabolism of amino acids (L-glutamine, L-lysine, L-methionine, and L-threonine), purines and pyrimidines, carbohydrates (galactose), bacterial spore germination and dsDNA digestion in ASD compared to NT siblings and NT unrelated (MGPP against the MGENES) database

ASD, Autism Spectrum Disorder; ASD-GI, Autism Spectrum Disorder with GI symptoms; CASP, Critical Appraisal Skills Program; FGID, functional gastrointestinal disorders (according to ROME III criteria); GABA, Gamma-aminobutyric acid; GI, gastrointestinal; IDO1, indoleamine 2,3-dioxygenase; MGENES, Microba Genes database; MGPP, Microba Gene and Pathway Profiler; NT, neurotypical controls; NT-GI, neurotypical with GI symptoms; No-GI, no GI symptoms; PDD-NOS, Pervasive Developmental Disorder – Not Otherwise Specified; rRNA, ribosomal ribonucleic acid; SCFAs, short chain fatty acids; Th-17, T helper 17 cells; bTEFAP, Tag-encoded FLX amplicon pyrosequencing. *Ruminococcaceae renamed to Oscillospiraceae in 2019.

**Table 3:** Evidence of microbiota alterations in ADHD.

Study characteristics	Microbiota assessment	Increasing microbial taxa	Decreasing microbial taxa	Microbiota and ADHD symptoms	Functional pathways
Jiang et al. (2018) Cohort (CASP Score: 15) Sample size: *N* = 83 Age range (years): 6-10 Dietary habits: Documented; not included in the analysis GI symptoms: Not investigated	16S rRNA sequencing (Stool samples) Alpha diversity No differences Beta diversity No differences	Family ↑*Peptostreptococcaceae* *Moraxellaceae* *Xanthomonadaceae* *Peptococcaceae* Genus No increase was observed	Family ↓*Alcaligenaceae* Genus ↓*Faecalibacterium,* *Lachnoclostridium* *Dialister* *Sutterella*	Negative correlation between *Faecalibacterium* and severity of ADHD symptoms (CPRS score and the hyperactivity index score)	Not investigated
Prehn-Kristensen et al. (2018) Cohort (CASP Score: 19) Sample size: *N* = 31 Age range (years): 12-14 Dietary habits: Not investigated GI symptoms: Not investigated	16S rRNA sequencing (Stool samples) Alpha diversity No difference in the observed species (Chao1 index) and richness between ADHD and NT ↓richness and evenness (Shannon index) in ADHD (w/w or w/o medications) compared to NT ↓richness and evenness (Shannon index) in the mothers of ADHD compared to NT, no changes in the fathers Beta diversity ADHD microbiota is different from NT Mothers of ADHD children have different microbiota from ADHD children and NT children	Family ↑*Neisseriaceae, Bacteroidaceae* Genus ↑*Neisseria* frequency in ADHD (No difference in *Neisseria* abundance in ADHD)	Family ↓*Prevotellaceae, Catabacteriaceae*, *Porphyromonadaceae* Genus No decrease was observed	Negative correlation between the levels of hyperactivity and the reduction in alpha diversity in ADHD Two species belonging to the genera *Bacteroides* are associated with ADHD (OTU_7, OTU_577) Positive correlation between *Bacteroides* and levels of hyperactivity and impulsivity in ADHD	Not investigated
Wang et al. (2020) Cohort (CASP Score: 17) Sample size: *N* = 60 Age range (years): 6-16 Dietary habits: Documented and include in microbiota analysis: ADHD display ↑intake of refined grains and ↓dairy and vitamin B2 intake In ADHD: -*S. stercoricanis* had negative correlation to the intake of dairy, and a positive correlation with nuts/seeds/legumes as well as ferritin and magnesium intake -*B uniformis* was positive correlated to fat and carbohydrate intake. GI symptoms: Not investigated	16S rRNA sequencing (Stool samples) Alpha diversity ↑Shannon index (richness and evenness) and Chao1 index (species abundance) ↓Simpson index (number of species and relative abundance of each species) in ADHD Beta diversity No differences	Phylum ↑Fusobacteria Genus ↑*Fusobacterium* Species ↑*Bacteroides uniformis* *Bacteroides ovatus* *Sutterella stercoricanis*	Phylum No decrease was observed Genus ↓*Lactobacillus* Species ↓*Bacteroides coprocola*	Positive correlation between *B. ovatus* and *S. stercoricanis* with ADHD clinical symptoms Possible outcomes Potential association between *Bacteroides* sp. and the development of the frontal lobe, cerebellum, and hippocampus	Not investigated
Wan et al. (2020) Case–control (CASP Score: 20) Sample size: *N* = 34 Age range (years): 6-12 Dietary habits: Documented; not included in the analysis GI symptoms: Not investigated	Metagenomic sequencing (Stool samples) Alpha diversity No differences Beta diversity No differences	Family ↑*Odoribacteraceae Enterococcaceae* Genus ↑*Odoribacter* *Enterococcus* Species ↑*Bacteroides caccae Odoribacter splanchnicus Paraprevotella xylaniphila Veillonella parvula*	Family ↓*Veillonellaceae* Oscillospiraceae* Genus ↓*Faecalibacterium* Species *↓Faecalibacterium prausnitzi* *Lachnospiraceae bacterium Ruminococcus gnavus*	Not investigated	↑enzymatic pathways involved in dopamine turnover at the postsynaptic neuron; the genes encoding the catalytic subunit of PP1, threonine synthase, and 6-pyruvoyl-5,6,7,8-tetrahydropterin *↓*gene encoding 4-hydroxy threonine-4-phosphate dehydrogenase (PICRUSt-KEGG database)
Richarte et al. (2021) Case–control (CASP Score: 13) Sample size: *N* = 200 Age range (years): 22-44 Dietary habits: Not investigated GI symptoms: Not investigated	16S rRNA sequencing (Stool samples) Alpha diversity No differences Beta diversity No differences	Family ↑*Selenomonadaceae Veillonellaceae* Genus ↑*Dialister* *Megamonas*	Family ↓*Gracilibacteraceae* in ASD-GI (compared to NT-GI) Genus ↓*Gracilibacter* *Anaerotaenia*	Positive correlation between *Anaerotaenia* and *Gracilibacter* in ADHD Negative correlation between *Anaerotaenia* and *Megamonas* in ADHD	Not investigated
Aarts et al. (2017) Cohort (CASP Score: 19) Sample size: *N* = 96 (NT are siblings and unrelated and are considered as one group) Age range (years): 18-21 Dietary habits: Not investigated GI symptoms: Not investigated	16S rRNA sequencing (Stool samples) Alpha diversity Not investigated Beta diversity Not investigated	Phylum ↑Actinobacteria Class ↑Bacteroidia Deltaproteobacteria Order ↑Bacteriodales Bifidobacteriodales Coriobacteriales Desulfovibrionales Family ↑*Rikenellaceae* *Bacteroidaceae* *Bifidobacteriaceae* *Coriobacteriaceae* Genus ↑*Bifidobacterium* *Eggerthella* *Bacteroides* *Odoribacter* *Alistipes* *Parabacteroides* Species ↑*Bacteroides uniformis Bacteroides ovatus Bacteroides vulgatus* *B. longum* *B. adolescentis*	Phylum No decrease was observed Class ↓Clostridia Order ↓Eubacteriales^#^ Family ↓Oscillospiraceae* Lachnospiraceae Genus ↓*Ruminococcus* *Acetivibrio* *Coprococcus* *Subdoligranulum* Species No decrease was observed	Negative correlation between the abundance of predicted CDT and reward anticipation, a key symptom in ADHD and dopamine target	↑CDT in the bacteriome from ADHD compared to NT Positive correlation between ↑CDT and *Bifidobacterium* in ADHD Possible outcomes: ↑CDT- > ↑phenylalanine, an essential AA that crosses the BBB and acts as a precursor for dopamine and noradrenaline (PICRUSt-KEGG database)
Szopinska-Tokov et al. (2021) Cohort (CASP Score: 13) Sample size: *N* = 89 (NT are siblings and unrelated and are considered as one group) Age range (years): 13-29 Dietary habits: Not investigated GI symptoms: Not investigated	16S rRNA sequencing (Stool samples) Alpha diversity No differences Beta diversity No differences	*In ADHD compared to NT* Genus ↑*Intestinibacter* (low relative abundance in both groups) *In ADHD-medicated compared to ADHD-naïve* Genus No increase was observed	*In ADHD compared to NT* Genus ↓*Coprococcus* *Prevotella* *In ADHD-medicated compared to ADHD-naïve* Genus ↓*Lactobacillus* *Lachnospiraceae_ND3007* *Ruminococcaceae_ g* *Ruminococcaceae_UCG.014*	Correlation between *Coproccocus_2* with ADHD symptoms, specifically inattention symptoms (*non-significant*)	Not investigated

AA, amino acids; ADHD, attention deficit hyperactivity disorder; BBB, Blood Brain Barrier; CASP, Critical Appraisal Skills Program; CDT, cyclohexadienyl dehydratase; CPRS, Conner’s Parent Rating Scales; GI, gastrointestinal; KEGG, Kyoto Encyclopedia of Genes and Genomes; NT, neurotypical controls; OTUs, Operational taxonomic unit; PICRUST, Phylogenetic Investigation of Communities by Reconstruction of Unobserved States; PP1, protein phosphatase-1; rRNA, ribosomal ribonucleic acid. *Ruminococcaceae was renamed to Oscillospiraceae; #Clostridiales was amended in Eubacteriales in 2019.

**Table 4:** Evidence of microbiota alterations in Rett syndrome.

Study characteristics	Microbiota assessment	Increasing microbial taxa	Decreasing microbial taxa	Metabolites/Immune markers	Functional pathways
Strati et al. (2016) Cohort (CASP Score: 20) Sample size: *N* = 79 Age range (years): 5-26 Dietary habits: Not investigated GI symptoms: ↑constipation and inflammation (↑fecal calprotectin and ESR) in RETT	16S rRNA sequencing Fungal ITS1 rDNA region (Stool samples) Bacterial diversity Alpha diversity ↓bacterial richness (Shannon index) and species abundance (Chao1 index) RETT-C and RETT-NC Beta diversity RETT microbiota is different from NT No differences between RETT-C and RETT-NC ↑Firmicutes/Bacteroidetes ratio Fungal diversity Alpha diversity No differences Beta diversity RETT mycobiota is different from NT No differences between RETT-C and RETT-NC	Phylum (Bacteria) ↑Actinobacteria Genus (Bacteria) ↑*Bifidobacterium* *Escherichia-Shigella* *Actinomyces* *Clostridium XIVa Anaerostipes* *Lactobacillus* *Blautia* *Eggerthella* *Enterococcus* *Erysipelotrichaceae incertae sedis* *Megasphaera* Species (Bacteria) ↑*Bifidobacterium longum* Genus (Fungi) ↑*Candida*	Phylum (Bacteria) No decrease was observed Genus (Bacteria) ↓*Bacteroides* *Faecalibacterium* *Gemmiger* *Ruminococcus* *Biophila* Species (Bacteria) No decrease was observed Genus (Fungi) No decrease was observed	↑overall content of SCFAs (propionate, isovalerate/2-methylbutyrate, isobutyrate) in RETT Possible outcomes: Non-physiological high levels of SCFAs in the gut could contribute to the constipation status observed in RETT Non-physiological high levels of SCFAs affect gene expression, brain function and behavior, neurotransmitter systems, neuronal cell adhesion, inflammation, oxidative stress, lipid metabolism and mitochondrial function	↑carbohydrate and propanoate metabolism in the gut microbiota of RETT subjects (same pathways of SCFAs production) in RETT (PICRUSt-KEGG database)
Thapa et al. (2021) Case–control (CASP Score: 17) Sample size: *N* = 65 Age range (years): 5-36 Dietary habits: Documented and included in microbiota analysis GI symptoms: ↑GI symptoms in RETT	16S rRNA sequencing (Stool samples) Alpha diversity *Between RETT and NT* No differences *Between RETT subgroups* ↓in RETT pre-P vs. post-P ↓in RETT-severe vs. mild/moderate ↑in RETT solid food vs. formula Beta diversity *Between RETT and NT* No differences *Between RETT subgroups* Microbiota is different between: -RETT pre-P and post-P -RETT severe and mild/moderate -RETT food- and formula-fed	Genus ↑*Bifidobacterium* in RETT and NT formula-fed	Genus No decrease was observed	Microbial-derived metabolites: ↓fecal GABA and tyrosine concentrations in RETT compared to NT ↓fecal glutamate (*not significant*) in RETT compared to NT No changes in fecal tryptophan and glutamine between RETT and NT	Not investigated
Borghi et al. (2017) Case–control (CASP Score: 16) Sample size: *N* = 18 Age range (years): 15-30 Dietary habits: Documented; not included in the microbiota analysis GI symptoms: ↑constipation in RETT	16S rRNA sequencing (Stool samples) Alpha diversity ↓in RETT compared to NT ↓in RETT severe compared to mild/moderate *(not significant)* Beta diversity RETT microbiota is different from NT RETT severe is different from mild/moderate *(not significant)*	*Between RETT and NT* Family ↑*Bacteroidaceae* Genus ↑*Bacteroides* Species ↑*Clostridium* sp. *Sutterella* sp. *Between RETT subgroups* Family ↑*Bacteroidaceae*, *Enterobacteriaceae* *Erysipelotrichaceae* (In RETT mild compared to severe)	*Between RETT and NT* Family ↓Oscillospiraceae* Genus No decrease was observed Species ↓*Faecalibacterium prasunitzii* *Prevotella* sp. *Roseburia* sp. *Between RETT subgroups* Family ↓*Oscillospiraceae* (In RETT mild compared to severe)	Microbial-derived metabolites: No difference in total SCFAs and acetate between RETT and NT ↑Butyrate and propionate in RETT compared to NT ↑BCFAs in RETT compared to NT *In RETT and NT:* Positive correlation between: *Parabacteroides* and propionate, butyrate, and BCFA concentrations *Alcaligenaceae* and propionate *Porphyromonadaceae* and propionate, butyrate, and BCFA *Ruminococcaceae* and BMI *Bacteroides* and *Clostridium* and total protein and animal protein intake Negative correlation between: *Bacteroidaceae*, *Bacteroides* and total SCFAs and acetate *Bacteroidaceae*, *Bacteroides*, *Veillonaceae* and BMI	In RETT compared to NT ↓enzymes for carbohydrate and lipid metabolism ↑amino acids pathway ↑butanoate and propanoate metabolism (PICRUSt-KEGG database)

BCFA, branched chain fatty acids; BMI, Body mass index; CASP, Critical Appraisal Skills Program; GABA, Gamma-aminobutyric acid; GI, gastrointestinal; ESR, erythrocyte sedimentation rate; KEGG, Kyoto Encyclopedia of Genes and Genomes; NT, neurotypical controls; PICRUST, Phylogenetic Investigation of Communities by Reconstruction of Unobserved States; rRNA, ribosomal ribonucleic acid; RETT, Rett’s syndrome; RETT-C, subjects with Rett’s syndrome and constipation; RETT-NC subjects with Rett’s syndrome and without constipation; RETT pre-P, subjects with Rett’s syndrome in their pre-puberty period; RETT post-P, subjects with Rett’s syndrome in their post-puberty period; SCFAs, short chain fatty acids. *Ruminococcaceae renamed to Oscillospiraceae in 2019.

Studies examining the effects of microbiota- or diet-targeted interventions on behavioral outcomes and microbiota composition in subjects with neurodevelopmental disorders are summarized (Table 5; Supplementary Table S9). Two studies assessed the effect of probiotic administration (Tomova et al., 2015; Shaaban et al., 2018), and one study explored the effects of FMT (Kang et al., 2017) on gut microbiota, GI symptoms, and neurodevelopmental outcomes in individuals with ASD. One study assessed the effect of dietary micronutrient supplementation on gut microbiota composition and behavioral symptomatology in subjects with ADHD (Stevens et al., 2019). One study assessed the effect of perinatal administration of probiotic *Lactobacillus rhamnosus* GG in preventing the onset of ASD or ADHD in childhood (Pärtty et al., 2015). The characteristics of subjects included in the intervention trials are summarized (Supplementary Table S9).

**Table 5:** Intestinal microbiota- or diet-targeted interventions in neurodevelopmental disorders.

Study characteristics	Intervention	Effect on the microbiota	Effect on disease severity	Effect on GI function	Other effects
Kang et al. (2017) Case–control/open label (CASP Score: 14) Sample size: *N* = 38 Age range (years): 7-16 Dietary habits: Documented; not included in the analysis GI symptoms: ↑GI symptoms in ASD	FMT Standardized Human Gut Microbiota, high and low dose, orally or rectally administered for 8 weeks- 8 weeks of follow up. ASD children were subjected to FMT, NT children were not subjected to FMT	16S rRNA sequencing (Stool sample) Bacterial diversity ↑bacterial diversity ↑*Bifidobacterium* *Desulfovibrio* *Prevotella* In ASD children following FMT Virus diversity No changes in phage richness and evenness in ASD after FMT Positive engraftment of phageome from the donor to the ASD and increased abundance of phage after FMT Overall shift of ASD microbiota to NT after FMT	FMT improved ASD symptoms of: social skill deficits, irritability, hyperactivity, lethargy, stereotypy, and aberrant speech adaptive behaviors such as communication, daily living skills, socialization	FMT improved GI symptoms in ASD children (visceral pain, constipation, indigestion, diarrhea) GI improvements lasted after 8 weeks of no treatment (follow up)	Not investigated
Shaaban et al. (2018) Case–control (CASP Score: 15) Sample size: *N* = 60 Age range (years): 5-9 Dietary habits: Not investigated GI symptoms: ↑ GI symptoms in ASD	Probiotic treatment *Lactobacillus acidophilus*, *Lactobacillus rhamnosus*, *Bifidobacteria longum* Dose: 1 × 10^6^ colony forming units/g Frequency: oral administration, once daily for 3 months	RT-PCR (stool samples) Bacterial diversity Not investigated Genus ↓*Bifidobacteria* in ASD ↑*Bifidobacteria* *Lactobacillus* in ASD treated with probiotics	↓ASD severity after probiotic treatment In ASD children, probiotics improved: Speech Language Communication Sociability Sensory/cognitive awareness Health and physical behavior	↓constipation, flatulence, visceral pain in ASD children treated with probiotics Positive correlation between improvement of GI symptoms and reduction of ASD severity in ASD children treated with probiotics	↓body weight and BMI in obese ASD children treated with probiotics Negative correlation between the ↑*Bifidobacteria* due to probiotics treatment with the decrease of body weight after the probiotic’s treatment
Tomova et al. (2015) Case–control (CASP Score: 20) Sample size: *N* = 20 (NT are siblings and unrelated-2 groups) Age range (years): 2-17 Dietary habits: Not investigated GI symptoms: ↑GI symptoms in ASD and their non-autistic siblings (compared to NT)	Probiotic treatment (*L. casei* and *B. longum*) in ASD children: Dose: Dietary supplementation of one capsule of “Children Dophilus” containing 3 strains of *Lactobacillus* (60%), 2 strains of *Bifidobacteria* (25%) and one strain of *Streptococcus* (15%) Frequency: oral administration, three times a day for 4 months	Targeted qPCR (Stool sample) Bacterial diversity *Before intervention* See Supplementary Table S9 *After intervention* ↑Bacteroidetes/Firmicutes ratio to levels compared to NT unrelated Normalization of *Bifidobacterium* and *Lactobacillus* to levels of NT (both) ↓*Desulfovibrio* to levels of NT (both)	The effect of the probiotic treatment on ASD severity was not documented	The effect of the probiotic treatment on GI symptoms in ASD was not documented	↓fecal TNFα in ASD children supplemented with probiotic (before the intervention fecal TNFα was found ↑ in ASD children and their siblings)
Pärtty et al. (2015) RCT, double blind, placebo prospective follow-up (CASP Score: 20) Sample size: *N* = 75 Age range (years): 0-13 Dietary habits: Documented; not included in the analysis GI symptoms: Not investigated	Probiotic treatment Perinatal supplementation *Lactobacillus rhamnosus GG* *During pregnancy:* Dose: 1×10^10^ colony-forming units of *Lactobacillus rhamnosus GG* or placebo (microcrystalline cellulose) Frequency: daily for 4wk before expected delivery *After birth:* Dose: 1×10^10^ colony-forming units of *Lactobacillus rhamnosus GG* or placebo (microcrystalline cellulose) to the children up to 6 months Frequency: daily (if breastfeeding, the supplementation was given to the mothers)	FISH assay and qPCR No difference in bacterial number (assessed with FISH) in 3 months children that later will develop AS or ADHD and NT Genus ↓*Bifidobacterium* in 6 months children that later will develop AS, or ADHD compared with NT (assessed with FISH) ↓*Bacteroides* and *Lactobacillus-Enterococcus* in 18 months children that later will develop AS, or ADHD compared with NT (assessed with FISH) Species ↓*Bifidobacterium longum* in 3 months children that later will develop AS, or ADHD compared with NT (assessed by qPCR) ↓*Clostridium histolyticum* in 24 months children that later will develop AS, or ADHD compared with NT (assessed with FISH) No difference in bacterial number (assessed with FISH) in 13 years children with AS or ADHD compared with NT	Potential effect of *Lactobacillus rhamnosus* GG on the gut-brain axis: In modulating emotional behavior and the central GABAergic system through the vagus nerve, all features associated with neuropsychiatric disorders	Potential effect of *Lactobacillus rhamnosus* GG on gut health: In stabilizing gut barrier permeability by modulating the tight junctions In regulating mucin production and antigen-specific IgA production	Not investigated
Stevens et al. (2019) RCT, double blind (CASP Score: 19) Sample size: *N* = 18 Age range (years): 7-12 Dietary habits: Dietary patterns, including consumption of fruit, vegetables, breakfast, and fast foods, were assessed at the baseline and end of RCT with a higher score indicative of a healthier eating pattern GI symptoms: Not investigated	Micronutrient supplementation Dose: capsules (formulation containing a blend of vitamins, minerals, amino acids and antioxidants-ADHD-t) or placebo to ADHD subject (ADHD-p) Frequency: one capsule, three times each day, increasing the dose by three capsules every two days up to a target dose of 12 capsules per day: four taken at three different intervals For each participant, a pre-RCT, and a post-RCT fecal sample was collected and sequenced for microbiota analysis	16S rRNA sequencing (Stool sample) Bacterial diversity Alpha diversity ↑observed OTUs in ADHD-t compared to ADHD-p (no changes between pre- and post-RCT) ↓Shannon diversity in ADHD-p between pre- and post- RCT Beta diversity No differences between ADHD-t and ADHD-p, or pre- and post- RCT In ADHD-t (*vs* ADHD-p) Phylum ↓Actinobacteria ↑Proteobacteria Order ↓Bifidobacteriales ↑Coriobacteriales Genera ↓*Bifidobacterium* sp. ↓*B. longum* ↑*Collinsella aerofaciens*	In ADHD-t post RCT, a low abundance of *Bifidobacterium* is associated with a low ADHD-IV-RS score, which is contradictory to the general trend observed in the pre-RCT and ADHD-p (*Trend, not significant*) Possible outcomes: Potential interaction between macronutrient (diet) and micronutrient (intervention) intake with the behavioral outcomes observed in ADHD subjects	Not investigated	Not investigated

ADHD, attention deficit hyperactivity disorder; ADHD-IV-RS, ADHD rating scale (IV edition); ASD, Autism Spectrum Disorder; AS, Asperger’s Syndrome; BMI, Body mass index; CASP, Critical Appraisal Skills Program; FISH, Fluorescence In Situ Hybridization; FMT, Fecal Microbiota Transplantation; GABA, Gamma-aminobutyric acid; GI, gastrointestinal; NT, neurotypical controls; RT-PCR, real time polymerase chain reaction; qPCR, quantitative polymerase chain reaction; RCT, randomized control trial; rRNA, ribosomal ribonucleic acid; TNFα, Tumor necrosis factor α.

### Sample harvesting and analysis

Samples used in the selected studies were derived from stool (31 studies), rectal tissue (1 study), and duodenal tissue (1 study). Samples from seven studies were stored at 4°C for ≤24 h before being frozen for storage (Pärtty et al., 2015; Kantarcioglu et al., 2016; Aarts et al., 2017; Prehn-Kristensen et al., 2018; Stevens et al., 2019; Wang et al., 2020; Szopinska-Tokov et al., 2021). All other studies’ samples were frozen immediately upon collection. Samples from five studies were stored with preservation agents at the time of collection (De Angelis et al., 2013; Son et al., 2015; Rose et al., 2018; Richarte et al., 2021; Thapa et al., 2021). Further details about DNA extraction, sequencing technique, reference database and sequence corrections are described (Supplementary Table S10).

### Neurodevelopmental outcome measurements

Measurements of neurodevelopmental outcomes in ASD, ADHD, and RETT are summarized in Supplementary Tables S5–S9.

## Characteristics of the intestinal microbiota in ASD

### Changes in bacterial diversity in ASD

Ten studies compared stool-associated bacterial communities of ASD subjects to NT controls (with no first-degree family relationships to ASD subjects, Table 1), while four studies used NT first-degree siblings of ASD subjects, and two studies included both NT first-degree siblings of ASD subjects and unrelated NT controls (Table 2). Bacterial α-diversity indices were evaluated in five of the 10 studies in which NT controls were unrelated to ASD individuals. Four studies reported reduced, and one study reported increased bacterial α-diversity of ASD-associated microbiota (Table 1). Six of the 10 studies, in which NT controls were unrelated to ASD individuals, evaluated β-diversity indices and showed differences in gut bacterial profiles between ASD subjects and NT controls (Table 1).

Conflicting α- and β-diversity outcomes occurred in three of the four studies in which NT controls were siblings of ASD children as no differences in bacterial α-diversity or composition (β-diversity) were found between groups in these studies (Table 2). No differences in α- or β-diversity were again reported by Yap et al. (2021) and Ahmed et al. (2020), who compared bacterial α- and β-diversities of ASD subjects with both NT siblings and unrelated NT controls. De Angelis et al. (2013) reported significantly increased α-diversity in both ASD and PDD-NOS subjects, and differences in relative abundance of bacterial communities between subjects with ASD, PDD-NOS, and their NT first-degree siblings (Table 2).

The two studies evaluating mucosal biopsy-associated microbiota included only unrelated NT controls (Table 1). Kushak et al. (2017) did not report any differences in bacterial diversity of duodenal mucosa-associated microbiota between ASD subjects and NT controls (Kushak et al., 2017). Luna et al. (2017) reported differences in β-diversity between rectal mucosa-associated microbiota of ASD and NT controls (Luna et al., 2017).

Altered bacterial diversity in ASD children compared to unrelated NT controls might suggest the presence of an ASD-related gut microbiota phenotype (Brito et al., 2019). However further studies with larger sample sizes and with both siblings and family unrelated NT controls are warranted to determine whether children with ASD have an altered microbiota due to the disease.

### Changes in the relative abundance of bacterial taxa in ASD

Multiple studies reported distinctive alterations in bacterial taxa among subjects with ASD; however, these results were not consistent. A reduction in the relative abundance of the genus *Prevotella* in ASD was reported across five studies assessing stool-associated microbiota (two studies included NT siblings), and in the study by Kushak et al. (2017), which investigated duodenal mucosa-associated microbiota (Tables 1, 2). The results from bacterial cultures in the study by De Angelis et al. (2013), reported increased relative abundance of *Prevotella* in ASD compared to NT subjects (Supplementary Table S6). *Faecalibacterium* and *Streptococcus* levels were decreased in ASD subjects in three (De Angelis et al., 2013; Kang et al., 2018; Pulikkan et al., 2018) and four studies (De Angelis et al., 2013; Kushak et al., 2017; Kang et al., 2018; Zhang et al., 2018), respectively (Tables 1, 2). Bacterial culture, 16S rDNA sequencing, and targeted PCR revealed higher levels of genus *Clostridium* in stool samples of ASD patients in the studies by De Angelis et al. (2013) (Table 2; Supplementary Table S6) and Tomova et al. (2015) (Table 5; Supplementary Table S9). In ASD-associated microbiota, *Lactobacillus* was increased in three studies (Tomova et al., 2015; Strati et al., 2017; Pulikkan et al., 2018) and decreased in De Angelis et al. (2013) (Tables 1, 2, 5). Three studies reported increased abundance of genus *Bacteroides* in ASD stool samples (De Angelis et al., 2013; Zhai et al., 2019; Ahmed et al., 2020), while Kushak et al. (2017) showed decreased abundance of *Bacteroides* in duodenal mucosa-associated microbiota from ASD subjects (Tables 1, 2).

At the species level, increased *Bacteroides fragilis* and *B. vulgatus* (De Angelis et al., 2013), *Clostridium* sp. (De Angelis et al., 2013; Finegold et al., 2017)., *Lactobacillus* sp. (Tomova et al., 2015). and reduced *Faecalibacterium prausnitzii* (De Angelis et al., 2013), *Romboutsia timonensis* (Yap et al., 2021), *Prevotella* sp. (De Angelis et al., 2013) were detected in ASD-associated microbiota (Tables 1, 2). Conflicting results were reported for *Bifidobacterium* abundance in ASD subjects compared to NT first-degree siblings. Decreased and increased abundance of the genus *Bifidobacterium* in ASD subjects compared to NT siblings were reported by De Angelis et al. (2013) and Pulikkan et al. (2018), respectively (Table 2). At the species level, reduced *B. adolescentis*, and *Bifidobacterium* sp. were reported by De Angelis et al. (2013) and Ahmed et al. (2020) (Table 2), whereas Tomova et al. (2015) observed an increased abundance of *Bifidobacterium* sp. in their cohort of ASD children (Table 5; Supplementary Table S9). In all studies there was no difference in the abundance of *Bifidobacterium* between children with ASD and unrelated NT subjects.

In mucosa-associated microbiota from duodenal biopsies of subjects with ASD, significant alterations occurred at the genus level, with reduced abundance of *Prevotella*, *Bacteroides*, *Streptococcus*, *Neisseria*, and increased abundance of *Devosia*, *Oscillospira*, *Actinomyces*, and *Peptostreptococcus*. These findings recapitulate some of the changes observed in the stool-associated microbiota from ASD subjects (Kushak et al., 2017). Positive correlations between *Bacteroides*, *Faecalibacterium*, and *Clostridium* and the activity of duodenal enzymes such as maltase, lactase and sucrase were also observed in ASD (Kushak et al., 2017; Table 1).

The study of Luna et al. (2017) reported increased abundance of bacterial species from Clostridiales in rectal mucosa-associated microbiota from ASD subjects. In particular, bacterial species *Clostridium lituseburense*, *Lachnoclostridium bolteae*, *Lachnoclostridium hathewayi*, *Clostridium aldenense*, and *Flavonifractor plautii* (Table 1), were significantly more abundant in ASD microbiota compared to NT controls. This study also observed a decrease in *Dorea formicigenerans* and *Blautia luti* (Table 1) as well as reduced *Sutterella species* (Table 1) in ASD-associated microbiota. Together these data showed major perturbations in the abundance of bacterial genera and species belonging to the phyla Firmicutes and Bacteroidetes in ASD-associated microbiota.

### Changes In bacterial functional profile in ASD

Few studies assessed changes in inferred bacterial function using 16S profiles and Phylogenetic Investigation of Communities by Reconstruction of Unobserved States (PICRUSt) to predict the presence of Kyoto Encyclopedia of Genes and Genomes (KEGG) orthologs across functional and metabolic pathways (Box A1; Kanehisa, 2000). Zhang et al. (2018) observed significant enrichments of several bacterial genes associated with D − Arginine and D − ornithine metabolism, ether lipid metabolism, bacterial chemotaxis, phosphotransferase system (PTS) and flagellar assembly genes in ASD subjects. Steroid hormone biosynthesis, glycosaminoglycan degradation and lipoic acid metabolism were more abundant in NT controls (Zhang et al., 2018; Table 1). Conversely, Kang et al. (2018) assessed genes in the metabolic pathways associated with p-cresol, vitamin K, and GABA but did not find any significant differences between ASD subjects and NT controls (Table 1). The study by Rose et al. (2018) differentiated NT or ASD subjects with GI symptoms (NT-GI and ASD-GI, respectively), compared with NT or ASD without GI symptoms (NT-no-GI and ASD-no-GI, respectively; Supplementary Table S5). Amino sugars and nucleotide sugars pathways related to the gut microbiota were significantly upregulated in ASD-GI compared to ASD-no-GI subjects. Starch and sucrose metabolism pathways were downregulated in ASD-no-GI subjects compared to ASD-GI subjects (Rose et al., 2018; Table 1). Zhai et al. (2019) described 40 metabolic pathways found to be statistically significantly different between subjects with ASD and NT controls. *Bacteroides, Oscillospira* and *Sutterella* were positively correlated with carbon fixation pathways in prokaryotes, the citrate cycle, and gas degradation (pathways downregulated in ASD), and negatively correlated with porphyrin and chlorophyll metabolism (pathways upregulated in ASD). Ether lipid metabolism and sporulation were upregulated in ASD and negatively associated with *Parabacteroides.* A negative correlation was found between *Bacteroides* and *Sutterella* and carotenoid biosynthesis metabolism (Table 1). Yap et al. (2021) assessed the potential functional profiling of ASD-associated microbiota by using the Microba Gene and Pathway Profiler (MGPP) tool against the Microba Genes (MGENES) database. The study assessed metabolic pathways associated with *Romboutsia timonensis*, as this species was found to be less abundant in ASD subjects. The predictive analysis showed reduced abundance of microbial genes responsible for the metabolism of amino acids (L-glutamine, L-lysine, L-methionine, and L-threonine), purines and pyrimidines, carbohydrates (galactose), bacterial spore germination and dsDNA digestion in ASD subjects compared to NT siblings and unrelated NT controls (Table 2). These results suggest potential alterations in the transcription of microbial genes associated with ASD. Further analyses (e.g., metatranscriptomics) will be required to evaluate protein expression associated with these microbial differences.

### Correlation between bacterial profiles and disease severity

**Figure 2:**
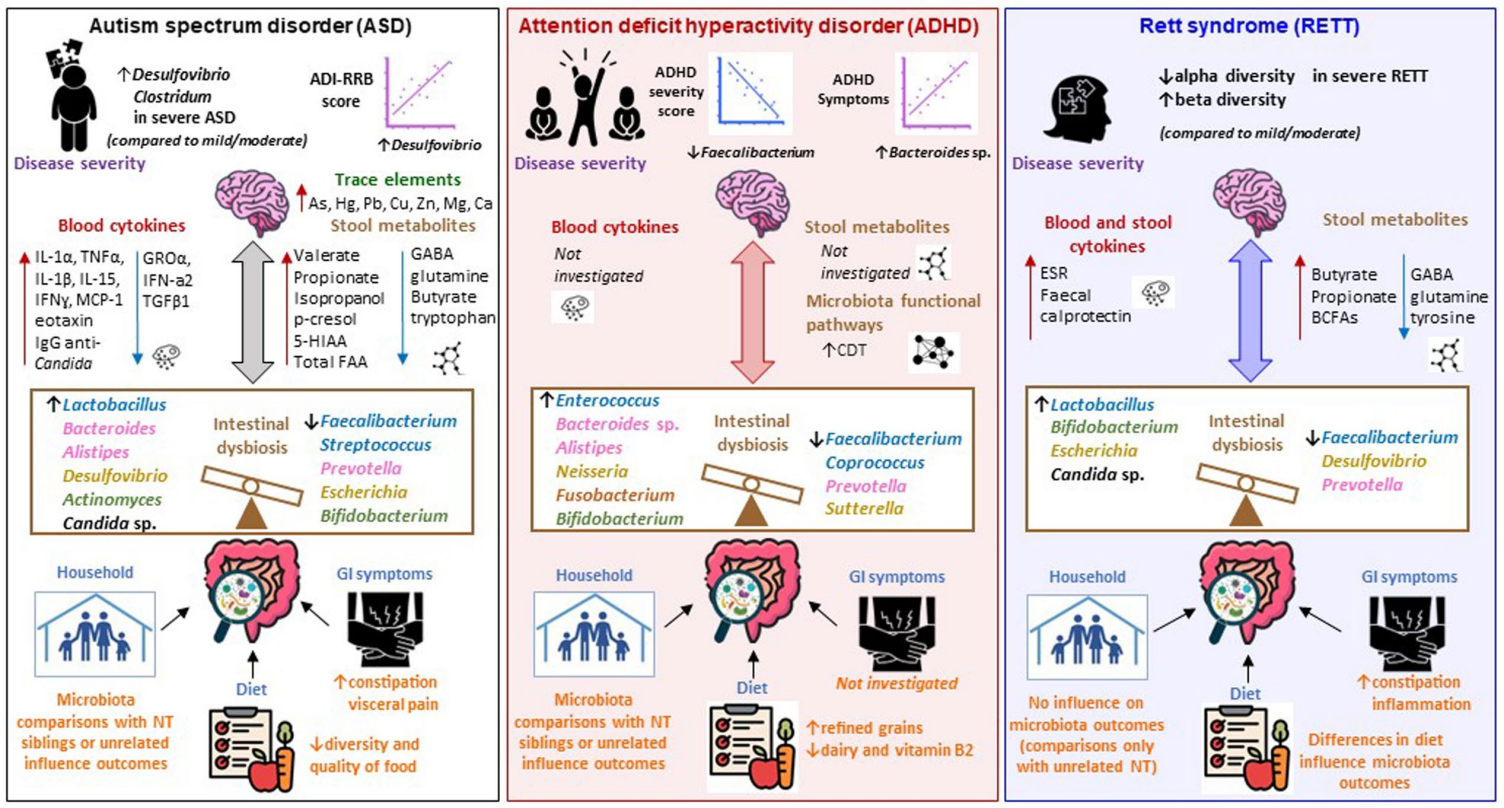
Microbiota-gut-brain axis alterations in ASD, ADHD, and Rett syndrome. Schematic representation of the main findings emerged from the studies included in our systematic review, portraited as alterations across the microbiota-gut-brain axis in ASD (left panel), ADHD (middle panel), and Rett’s syndrome (RETT; right panel). Changes in the intestinal microbiota of subjects with ASD are associated with a high prevalence of GI symptoms and low-quality diet. The family household might have an influence in shaping gut microbiota communities, as ASD and family unrelated NT subjects have a different microbiota, however no differences are observed when ASD subjects are compared with their siblings. RETT subjects present intestinal inflammation (indicated by increased ESR and fecal calprotectin) and constipation and there are changes in the gut microbiota depending upon the type of diet (solid food vs. formula). No information is provided on the presence of GI disorders in ADHD subjects, however differences in dietary habits between ADHD subjects and NT controls have been shown even though their impact on the gut microbiota has not been investigated. Intestinal dysbiosis is mainly characterized by decreased *Faecalibacterium* (phylum Firmicutes) and *Prevotella* (phylum Bacteroides) in all three disorders. Increased *Bacteroides* sp. (phylum Bacteroidetes) were identified in ASD and ADHD. *Lactobacillus* (phylum Firmicutes) was increased in ASD and RETT. *Bifidobacterium* (phylum Actinobacteria) increased in ADHD and RETT and decreased in ASD. *Desulfovibrio* (phylum Proteobacteria) increased in ASD and decreased in RETT. *Escherichia* (phylum Proteobacteria) increased in RETT and decreased in ASD. Increased abundances of *Candida* sp. were identified whithin the mycobiota of ASD and RETT. Microbiota associated metabolites such as SCFAs and gut-brain neurotransmitters GABA, glutamine, tryptophan, and tyrosine are altered in ASD and RETT, however they have not been explored in ADHD studies. Increased p-cresol, isopropanol in the stool and trace elements As, Hg, Pb, Cu, Zn, Mg, Ca in the hair were found in ASD subjects. ASD is also associated with a dysregulation of the immune system (and potential inflammation), as blood cytokines IL-1α, TNFα, IL-1β, IL-15, IFNɣ, MCP-1, eotaxin are increased and GROα, IFN-a2, TGFβ1 are reduced. Conversely, no sign of inflammation was reported by studies investigating ADHD. Possible correlation between changes in microbial taxa and disease severity can be extrapolated across the three disorders. ASD severity is positively correlated with *Desulfovibrio* (phylum Proteobacteria). Hyperactivity and impulsivity (ADHD symptoms) are negatively correlated with *Faecalibacterium* (phylum Firmicutes) and positively correlated with *Bacteroides* sp. (phylum Bacteroidetes) in ADHD. A reduced alpha-diversity and increased beta diversity are associated with severe RETT (compared to mild/moderate). CDT was increased in the ADHD bacteriome. CDT is responsible of producing phenylalanine, an essential AA that crosses the BBB and acts as a precursor for dopamine and noradrenaline, key neurotransmitters involved in modulating behavior-related neuronal pathways in the brain. AA, amino acid; ADI-RRB, autism diagnostic interview-restricted and repetitive behaviors; ADHD, attention deficit hyperactivity disorder; ASD, Autism Spectrum Disorder; BBB, blood brain barrier; BCFAs, branched chain fatty acids; CDT, cyclohexadienyl dehydratase; GABA, Gamma-aminobutyric acid; GI, gastrointestinal; GROα, growth-related oncogene alpha; IL, Interleukin; IFN, Interferon; MCP-1, monocyte chemoattractant protein; NT, neurotypical controls; RETT, Rett syndrome; SCFAs, short chain fatty acids.

Two studies investigated possible correlations between intestinal microbiota changes and ASD severity. Ahmed et al. (2020) assessed ASD subjects with mild–moderate (*n* = 35) and severe ASD (*n* = 6). No statistically significant differences were found across most taxa, except for Firmicutes taxa which were significantly higher in patients with severe ASD (Figure 2; Table 2). This was also described by Tomova et al. (2015), who found a lower Bacteroidetes to Firmicutes ratio in children with severe ASD, compared to children with milder ASD and NT controls, respectively (Supplementary Table S9). This study also found children with severe ASD had higher *Desulfovibrio* and *Clostridia* compared to children with a milder ASD phenotype (Tomova et al., 2015) (Supplementary Table S9). A strong correlation between *Desulfovibrio* and the ADI restricted/repetitive behavior subscale score (a measure of ASD severity) was reported by Tomova et al. (2015) (Figure 2; Supplementary Table S9). These studies suggest a possible association between the phylum Firmicutes and ASD severity.

### Changes in intestinal mycobiota composition in ASD

Three studies investigated alterations in fungi composition of stool samples from ASD subjects (Table 1). Strati et al. (2016) observed a significant difference in the composition of the gut mycobiome between subjects with ASD and unrelated NT controls with enrichment in relative abundance of the genus, *Candida* in ASD. The study by Kantarcioglu et al. (2016) also reported increased levels of *Candida* species in stool specimens from ASD subjects, and Hughes and Ashwood (2018) observed an increased concentration of *anti-C. albicans* immunoglobulin (IgG) in serum samples (Hughes and Ashwood, 2018; Figure 2). Further studies are required to assess the role of fungi in ASD.

## Characteristics of the intestinal microbiota in ADHD

### Changes in bacterial diversity in ADHD

Five studies did not find any significant differences in bacterial α- or β-diversities indices between ADHD subjects and NT controls, including those studies where the age of the subjects was >18 years (Aarts et al., 2017; Zhai et al., 2019; Szopinska-Tokov et al., 2020; Wan et al., 2020; Richarte et al., 2021; Szopinska-Tokov et al., 2021; Table 3). Prehn-Kristensen et al. (2018) observed significantly decreased richness and evenness in the gut microbiota of ADHD subjects and their mothers compared to unrelated NT controls. The same study observed significant differences in bacterial profiles between ADHD subjects and NT controls, and between ADHD subjects and their mothers (Prehn-Kristensen et al., 2018; Table 3). Wang et al. (2020) found changes in bacterial α-diversity in ADHD subjects and NT controls, with no significant differences in bacterial composition overall (Table 3). Only two studies evaluated first-degree NT siblings of ADHD subjects, however NT siblings were mixed with unrelated NT controls and data were analyzed as a single group (Aarts et al., 2017; Szopinska-Tokov et al., 2021). Diversity measures showed no differences in bacterial communities between ADHD subjects and NT controls. Changes in α-diversity observed in the studies by Prehn-Kristensen et al. (2018) and Wang et al. (2020) need further validation using larger cohorts.

### Changes in the relative abundance of bacterial taxa in ADHD

The principal phyla characterizing the bacterial kingdom of the human microbiota, Firmicutes, Bacteroidetes, Actinobacteria, and Proteobacteria, were identified across all ADHD studies. One study found a significant increase of Actinobacteria, accompanied by a decrease of Firmicutes in ADHD gut microbiota (Aarts et al., 2017). Further taxonomic analyses showed that taxa from families and genera belonging to the phyla Firmicutes and Bacteroidetes were primarily associated with ADHD diagnosis (Figure 2; Table 3). At least two studies reported reduced abundance of bacteria from the family *Oscillospiraceae* (Aarts et al., 2017; Wan et al., 2020) (renamed from *Ruminococcaceae* (Tindall, 2019)), and the genera *Faecalibacterium* (Jiang et al., 2018; Wan et al., 2020) and *Coprococcus* (e.g., *Coprococcus_2*) (Aarts et al., 2017; Szopinska-Tokov et al., 2021) within the ADHD gut microbiota (Table 3). Within the phylum Bacteroidetes, the abundance of bacteria from the genera *Bacteroides*, *Alistipes*, *Odoribacter*, and *Parabacteroides*, as well as from the family *Bacteroidaceae*, were reported to be increased in ADHD subjects (Table 3). Increased abundance of *Egghertella*, *Neisseria* (phylum Proteobacteria), *Bifidobacterium* (phylum Actinobacteria), and *Fusobacterium* (phylum Fusobacteria) were also observed in the gut microbiota of ADHD subjects. At the species level, two studies reported increased amounts of *Bacteroides uniformis* and *Bacteroides ovatus* in ADHD gut microbiota (Aarts et al., 2017; Wang et al., 2020). Increased relative abundance of *Bacteroides vulgatus*, *Bacteroides caccae*, *Paraprevotella xylaniphila*, *Odoribacter splanchnicus* (phylum Bacteroidetes) as well as *Bifidobacterium longum*, *Bifidobacterium adolescentis* (phylum Actinobacteria), and *Sutterella stercoricanis* (phylum Proteobacteria) were observed in ADHD-associated microbiota (Table 3). Each set of findings were reported in only one study; further validation across larger samples is needed.

### Changes in bacterial functional profile in ADHD

Changes in inferred metabolic function of the intestinal microbiota in ADHD subjects were assessed by two studies using PICRUSt-KEGG analysis. Wan et al. (2020) found an enrichment in the enzymatic pathways involved in dopamine turnover at the postsynaptic neuron as well as in genes encoding the catalytic subunit of protein phosphatase-1 (PP1), threonine synthase, and 6-pyruvoyl-5,6,7,8-tetrahydropterin, and depletion of the gene encoding 4-hydroxy threonine-4-phosphate dehydrogenase in ADHD-associated microbiota (Wan et al., 2020; Table 3). PP1 catalytic subunit has been described to increase synaptic sodium ion flux and may impact transmembrane sodium/chloride-dependent transporter dopamine receptors (Navratna and Gouaux, 2019). Abnormal *Enterococcus* and *Odoribacter* were found to be associated with alterations in the dopamine metabolic pathway in ADHD patients compared to NT controls (Wan et al., 2020). Aarts et al. (2017) reported increased abundance of the enzyme cyclohexadienyl dehydratase (CDT) in the gut microbiota of ADHD subjects. CDT is responsible for the production of phenylalanine, a precursor for the synthesis of dopamine and noradrenaline. Predicted CDT was found to be positively correlated with *Bifidobacterium* in ADHD. Reward anticipation, a key symptom in ADHD mediated by dopamine, was negatively correlated with predicted CDT in ADHD subjects (Aarts et al., 2017; Table 3). This study suggests that *Bifidobacterium* may enhance production of phenylalanine via the enzyme CDT in patients with ADHD. Several studies have assessed the role of phenylalanine in ADHD with conflicting results, showing decreased concentrations of phenylalanine in ADHD and no association (Bornstein et al., 1990; Baker et al., 1991; Bergwerff et al., 2016). In contrast, among patients with the metabolic condition, phenylketonuria, toxic phenylalanine build-up may lead to ADHD symptoms (Stevenson and McNaughton, 2013; Ashe et al., 2019; Beckhauser et al., 2020).

### Correlation between bacterial profiles and disease severity

None of the studies in our review stratified the microbiota data according to the ADHD severity. Some correlations between bacterial taxa and ADHD symptoms, however, were reported. The relative abundance of *B. uniformis*, *B. ovatus*, and *S. stercoricanis* correlated with ADHD symptoms (Wang et al., 2020), and *Coproccocus_2* with inattention symptoms, even though this association was not statistically significant (*p* = 0.055) (Szopinska-Tokov et al., 2020) (Figure 2; Table 3). A negative correlation occurred between hyperactivity scores and α-diversity in ADHD patients (Prehn-Kristensen et al., 2018), and between hyperactivity and impulsivity and *Faecalibacterium* levels (Figure 2; Table 3). No correlations were identified between microbiota changes and attention deficit and impulsivity (parental rating assessed by the German ADHD Rating Scale, FBB-HKS), or clinical symptoms assessed by the CBCL. A positive correlation between *Bacteroides* and levels of hyperactivity and impulsivity was observed (Figure 2; Table 3). The authors further assessed intestinal microbiota of mothers of ADHD patients and found patterns of similarly reduced α-diversity, which correlated with maternal ADHD symptoms as compared to NT maternal controls. There were no differences between α-diversity and ADHD symptoms between fathers of ADHD patients and fathers of NT controls (Prehn-Kristensen et al., 2018). The study of Richarte et al. (2021) did not find any correlation between bacterial taxa (family, genus) and ADHD rating scale scores; however, some correlations between bacterial taxa among ADHD subjects were identified (Table 3). Together these findings suggest relationships between ADHD symptoms and *Bacteroides*, yet the biological mechanisms behind these remain unknown.

## Characteristics of the intestinal microbiota in Rett syndrome

### Changes in bacterial diversity in Rett syndrome

Two studies from Italy showed a reduction in α- and β-diversity between RETT subjects and NT controls (Strati et al., 2016; Borghi et al., 2017). The study by Borghi et al. (2017) did not reach statistical significance. The authors suggested that this result was affected by the small sample size (Table 4). A third study, conducted in United States, did not find any differences in α- and β-diversities between RETT and NT controls; however, changes in bacterial richness, diversity, and composition were observed within RETT subjects based on pubertal stage, severity of disease and dietary habits (Thapa et al., 2021) (Table 4). These studies indicate a possible change in bacterial diversity associated with RETT. However further analyses in larger cohorts are required to confirm these results.

### Changes in the relative abundance of bacterial taxa in Rett syndrome

Most changes in the abundance of bacterial taxa associated with RETT occurred within the Firmicutes and Bacteroidetes phyla, followed by Actinobacteria and Proteobacteria phyla (Figure 2; Table 4). In general, RETT microbiota was characterized by reduced abundance of bacterial genera from the family *Oscillospiraceae* (*Ruminococcus*, *Faecalibacterium*, *Oscillibacter*, *Sporobacter*) and several genera from the phylum Bacteroidetes (*Prevotella*, *Barnesiella*, *Alistipes*, *Odoribacter*, *Butyricimonas*).

Conflicting findings were reported for the genus *Bacteroides* (Strati et al., 2016; Borghi et al., 2017). An increased relative abundance of the genera *Lactobacillus, Enterococcus* (phylum Firmicutes), *Egghertella*, *Bifidobacterium*, *Actinomyces* (phylum Actinobacteria) were observed in the gut microbiota of RETT subjects. Strati et al. reported decreased abundance of bacteria from the genus *Bilophila* and increased levels of bacteria in genera *Escherichia* and *Shigella* within phylum Proteobacteria (Strati et al., 2016). At the species level, subjects with RETT in the study by Borghi et al. (2017) showed decreased abundance of *F. prausnitzii*, *Roseburia* sp.*, Prevotella* sp. and an increased abundance of *Clostridium* sp., and *Sutterella* sp., compared to NT controls. The study by Strati et al. (2016) also reported increase abundance of *Bifidobacterium longum* in RETT-associated microbiota (Table 4). Overall, these results suggest the presence of bacterial changes across multiple bacterial phyla that are associated with RETT.

### Changes in bacterial functional profile in Rett syndrome

Two studies investigated the potential metabolic function of gut bacteria using PICRUSt-KEGG analysis. Strati et al. (2016) observed an enrichment in carbohydrate and propanoate metabolism (pathways of SCFA production) in the gut microbiota of RETT subjects. In the study by Borghi et al. (2017), enzymes for carbohydrate and lipid metabolism were depleted, whereas amino acids pathways, butanoate and propanoate metabolism were enriched in RETT subjects (Table 4). These data suggest that the bacterial metabolic pathways responsible for SCFA production are altered in RETT. As these findings were only derived from two studies, larger cohorts of patients are required for further validation.

### Correlation between bacterial profiles and disease severity

Specific correlations between intestinal microbial dysbiosis and disease severity in RETT were only assessed by Thapa et al. (2021), who found a significant reduction in α-diversity and differences in β-diversity between subjects with severe RETT compared to mild and moderate phenotypes (Figure 2; Table 4). Further studies are needed to understand whether there is a link between the composition of bacterial communities and RETT severity.

### Changes in gut mycobiota composition in Rett syndrome

One study investigated alterations in the gut mycobiota associated with RETT (Strati et al., 2016). Fungal β-diversity measurements showed a significant difference between RETT subjects and NT controls (Table 4). Metataxonomic analysis led to the identification of 77 fungal genera that differed between groups, including *Candida, Penicillium, Aspergillus, Malassezia*. Among identified genera, only *Candida* was increased in RETT subjects compared with NT controls (Figure 2; Table 4).

## Changes in host and microbial-derived metabolites associated with ASD, ADHD, and Rett syndrome

### ASD

Several studies assessed the concentration of microbial-derived metabolites in stool samples from ASD subjects and evaluated possible correlations with changes in microbial taxa associated with ASD. De Angelis et al. (2013) found that the metabolism of free amino acids (FAA) differed between patients with PDD-NOS and ASD compared to NT first-degree sibling controls. Levels of FAA and volatile organic compounds (VOCs) of subjects with PDD-NOS were more similar to NT controls than those of ASD on principal component analysis (PCA) plots (De Angelis et al., 2013).

Glutamine was highest in fecal samples of subjects with ASD compared to NT controls while indole, a microbial metabolite of tryptophan (which converts to serotonin) and 3-methylindole were increased in ASD and PDD-NOS subjects (De Angelis et al., 2013; Table 2). Kang et al. (2018) found trimethylamine, malonate, methylamine, choline, and 3- phenylpropionate weakly correlated with age in ASD subjects but were not statistically significant after correction for multiple hypothesis testing. Fecal concentrations of GABA, a major inhibitory neurotransmitter, were lower in stool of children with ASD in this cohort, although differences were only marginally significant. Concentrations of glutamate, a major excitatory neurotransmitter, were comparable between groups. GABA to glutamate ratio was observed to be marginally lower in stool of ASD subjects (Kang et al., 2018; Table 1).

Phenol compounds (phenol, 4-(1,1- dimethylethyl)-phenol, and para-Cresol) and methyl esters were increased in the stool of both PDD-NOS and ASD subjects compared to NT controls (De Angelis et al., 2013). This was also reported by Kang et al. (2018), who found significantly higher concentrations of p-cresol in stool of children with ASD compared to neurotypical controls. p-Cresol is introduced to the body either through skin, inhalation, or microbially produced in the intestine from dietary tyrosine or toluene substrates. The authors found no significant correlation between p-cresol and tyrosine in subjects’ stool (Spearman correlation, r = 0.08, *p* = 0.62) (Kang et al., 2018). p-cresol concentrations were also found to be significantly and negatively correlated with age within the ASD group (Spearman correlation, *r* = 0.47, *p* = 0.02), whereas no significant correlation was observed within NT subjects (Spearman correlation, *r* = 0.22, *p* = 0.34) (Kang et al., 2018) (Supplementary Table S5).

The study of Kang et al. (2018) involved multiple testing corrections (permutation-based non-parametric significance test, adjusted *p* = 0.022) among 59 metabolites and found that isopropanol was the only metabolite that was significantly different between ASD and NT controls after multiple testing corrections. In this same study, metabolite combinations from the 25 most differently abundant metabolites were evaluated to determine if they could support differentiation of ASD subjects from NT controls. Only five out of 23 children with ASD (78% sensitivity), and four out of 21 neurotypical controls children would not be classified as belonging to their true group (81% specificity). Further, using a more stringent threshold (>30% taxa membership in ASD subjects), cross-validation showed 70% sensitivity and 95% specificity (Kang et al., 2018). This analysis showed higher concentrations of caprate and aspartate in stool of subjects with ASD, while nicotinate, glutamine, and thymine were lower.

Differences in SCFA have also been described between subjects with ASD and NT controls (Figure 2; Tables 1, 2) Children with ASD showed significantly lower levels of total SCFA compared to NT, except for propionic acid which was increased (De Angelis et al., 2013). The proportion of SCFA correlated with levels of *Faecalibacterium, Ruminococcus* and *Bifidobacterium* (De Angelis et al., 2013). Liu et al. (2019) found correlations between microbial taxa and decreased fecal SCFA in ASD. *Ruminococcaceae*, *Peptostreptococcaceae, Lactobacillales, Streptococcaceae*, *Eubacterium* and *Lachnospiraceae-NC2004* all correlated with decreased fecal concentrations of butyrate. A correlation was found between relative Acidobacteria levels and increased levels of fecal valerate (Spearman correlation, rs = 0.349, *p* = 0.013), *Desulfovibrionaceae* and increased fecal acetate (*r* = 0.360, *p* = 0.01), and *Streptococcaceae* and increased propionate (*r* = 0.368, *p* = 0.009) (Liu et al., 2019). Subjects in both cohorts were thought to have similar dietary contributions of butyrate and valerate, suggesting that differences in microbial taxa were the primary determinant of SCFA changes (Liu et al., 2019). Kang et al. (2018) found that fecal concentrations of propionate and butyrate were not different between ASD and NT controls. Other carbohydrate fermentation products of intestinal bacteria, formate and lactate, were detected at relatively lower concentrations in children with ASD, but their differences were not significant after multiple testing corrections (Table 1).

The study by Zhai et al. (2019) described high levels of Pb, As, Cu, Zn, Mg, Ca and Hg in children with ASD (Figure 2; Table 1). In particular, As and Hg were positively correlated with *Oscillospira* and *Parabacteroides* in ASD. Luna et al. (2017) assessed tryptophan and serotonin metabolite concentrations in supernatants of rectal biopsies obtain from ASD patients (all displaying FGID) and correlated these data with bacterial taxa that were abundant in biopsy preparations from an ASD cohort. Decreased tryptophan and increased 5-hydroxyindoleacetic acid (5-HIAA) levels were found in ASD rectal biopsies compared to NT control rectal biopsy preparations (Luna et al., 2017). *Erysipelotrichaceae*, *C lituseburense*, and *Terrisporobacte*r species correlated with tryptophan concentrations from the rectal biopsies of the ASD cohort. *L. bolteae*, *L. hathewayi*, and *F. plautii* correlated with levels of serotonin in ASD tissue biopsy specimens (Luna et al., 2017; Table 1; Supplementary Table S5).

### ADHD

Microbial-derived metabolites were not directly investigated in ADHD studies.

### Rett syndrome

Among RETT subjects, Borghi et al. (2017) found fecal butyrate and propionate to be significantly increased, as well as iso-butyrate and iso-valerate. This was found in conjunction with a greater abundance of Bacteroidetes and *Veillonaceae*, major propionate producers (Borghi et al., 2017; Table 4). Similar findings were described in a study by Strati et al. (2016), who found that the fecal content of SCFAs in RETT subjects was significantly enriched in propionate, isobutyrate and 2- methylbutyl isovalerate (Table 4). Concentrations of selected protein end-products of the gut bacterial metabolome, including GABA, tyrosine, and glutamate, were reduced in RETT subjects as compared to NT (Thapa et al., 2021; Table 4).

Overall, these studies show changes in microbial-host metabolites, neurotransmitters and trace elements in stool and intestinal mucosa of ASD and RETT patients that correlated with bacterial taxa altered in ASD patients. These results require further validation to infer a causal relationship between microbiota changes and neurodevelopmental outcomes.

## Changes in inflammatory markers associated with ASD, ADHD, and Rett syndrome

### ASD

Few studies assessed correlations between immune dysregulation and changes in microbial taxa associated with ASD (three studies) or RETT (one study). No studies assessed these relationships in individuals with ADHD. Hughes and Ashwood (2018) reported that children with ASD have elevations in IgG against *Candida albicans* with concomitant elevations of interleukin (IL)-17 (Table 1). Rose et al. (2018) also found that peripheral blood mononuclear cells (PBMC), isolated from the blood of subjects with ASD and GI symptoms (ASD-GI), produced elevated levels of interleukin (IL)-17, as well as IL-5 and IL-15, after exposure to the toll-like receptor (TLR)-4 agonist lipopolysaccharide (LPS), a component of gram-negative bacteria cellular walls. This elevation specifically occurred in PBMC isolated from ASD-GI subjects, while those with ASD-no-GI showed increased levels of IL-1α, IL-1β and Tumor Necrosis Factor (TNF)-α after TLR-4 stimulation of PBMC with LPS (Rose et al., 2018). Moreover, production of Transforming Growth Factor (TGF)-β1 was consistently decreased in ASD-GI subjects, as compared to subjects with ASD-no-GI and NT controls. The study by Luna et al. (2017) measured cytokines levels in the blood and biopsy supernatants from ASD-GI and NT controls, with or without GI symptoms (Figure 2; Table 1). In serum, decreased growth related oncogene alpha (GROα) and Interferon (IFN)-a2, and increased monocyte chemoattractant protein (MCP) and eotaxin levels were observed in ASD-GI subjects compared with NT-GI and NT controls. Moreover, increases in MCP-1 and eotaxin were found in ASD-GI subjects with abdominal pain. Luna et al. (2017) reported positive correlations between serum/mucosal cytokines and bacterial species associated with ASD-GI group (Table 1) and also between cytokines and bacterial species associated with pain in the ASD-GI group. *C. disporicum* correlated with macrophage inflammatory protein 1 alpha (MIP-1a) and beta (MIP-1β), vascular endothelial growth factor (VEGF), IFN-γ, IL12p70, IL17A, IL5, IL6 and IP-10. *C. tertium* correlated with IL1RA, IFN-γ, IL12p70, IL17A, IL1a, IL5, IL6, MIP-1a, and MIP-1b; *P. excrementihominis* correlated with GROα, and *Tyzzerella nexilis* with soluble CD40 ligand (Luna et al., 2017). The study by Tomova et al. (2015), which assessed probiotic supplementation in ASD subjects, identified a significant decrease in relative abundance of Firmicutes, *Bifidobacterium* and *Desulfovibrio* that correlated with decreased fecal TNFα levels in probiotic-treated ASD subjects (Table 5; Supplementary Table S9). Oxytocin was significantly lower in the plasma of subjects with ASD and NT first-degree sibling controls compared to unrelated NT controls. A positive correlation was found between plasma oxytocin levels and ASD severity (ADI) (*r* = 0.71, *p* < 0.05) (Tomova et al., 2015). Plasma levels of dehydroepiandrosterone sulfate (DHEA-S) were significantly lower in children with ASD, compared to NT first-degree sibling controls or unrelated NT controls. Testosterone levels did not show significant differences between groups but were positively correlated with ASD severity (as measured by ADI scores) (Tomova et al., 2015; Supplementary Table S9).

### ADHD

Inflammatory markers were not investigated in ADHD studies.

### Rett syndrome

Strati et al. (2016) found that subjects with RETT had elevated levels of fecal calprotectin and serum erythrocyte sedimentation rate (ESR), which correlated with levels of serum IgA antibodies (Table 4). These increased inflammatory markers were hypothesized to be associated with RETT subjects’ lower intestinal bacterial microbiota richness and diversity as compared to NT controls (Strati et al., 2016).

## Gastrointestinal comorbidities associated with ASD, ADHD, and Rett syndrome

### ASD

GI symptoms were assessed using the Questionnaire on Pediatric Gastrointestinal Symptoms-Rome III (QPGS-RIII) in five studies (Son et al., 2015; Luna et al., 2017; Strati et al., 2017; Hughes and Ashwood, 2018; Rose et al., 2018) and using the 6-item Gastrointestinal Severity Index (6-GSI) in four studies (Kang et al., 2013, 2018; Liu et al., 2019; Ahmed et al., 2020; Supplementary Tables S5, S6). In two studies, GI symptoms were evaluated based on parental questionnaires (Gondalia et al., 2012; Tomova et al., 2015; Supplementary Tables S5, S6), and four studies did not assess the presence of GI symptoms in their patient cohorts (De Angelis et al., 2013; Pulikkan et al., 2018; Zhang et al., 2018; Zhai et al., 2019). Many ASD subjects included in these studies manifested GI symptoms including constipation, diarrhea, abdominal pain, and others.

Liu et al. (2019) observed that only constipation was significantly increased in ASD subjects compared to NT controls. ASD subjects with constipation displayed stool enrichments in *Fusobacterium*, *Barnesiella, Coprobacter* and valeric acid-associated taxa (*Actinomycetaceae*), and reduced butyrate-producing taxa (Liu et al., 2019). The study by Strati et al. (2017) found that taxa belonging to *Clostridium cluster XVIII* and pro-inflammatory bacteria *Escherichia/Shigella* positively correlated with ASD constipation status. Finegold et al. (2017) did not report their methodology for assessment of GI symptoms. This study found that samples from subjects with ASD have significantly higher counts of β2-toxin expressing-*Clostridium perfringens* compared to NT controls. 79% of the 33 ASD subjects showed the presence of β2- toxin gene in stool, compared to 38% of the 13 NT controls. Those subjects with ASD also showed more severe GI symptoms, although the correlation between β2- toxin gene levels and GI symptoms severity was not assessed. Bacterial richness was negatively correlated with GI symptoms in ASD subjects in the study by Kang et al. (2013) (Table 1). Almost 40% of anti-*C. albicans*^+^ IgG-or anti-*C. albicans*^-I^ IgG- ASD subjects had GI symptoms in the study by Hughes and Ashwood (2018). Son et al. (2015) observed increased abdominal pain and functional constipation in ASD subjects compared with their NT siblings, but no significant correlation was identified between ASD, FGIDs or gut bacteria composition (Son et al., 2015).

In a study evaluating 50 individuals with ASD, Rose et al. (2018) showed that the relative abundance of *Bacteriodaceae, Lachnospiraceae, Ruminococcaceae* and *Prevocellaceae* differed between ASD-GI subjects and NT-GI controls. No differences were found among these taxa between ASD-no-GI subjects, and NT-no-GI controls. ASD-GI subjects had more irritability and agitation, social withdrawal, lethargy, and hyperactivity compared to ASD-no-GI. Further analyses showed that microbiota-related biological pathways were differentially represented between groups and that alterations were related to the presence of ASD, and not GI symptoms independently (Rose et al., 2018). In contrast, the studies by Tomova et al. (2015) and Kang et al. (2018) reported positive correlations between GI symptoms and ASD severity in their cohort of patients. Luna et al. (2017) found positive correlations between bacterial taxa (from mucosal biopsies) and GI symptoms in both ASD and NT subjects. Six bacterial taxa were higher in ASD with abdominal pain compared to ASD without abdominal pain, as well as NT controls with and without abdominal pain (Luna et al., 2017; Table 1).

Ahmed et al. (2020) showed that the 6-GSI score was not correlated with changes in microbiota profiles among subjects with ASD. This was corroborated by Gondalia et al. (2012), who also found no differences in intestinal microbial profiles between ASD-GI and ASD-no-GI subjects.

In the study by Yap et al. (2021), reduced stool consistency, as measured on one stool sample using the Bristol Stool Chart, was associated with reduced dietary and microbiota diversity in ASD patients. Kushak et al. (2017) reported constipation and gastroesophageal reflux in ASD subjects assessed for GI symptoms through upper GI endoscopy. However, the authors did not observe any effects of GI symptoms on mucosal-associated microbiota or duodenal enzyme activity (Kushak et al., 2017). Overall, these findings confirm the presence of GI comorbidities in subjects with ASD. Nevertheless, relationships between gut microbes, GI symptoms and ASD-related neurobehavioral outcomes remain unclear.

### ADHD

The study by Wan et al. (2020) was the only study included in our systematic review to describe GI comorbidities in ADHD patients. An association was reported between bacterial changes and constipation symptoms in the ADHD cohort (Supplementary Table S7).

### Rett syndrome

All individuals with RETT suffered from constipation, although relationships between microbiota alterations or disease severity with GI symptoms were not investigated (Table 4; Supplementary Table S8).

## Effect of dietary habits on the intestinal microbiota in subjects with ASD, ADHD, and Rett syndrome

### ASD

Eight out of 17 studies on ASD documented dietary patterns of patients. The effect of diet in shaping the gut microbiota composition, however, was evaluated only by Yap et al. (2021) (Table 2; Supplementary Table S6). The investigators in this study performed stool metagenomics in a large (*n* = 247) cohort of ASD and NT controls from a closely observed population from the Australian Autism Biobank and Queensland Twin Adolescent Brain project. ASD subjects had greater restriction in food intake and a less-diverse diet, which was associated with lower microbial α-diversity (Yap et al., 2021). This study found little evidence for a direct association between intestinal microbiota and ASD; rather, any observed changes were found to be most associated with influences of stereotypic dietary intakes in ASD subjects compared to NT controls (Yap et al., 2021).

### ADHD

A strictly controlled cross-sectional cohort study was conducted by Prehn-Kristensen et al. (2018) where subjects with ADHD were matched with NT controls with similar BMI and food intake (meat, fruits/vegetables, yogurt, milk products, or fast food during the month prior to microbiota analysis; Table 3; Supplementary Table S7). Differences were observed in α-diversity between subjects with ADHD and NT, and authors speculated that microbial changes in ADHD individuals were due to the underlying disease and not dietary patterns. In a cohort study by Wang et al. (2020), diets of ADHD subjects and NT controls were compared. ADHD subjects were found to have higher intake of refined grains (*p* = 0.027) and a lower proportion of dairy (*p* = 0.020) and vitamin B2 (*p* = 0.033) intake (Wang et al., 2020). In this study, *S. stercoricanis* was significantly associated with dairy, nut, seed and legume intake, and *B. uniformis* correlated with fat and carbohydrate intake. *S. stercoricanis* and *B. ovatus* also positively correlated with ADHD symptoms. These findings correlate with findings from Aarts et al. (2017), who demonstrated greater abundance of *B. uniformis* and *B. ovatus* in ADHD patients. *Bacteroides* sp. have been associated with the development of the frontal lobe, cerebellum and hippocampus (Aarts et al., 2017; Table 3; Supplementary Table S7). Overall, these data suggest that changes in the microbiota may correlate with dietary intake and not the actual disease state.

### Rett syndrome

The influence of diet on gut microbiota profiles among NT controls and RETT subjects was only investigated by Thapa et al. (2021) (Table 4; Supplementary Table S8). Differences in bacterial composition and increased α-diversity were observed in RETT subjects who consumed table foods alone or combined with formula compared with RETT subjects who received exclusive formula. However, the effect of the diet in influencing disease severity was not investigated (Thapa et al., 2021).

## Effects of microbiota- or diet- targeted interventions on intestinal dysbiosis and behavioral outcomes in subjects with ASD, ADHD, and Rett syndrome

### Probiotic treatments

Two studies examined the effects of probiotic treatment on gut microbiota profiles, disease outcomes and GI symptoms in patients with ASD (Tomova et al., 2015; Shaaban et al., 2018; Table 5; Supplementary Table S9). Both studies used a mix of *Lactobacillus* and *Bifidobacteria* strains, orally administered once (Shaaban et al., 2018) or three times per day (Tomova et al., 2015) for one month (Table 5). In both studies, NT controls were siblings of ASD subjects. Shaaban et al. (2018) found that decreases in the amount of *Bifidobacterium* in stool from ASD subjects recovered to levels observed in NT controls following probiotic treatment (Table 5). Conversely, Tomova et al. (2015) showed higher amounts of *Bifidobacterium* and increased *Clostridium, Desulfovibrio*, and *Lactobacillus* in ASD subjects. After probiotic treatment, levels of *Bifidobacterium* and *Lactobacillus* of ASD subjects decreased, reaching levels observed in NT controls (Tomova et al., 2015).

Tomova et al. (2015) did not assess the effect of probiotic treatment on disease severity. Shaaban et al. (2018) observed a significant decrease in ASD severity in probiotic-treated patients, represented by improvements in ATEC scores (Table 5).

In the study by Pärtty et al. (2015), *Lactobacillus rhamnosus GG* was administered to mothers daily, from four weeks before delivery up to six months of life (Table 5). Subjects were followed for 13 years to assess whether probiotic administration in early life reduced the risk of developing AS and ADHD in adolescence (Pärtty et al., 2015). ADHD or AS was diagnosed in 17.1% of subjects in the placebo group vs. 0% of the probiotic-supplemented group by age 13. There were no differences in bacterial abundance between ADHD subjects and NT controls, by 3 months or 13 years. Decreases in *Bifidobacterium*, *Clostridium histolyticum*, *Bacteroides*, *Lactobacillus*, and *Enterococcus* observed in 2-year-old children treated with placebo were associated with the development of AS or ADHD at 13 years old. These taxa differences did not persist at age 13 years (Pärtty et al., 2015; Table 5).

### Fecal microbiota transplantation

In an open-label study, Kang et al. (2017) showed that fecal microbiota transfer (FMT) to patients with ASD, using stool derived from healthy controls (Standardized Human Gut Microbiota) (Hamilton et al., 2012) (Table 5; Supplementary Table S9) increased bacterial diversity compared to NT controls and remained higher than baseline bacterial diversity 8 weeks after final administration of FMT (Kang et al., 2017). In ASD patients, the relative abundance of *Bifidobacterium*, *Desulfovibrio*, and *Prevotella* increased following FMT. Median microbial richness at week 18 was not statistically different between ASD and NT control groups (*p* > 0.05). A positive engraftment of phageome from the healthy donor to ASD subjects and increased abundance of phage was also observed after FMT (Table 5). Similar to the probiotic treatment described by Shaaban et al., FMT significantly improved ASD symptoms by improving social skill deficits, stereotypy, communication, daily living skills and socialization (Kang et al., 2017) (Table 5).

Probiotic and FMT intervention have demonstrated beneficial effects in improving GI symptoms in ASD patients (Table 5). Among recipients of probiotic supplementation, improvements in GI symptoms (assessed by the 6-GSI) were also strongly correlated with improvements in autism severity (assessed by the ATEC) (*r* = 0.674, *p* = 0.0001; Shaaban et al., 2018). Specifically, these subjects had a significant reduction in constipation scores (*p* < 0.01), stool consistency (*p* < 0.023), flatulence (*p* < 0.037) and abdominal pain (*p* < 0.002). Further, a significant correlation was found between probiotic administration, stool colony counts of *Bifidobacteria*, and reduction of constipation severity score (*r* = −0.441, *p* < 0.015; Shaaban et al., 2018). In the FMT study by Kang et al. (2017), 89% of ASD subjects had improvement in GI symptoms as reflected by an 80% reduction in the Gastrointestinal Symptom Rating Scale (GSRS). Further, improvements persisted up to 8 weeks after FMT (follow up) though similar changes in microbial richness were also observed in one of two non-responders (subjects whose GI symptoms improved less than 50% on the GSRS; Kang et al., 2017).

### Dietary interventions

Stevens et al. (2019) assessed the effects of dietary supplementation on the modulation of ADHD symptom expression and gut microbiota composition in a subset of participants from a large RCT (Table 5; Supplementary Table S9). Participants ages seven to 12 years with a diagnosis of ADHD were randomized to receive a 10-week course of broad-spectrum micronutrients containing a mixture of vitamins, minerals, amino acids, and antioxidants, or inert placebo control. Subjects receiving the micronutrient supplementation had improved symptoms of attention, emotional regulation, and aggression relative to placebo. Eighteen children from this cohort (*n* = 8 placebo, *n* = 10 micronutrient group) had their fecal microbiota assessed and showed a decrease in *Bifidobacterium* genera by 25% in the micronutrient treatment group while Coriobacteriales increased though these changes were not significant (Stevens et al., 2019).

## Discussion

The gut microbiota contributes to the maintenance of human health by modulating the function of numerous organs (Al Nabhani and Eberl, 2020; Beckhauser et al., 2020; Wang et al., 2021), including the central nervous system (Ratsika et al., 2023). Changes in gut microbial communities have been described in children and adults with neurodevelopmental disorders. These patients display altered dietary patterns and GI comorbidities, particularly constipation, visceral sensitivity, and intestinal inflammation. These factors collectively play a critical role in shaping gut microbiota composition, especially in early life. Given this, a key unresolved question is whether changes in the microbiota dynamics are part of the biomolecular alterations that cause neurodevelopmental disorders or are merely a consequence of gastrointestinal dysfunctions or food selectivity more commonly associated with these conditions.

We present the first systematic review to date assessing the influence of altered gut microbiota composition on the expression of three highly prevalent pediatric and adult neurodevelopmental disorders: ASD, ADHD and RETT. Herein, we highlight the implications of intestinal microbiota changes on diverse aspects of host–microbe interactions that may contribute to underlying neuropathology. Our inclusion criteria identified studies that explore intestinal microbiota dynamics in stool and intestinal mucosal biopsy samples of children and adults affected by ASD, ADHD, and RETT. Associations between microbial changes, disease-related behavioral and gastrointestinal outcomes were evaluated across 13 ASD studies, five ADHD studies, and one RETT study, per our predefined review criteria (Hill et al., 2021). Further, nine ASD and three RETT studies also sought to evaluate associations between gut microbiota composition and alterations in microbial-derived metabolites, trace elements, enzymes, and/or immune-inflammatory markers. Lastly, we assessed studies that sought to explore the role of microbial- or dietary-mediated interventions in restoring gut eubiosis and behavioral outcomes.

The main finding that emerged from our review is the disproportionate abundance of bacterial taxa from the phyla Firmicutes and Bacteroidetes in the microbiota of individuals affected by ASD, ADHD, and RETT. These taxa could possibly alter host-microbe homeostasis toward a pro-inflammatory phenotype, ultimately contributing to the neurological symptoms typical of these three diseases. The majority of ASD and RETT subjects are affected by GI symptoms, and RETT subjects have intestinal inflammation. ASD subjects display changes in microbiota-derived metabolites (SCFAs, neurotransmitters, FAA), inflammatory markers such as interleukins, interferons, and other proinflammatory and regulatory cytokines. Increased levels of As and Hg, trace elements with potential neurotoxic activity were also found in ASD subjects. Symptoms of ADHD, particularly hyperactivity and impulsivity were negatively correlated with *Faecalibacterium* (phylum Firmicutes) and positively correlated with *Bacteroides* sp. (phylum Bacteroidetes). Individuals with RETT are mostly constipated and have intestinal dysbiosis, associated with increased SCFAs and reduced GABA and glutamate levels. Increased abundances of *Candida* sp. were observed in ASD and RETT subjects. Our review shows that microbiota-targeted interventions with probiotics or FMT are capable of ameliorating or preventing ASD and ADHD symptoms. However, despite such evidence, many inconsistencies were identified across the included studies, primarily due to the small sample sizes, a wide range of subjects’ age, cross-sectional design with single time-point sampling, different sequencing techniques and other many confounding factors including, but not limited to, GI dysmotility, differences in diet and sex. Therefore, further research in studies with larger sample sizes and longitudinal design is necessary to determine the extent of the contribution of the gut microbiota to the neurobehavioral alterations of neurodevelopmental disorders.

At first, we assessed bacterial diversity between neurodivergent and NT subjects. Our systematic review identified changes in α- and β-diversity between subjects affected by ASD or RETT, compared to NT controls. However, these changes were inconsistent among selected studies. A significant discrepancy occurred in studies comparing the gut microbiota of ASD individuals with NT first-degree sibling controls or unrelated NT controls. Most studies in which NT controls were first-degree siblings of ASD subjects did not detect any differences in α- or β-diversity of gut bacterial populations (Gondalia et al., 2012; Son et al., 2015; Pulikkan et al., 2018) but one study, which examined the gut microbiota of subjects with ASD and PDD-NOS, found that both α- and β- diversities were higher in ASD subjects than their NT first-degree siblings (De Angelis et al., 2013). Three studies (Tomova et al., 2015; Ahmed et al., 2020; Yap et al., 2021) compared the gut microbiota of ASD subjects with both NT first-degree siblings and unrelated NT controls. In these studies, no differences in gut microbiota diversity were found between the three groups. Distinct bacterial changes were observed only between ASD and unrelated NT controls, except for *Bifidobacterium*, which differed [lower (Ahmed et al., 2020) and higher (Tomova et al., 2015)] compared to NT first-degree siblings. Other studies enrolling unrelated NT controls have found significant differences in bacterial diversity between NT and ASD-associated microbiota. The same results emerged in studies of RETT subjects, where only unrelated NT controls were used for microbiota comparison (Kang et al., 2013, 2018; Strati et al., 2016, 2017; Borghi et al., 2017; Zhang et al., 2018; Liu et al., 2019; Zhai et al., 2019; Thapa et al., 2021).

The most plausible explanation for these discrepancies is that first-degree siblings of neurodivergent children possess similar alterations in their intestinal microbiome secondary to shared environmental exposures that include dietary habits from shared cooking, living conditions, and host genetic factors (Orsmond and Seltzer, 2007; Wade et al., 2015). Further, a higher relative prevalence of first-degree siblings of ASD subjects have been shown to display some neurocognitive alterations even without a diagnosis of ASD (Wade et al., 2015), suggesting that some NT siblings may manifest a disease subtype, with a different host-microbe phenotype, compared to unrelated NT individuals (Son et al., 2015).

Our review showed no difference in α- or β-diversities of the gut microbiota between individuals with ADHD and NT first-degree siblings, or unrelated NT controls.

Monozygotic twin studies have shown significant heritability of ADHD (h2 = 0.77-0.82), yet up to 20% discordance in diagnosis remains (Sprich et al., 2000; Thapar et al., 2013). Only one study in our systematic review found differences in gut microbiota composition between ADHD subjects and unrelated NT controls. Interestingly, the mothers of those ADHD individuals displayed reduced bacterial α-diversity compared to their offspring. This suggests that the microbiota could be partially involved in the intergenerational transfer of an altered gut-brain phenotype responsible for the disease (Prehn-Kristensen et al., 2018), a finding that has also been described in mouse models of inflammatory bowel disease. However, these studies’ cross-sectional design precludes firm conclusions.

The sample sizes of most of the studies included in our systematic review were small (N = 10-50), and this might have contributed to some of the variability in microbiota measurements. We included two studies that assessed microbiota changes in ASD (Yap et al., 2021) and ADHD (Richarte et al., 2021) in larger sample sizes (>100 individuals per group). Yap et al. (2021) performed shotgun metagenomics analyses in stool samples from a large cohort of ASD children (approximately 100 ASD subjects and unrelated NT controls, and 50 pairs of ASD subjects and NT first-degree siblings). This study found a significant association between microbiota changes in ASD and ASD-related dietary preferences, and no changes in the microbiota of ASD subjects as compared to NT controls (siblings, or unrelated healthy controls) (Yap et al., 2021).

Richarte et al. (2021) examined the gut microbiota of 100 medication-naïve ADHD subjects. Changes were found in relative abundance of several microbial taxa, but no differences were found in α- or β-diversity indices between ADHD and NT controls. This confirmed the findings of other studies with smaller sample sizes (Richarte et al., 2021). Conflicting results between the study by Yap et al. (2021) and other ASD studies might be secondary to differing methodologies used for microbial sequencing and bioinformatics analyses.

The choices of sequencing techniques, genome databases and sample sizes and other factors that may also shape the gut microbiota during early childhood development (e.g., GI dysfunction, dietary impact, and geographical locations) are crucial for determining the influence of microbial changes on neurobehavioral pathology (Hamady and Knight, 2009; Chavaria et al., 2022; Morton et al., 2022).

The two major bacterial phyla of the human gut microbiome, Firmicutes and Bacteroidetes, were evaluated across all studies. Our systematic review shows that differences in the relative abundance of both phyla, between ASD, RETT, and ADHD subjects and NT controls are not consistent, and the ratio between these two phyla does not seem to be an adequate differentiator between these neurodevelopmental disorders and NT control populations.

Taxa belonging to phylum Firmicutes are involved in the intestinal fermentation of carbohydrates undigestible by the host, which may result in the production of SCFAs, one of the most characterized families of microbial-derived metabolites that have also been recognized to influence brain function through different biological pathways within the MGBA (Plöger et al., 2012; Bourassa et al., 2016; Erny et al., 2021; O’Riordan et al., 2022). Increasing evidence suggests that SCFAs, such as butyrate, may be linked to neurodevelopment. Besides being an energy substrate for intestinal colonocytes and strengthening the intestinal epithelial barrier, butyrate participates in early life immune programming and modulates gene expression of brain neurotransmitters and structural components of the BBB (Zhang et al., 2018; Deleu et al., 2021; O’Riordan et al., 2022). Lower levels of butyrate or dysbiosis of butyrate-producing bacteria could be a potential contributor to the neuroimmune alterations observed in neurodevelopmental disorders such as ASD. Higher levels of propionate-producing bacteria have also been associated with the development of ASD in few studies (Finegold et al., 2002, 2010; Finegold, 2011). Propionate is a SCFA involved in gluconeogenesis and energy production in the liver, and studies in animals have shown that injection of propionic acid in the rat brain can induce neurobehavioral alterations characteristic of ASD (MacFabe et al., 2008; Abuaish et al., 2022). Our systematic review shows lower levels of butyrate and acetate, and increased levels of valeric acid and propionate in fecal samples from ASD individuals (De Angelis et al., 2013; Liu et al., 2019). These fluctuations of SCFAs levels are paralleled by reduced abundance of butyrate- and acetate-producing bacteria (Zhang et al., 2018) and increases in the proportion of *Desulfovibrio* (Finegold, 2011), *Clostridium* (Tomova et al., 2015) and *Bacteroides* (De Angelis et al., 2013), bacterial groups primarily involved in the production of propionate and acetate (Zhou et al., 2021; O’Riordan et al., 2022). Nevertheless, data on the effects of SCFA in ASD that emerged from our review warrant further exploration in larger sample sizes, as they do not correspond with what has been previously reported in literature (Adams et al., 2011; Wang et al., 2012),or show trends that are not statistically significant (Kang et al., 2018).

RETT is associated with high levels of propionate, isobutyrate, and isovalerate in the stool of affected subjects enrolled in the studies by Strati et al. (2016) and Borghi et al. (2017). The authors have linked the enrichment of *Bifidobacterium*, *Bacteroides, Clostridium, Enterococcus, Escherichia/Shigella* and *Lactobacillus* in RETT microbiota with increased production of SCFAs and have also demonstrated the role of SCFAs in slowing intestinal motility and causing constipation, a frequent GI comorbidity in RETT subjects (Strati et al., 2016; Borghi et al., 2017).

Distinct changes in bacterial taxa in subjects with ASD compared to NT controls were reported across multiple studies, with no specific taxa consistently identified. In ASD, decreases in *Faecalibacterium*, *Streptococcus* (Phylum Firmicutes), *Prevotella* (Phylum Bacteroidetes), *Bifidobacterium* (phylum Actinobacteria), and increases in *Clostridium*, *Lactobacillus* (phylum Firmicutes), and *Bacteroides* (phylum Bacteroidetes) were observed across studies that assessed bacterial taxonomic composition using the 16S rRNA gene (De Angelis et al., 2013; Kang et al., 2013, 2018; Strati et al., 2017; Pulikkan et al., 2018; Zhang et al., 2018; Liu et al., 2019; Zhai et al., 2019), by performing qPCR for specific bacterial taxa (Son et al., 2015; Tomova et al., 2015) or through quantification from bacterial cultures (De Angelis et al., 2013). Reduction in the genus *Prevotella* in children with ASD was one of the most consistent findings emerging from our systematic review, as well as from previous work (Ho et al., 2020). However, the role of this bacterial group in affecting host neurobiology is still unknown. In adults, a Western-style diet poor in fiber content and rich in saturated fat has been found to affect *Prevotella* and *Bacteroides* abundance (Derrien et al., 2019; Tett et al., 2021). As suggested by Yap et al. (2021), ASD is associated with reduced dietary diversity, which in turn can impact microbiome diversity and GI function. The altered dietary behaviors of ASD individuals have a significant impact on the developmental trajectory of the microbiome in early life, and a dysbiotic microbiome affects host physiology, metabolism, and brain function which may contribute to some of the behavioral traits seen in ASD.

ADHD and RETT are characterized by alterations in bacterial taxa belonging to the phyla Firmicutes and Bacteroidetes, and similar to ASD, these changes were not consistent across the included studies. A recent meta-analysis that analyzed some of the studies included in our systematic review identified a significant increase of *Blautia* (phylum Firmicutes) in ADHD subjects (Wang et al., 2022). This bacterial genus has been associated with various human diseases, such as depression, obesity, and diabetes (Wang et al., 2022), yet the link between *Blautia* and neurodevelopment is still undefined. Correlations between bacterial groups and disease symptoms, or bacteria-associated functional pathways related to dopamine or norepinephrine turnover have been described across different ADHD studies (Aarts et al., 2017; Jiang et al., 2018; Prehn-Kristensen et al., 2018; Wang et al., 2020).

Besides changes in microbial composition, our systematic review also identified other elements that link the microbiota with host neuronal function. For example, decreased GABA levels were observed in stool from ASD (De Angelis et al., 2013) and RETT (Thapa et al., 2021) subjects. Decreased tryptophan and increased 5-HIAA, the primary metabolite of serotonin, were observed in ASD intestinal tissues, and such changes were correlated with specific bacterial taxa. GABA, glutamine, serotonin, and tryptophan are neurotransmitters of the PNS and CNS that play a critical role in modulating neuronal activity within the gut-brain axis. Bacteria in the gut can synthesize and metabolize such neurotransmitters, using them as host-microbe interkingdom signaling molecules and thus contributing to the neuro-hormonal functions of the host (Burton et al., 2002; Nzakizwanayo et al., 2015). Nevertheless, the impact of host-microbiota signaling in influencing neurobehavioral trajectories and its implication in neurodevelopmental disorders remains unclear. One study described high levels of minerals such as As and Hg and their correlation with *Oscillospira* and *Parabacteroides* in children with ASD. Changes in the levels of trace elements have been associated with the pathophysiology of many neurological disorders such as Huntington’s disease, amyotrophic lateral sclerosis, Parkinson’s disease, Alzheimer’s disease and others (Bayir, 2015).

One of the critical factors that can influence both microbiota dynamics and nervous system integrity in neurodevelopmental disorders is the aberrant activity of the immune system and the consequent local or systemic inflammation. Increases in proinflammatory cytokine production upon TLR4 stimulation in blood cells (Rose et al., 2018), or changes in serum cytokine levels (Luna et al., 2017) have been identified in ASD subjects. High fecal calprotectin and ESR were found in RETT subjects. Only the study of Luna et al. (2017) correlated the altered serum cytokines profiles of their ASD subjects with the microbiota changes observed in intestinal tissues. These preliminary data suggest a possible link between microbiota changes and neuroimmune alterations that may affect gut-brain axis signaling and behavior in ASD and RETT. However, only some studies included in our review evaluated host-microbial metabolites, neurotransmitters, or cytokines together with microbiota analyses. For instance, all the studies included in our systematic review on ADHD assessed microbiota composition and function without investigating other factors that may contribute to host-microbiota interactions associated with the disease. Our systematic review provides clear evidence of how microbiota data in neurodevelopmental disorders may be strengthened, by conducting clinical studies using larger cohorts and including concomitant neuronal and inflammatory markers analysis.

The GI tract is colonized not only by bacteria, but also fungi, viruses, and other microorganisms that reside in mutually beneficial relationships with each other and their hosts. Perturbations in the composition of commensal bacteria can alter this inter-kingdom homeostasis, favoring the overgrowth of other microbial species that may become potentially pathogenic to the host. Strati et al. found alterations in the gut mycobiome of RETT (Strati et al., 2016) and ASD (Strati et al., 2017). Both pathologies are associated with higher abundance of intestinal *Candida* and *Aspergillus*. These results are supported by the identification of three *Candida* species in stool samples of a large cohort of pediatric ASD subjects in Turkey (Kantarcioglu et al., 2016), and anti-*Candida albicans* IgG in the blood of ASD subjects from a cohort in California, United States (Hughes and Ashwood, 2018).

Subjects from all studies evaluating the presence of GI comorbidities in ASD and RETT in our systematic review experienced constipation and abdominal pain (Kang et al., 2013, 2018; Son et al., 2015; Tomova et al., 2015; Strati et al., 2016, 2017; Borghi et al., 2017; Pulikkan et al., 2018; Rose et al., 2018; Liu et al., 2019; Zhai et al., 2019; Thapa et al., 2021). GI symptoms pose a significant burden in children with neurodevelopmental disorders (Ming et al., 2008; Adams et al., 2011). GI symptoms correlated with severity of ASD symptoms in three of 19 included studies (18 case–control studies and one case–control study with intervention). Notably, in these studies both ASD and GI symptoms were measured using three different scoring tools (Tomova et al., 2015; Kang et al., 2018; Rose et al., 2018). In agreement with previous findings (Adams et al., 2011), Kang et al. (2018) reported that GI symptoms, assessed by the modified 6-GSI score, correlated with ASD symptom severity assessed by the ATEC, which is completed by parents. However, when a physician assessed GI symptoms in ASD subjects there was no correlation between ASD symptom severity with GI symptoms (Kang et al., 2013; Son et al., 2015). Bacterial taxa were associated with constipation (Liu and Zhu, 2018; Zhou et al., 2022) or visceral pain (Luna et al., 2017) in ASD subjects.

Our review shows the presence of GI symptoms in ASD and RETT, but not in ADHD. Few studies examined the possible correlations between GI symptoms and disease severity, however, no signature of disease- or GI symptom-driven change in gut microbial communities could be identified. GI tract dysfunction could be the cause of altered microbial communities in subjects with ASD or RETT; however, this link remains unclear.

Dietary habits of NT and ASD, ADHD, and RETT patients were documented in most of the studies included in our systematic review but not included in the microbiota analysis.

Food is the primary source providing nutrients that are fundamental precursors of neurotransmitters and neuromodulators, therefore diet has a critical impact on brain health (Marrone and Coccurello, 2019). The study by Yap et al. (2021) is the only one assessing dietary and microbiota diversity in ASD subjects compared to NT siblings and unrelated NT controls. ASD subjects seem to have less dietary diversity rather than microbiota diversity. Indeed, the study reports a lower quality of food and reduced meat intake in ASD compared to NT subjects. Such dietary imbalance, especially during child development, can significantly affect the quality and quantity of macronutrient intake, for instance lipids, thus reducing the production of bioactive lipid molecules, important mediators of brain signaling pathways (Coccurello et al., 2022). The study by Thapa et al. (2021) shows that solid food intake increases intestinal bacterial diversity in RETT subjects compared to those who are fed with only formula. This functional connection between diet and microbiome needs to be further explored as could set the basis for nutritional interventions targeting the microbiota-gut ecosystem.

It is still not known if therapeutic modulation of microbiota can improve brain health and behavioral outcomes in neurodevelopmental disorders. Our systematic review identified several clinical studies investigating the effect of probiotics or FMT in ASD and ADHD. Supplementation of *Lactobacillus* species (Shaaban et al., 2018) or FMT from NT controls (Kang et al., 2017) in open-label studies have been shown to significantly improve social skills, language and communication, irritability, stereotypy, as well as to reduce constipation in children with ASD. In a follow-up study by Kang et al. (2018), 18 patients from their initial FMT-treated ASD cohort were observed for an additional 2 years. The authors describe most improvements in GI symptoms were maintained among this cohort (58% reduction in GSRS relative to baseline), and autism-related symptoms improved even further after FMT treatment ended (47% reduction in CARS at two-year follow-up, compared to 23% lower at final FMT treatment). This study was not included in our systematic review due to not meeting inclusion criteria. Further control studies are needed to discern the potential for placebo effect on GI symptoms reporting.

Pärtty et al. (2015) showed that perinatal *Lactobacillus rhamnosus GG* supplementation may reduce the risk of ADHD or ASD development in later childhood, possibly through mechanisms involving interactions between the probiotic and neuroimmune component of the MGBA such as the vagus nerve. Epidemiological data have suggested that probiotic treatment in early life may reduce risks of ADHD diagnosis at 13 years of age, while insults that promote microbial dysbiosis like antibiotic exposure and cesarean section delivery may increase risks (Curran et al., 2015; Pärtty et al., 2015; Slykerman et al., 2017). These probiotics have been extensively studied in preclinical models of gut-brain axis anomalies such as anxiety (Zhou et al., 2022), depression (Kochalska et al., 2020), and stress-induced visceral pain (McVey Neufeld et al., 2020). This may offer a promising approach to understanding the biological mechanisms linking gut microbes with neurodevelopment, and to develop novel early therapeutic interventions for neurodevelopmental disorders in the future.

## Strength and limitations

This comprehensive systematic review highlights important milestones that may guide future research on the MGBA in pediatric neurodevelopmental disorders. The most salient limitation that emerged from this review is the sample size of our included studies. Pediatric and adult-focused studies in this area remain limited, and results of our review propose valuable hypotheses that may be better assessed in larger cohorts.

The study of microbial dynamics in neurodevelopmental disorders must consider several factors beyond the primary research question. Age, stress across the lifespan, differences in dietary habits, presence of concomitant GI dysfunction, geographic location, host genetics and physiology all can influence gut microbiota composition, confounding establishment of relationships between microbes and host neuroimmune homeostasis. For example, the dietary habits of the participants were documented in only eight (of eighteen) ASD studies, three of seven ADHD studies, and two of three RETT studies. Only Yap et al. (2021) and Thapa et al. (2021) explored the link between altered dietary patterns in ASD or RETT subjects and microbiota changes. The stereotypical diet of neurodivergent subjects may play a determining role in shaping the microbiota communities, and therefore diet should always be included in the analysis.

We assessed quality of evidence by applying a semi-quantitative appraisal with CASP checklists, a widely used tool for quality appraisal in health-related studies. While informative, the CASP tool is limited by its assessment of what has been reported by a given study only (Long et al., 2020). Our use of the CASP tool offered a clear, consistent appraisal of the literature by assessing individual aspects of quality (Harrison et al., 2017).

Overall, the studies included in our search did not have a longitudinal design except the study of Pärtty et al. (2015) Thus, it is unknown if microbiome changes are a precursor to disease manifestation, or if the relationship between specific microbes and behavioral symptoms persist over time and occurred secondarily.

The choice of the most appropriate control group is fundamental to draw appropriate comparisons and represents a challenge across many microbiome studies. Differences existed across studies with use of first-degree sibling NT controls, or unrelated NT controls. A further limitation of clinical microbiome research is the impact of sampling procedure and bioinformatics analytic techniques. The 16S-rRNA gene sequencing provides information on taxonomic composition with limited resolution and poor functional readout, whereas shotgun metagenomics leads to superior sequencing and functional profiling of metabolic capacity of gut microbes (Jovel et al., 2016). Inconsistencies in sample handling, variability in sequencing methodologies and resolution, and differences in bioinformatic databases used between studies all may contribute to variability between study conclusions.

## Conclusions and future directions

In summary, our systematic review describes the presence of alterations in the gut microbiota of patients with common neurodevelopmental disorders such as ASD, ADHD, and RETT. However, these findings are inconsistent and do not support causal inferences between microbiome changes and neurodevelopmental outcomes. Future studies are required to investigate the functional role of the gut microbiota in the pathophysiology of neurodevelopmental disorders and such studies should include longitudinal design, host- and microbial-derived metabolites, dietary and physiological characteristics of the patients, and more powerful tools to sequence and analyze microbial communities.

## Data availability statement

The original contributions presented in the study are included in the article/Supplementary material, further inquiries can be directed to the corresponding author/s.

## Author contributions

VC: Conceptualization, Data curation, Formal analysis, Investigation, Methodology, Project administration, Supervision, Validation, Writing – original draft, Writing – review & editing. LH: Conceptualization, Data curation, Formal analysis, Investigation, Methodology, Project administration, Supervision, Validation, Writing – original draft, Writing – review & editing. MF: Conceptualization, Data curation, Investigation, Writing – original draft. JP: Conceptualization, Data curation, Formal analysis, Investigation, Methodology, Writing – original draft. EH: Conceptualization, Data curation, Formal analysis, Investigation, Methodology, Writing – original draft. KM: Supervision, Writing – original draft. KB: Data curation, Visualization, Writing – original draft. PJ: Data curation, Visualization, Writing – original draft. MM: Data curation, Investigation, Writing – original draft. NP: Conceptualization, Data curation, Formal analysis, Investigation, Methodology, Project administration, Supervision, Validation, Writing – original draft, Writing – review & editing.

## Appendix

Key concepts in microbiome classification and measurement.

*Microbiota* – Term used to describe all bacteria, fungi, and viruses in a given ecological space. Individual microbiota is commonly called, “taxon” or “taxa” (plural).

*Microbiome* – Term used to describe genome of all bacteria, fungi, and viruses in a given ecological space.

*Microbial Culture* – Technique used to detect specific microbiota using selective, known culture conditions.

*16S rRNA Sequencing* – Technique used to identify bacteria by PCR amplifying specific regions (i.e., V4) of the bacterial DNA, and matching amplified regions against a known reference library of “amplicons.” Resolution is often limited to genus level of taxonomic classification.

*Shotgun Metagenomics* – Technique used to detect specific organisms by sequencing all regions of the microbial genome, and matching available genes against known genomic libraries. Offers greater specificity to species and strain levels of taxonomic classification.

*Functional Metagenomics* – Term used to describe a group of techniques to determine the “functional capacity” of a microbiome. Includes: metatranscriptomics (measuring transcribed mRNA in a sample), metaproteomics (measuring available proteins in a sample), and metabolomics (measuring non-protein small molecules in a sample). Each technique provides analyses of different metabolic products produced by a microbial community and offers progressively deeper insights into how the microbiota interacts with their host.

*Inferred Metagenomics* – Similar to functional metagenomics, computational technique that uses the results of 16S rRNA Sequencing or shotgun metagenomics data to *predict* the function of a microbiota. Identified taxa are evaluated against a known library of genes for each taxon to make an indirect prediction of their functional capacity. While significantly cheaper than measures of functional metagenomics, this approach has less accuracy.

*α-diversity* – Term used to describe microbial diversity within a given ecological space. Techniques include describing the number of species found within a particular environment or describing the balance of species within the environment (or “evenness”). Examples of common α-diversity measures include Shannon, Simpson, and Chao1 indices.

*β-diversity* – Term used to describe diversity between different ecological spaces. Techniques include describing differences in number of species between two (or more) environments or describing the balance of species within the environments. β-diversity is commonly expressed visually as a principal component analysis (PCA) plot, where taxa are plotted across two to three spatial dimensions to visualize their distribution relative to one another.
