# Supplementary material for: Functional contribution of the intestinal microbiome in autism spectrum disorder, attention deficit hyperactivity disorder, and Rett syndrome: a systematic review of pediatric and adult studies
Source: Front Neurosci. 2024 Mar 7;18:1341656. doi: 10.3389/fnins.2024.1341656 (PMC10954784; doi:10.3389/fnins.2024.1341656)
Supplement: Supplementary file 1 [file Table_1.DOCX]

**S1 Table. Inclusion and exclusion criteria of reviewed studies.**

| Inclusion Criteria | |
| --- | --- |
| 1 | Articles published between January 1980 and December 2021 |
| 2 | Original data |
| 3 | Studies conducted in humans |
| 4 | Studies conducted in pediatric patients (0-18 years) |
| 5 | Studies including neuropsychiatric, neurodegenerative, or neurodevelopmental disorders |
| 6 | Studies assessing intestinal microbiome |
| 7 | Prospective, retrospective, case-control and cross-sectional study designs |
| Exclusion Criteria | |
| 1 | Studies conducted in adults (>18 years) |
| 2 | Commentaries, book chapters, letters, editorials, conference proceedings, case reports, conferences, abstracts, non-peer-reviewed publications |
| 3 | Studies conducted in animals |

NB: Criteria previously published. ^[1]^
