# Supplementary material for: Functional contribution of the intestinal microbiome in autism spectrum disorder, attention deficit hyperactivity disorder, and Rett syndrome: a systematic review of pediatric and adult studies
Source: Front Neurosci. 2024 Mar 7;18:1341656. doi: 10.3389/fnins.2024.1341656 (PMC10954784; doi:10.3389/fnins.2024.1341656)
Supplement: Supplementary file 2 [file Table_2.DOCX]

| **First Author,**  **Year** | **CASP/Checklist questionnaire Case control studies** | | | | | | | | | | | **Score/20** |
| --- | --- | --- | --- | --- | --- | --- | --- | --- | --- | --- | --- | --- |
|  | **Neuro**  **developmental**  **disorder** | **Did the study address a clearly focused issue?** | **Did the authors use an appropriate method to answer their question?** | **Were the cases recruited in an acceptable way?** | **Were the controls selected in an acceptable way?** | **Was the exposure accurately measured to minimise bias?** | **Aside from the experimental intervention, were the groups treated equally?** | **Have the authors taken account of the potential confounding factors in the design and/or in their analysis?** | **Do you believe the results?** | **Can the results be applied to the local population?** | **Do the results of this study fit with other available evidence?** |  |
| **Zhang et al. 2018** | ASD | No | Unable to assess | No | No | No | Yes | Yes | No | No | Yes | 7 |
| **Ahmed et al. 2020** | ASD | Yes | No | Yes | Yes | Yes | No | No | No | No | No | 8 |
| **Richarte et al. 2020** | ADHD | Yes | No | Yes | Unable to assess | No | Yes | No | Yes | Yes | Yes | 13 |
| **Szopinska-Tokov et al. 2021** | ADHD | Yes | No | Yes | Yes | No | Yes | No | Yes | Unable to assess | Yes | 13 |
| **Gondalia et al. 2012** | ASD | Yes | Yes | Yes | Yes | No | Unable to assess | Unable to assess | No | Unable to assess | Yes | 13 |
| **Finegold et al. 2017** | ASD | Yes | Yes | Yes | Yes | Unable to assess | Unable to assess | Unable to assess | No | Unable to assess | Yes | 14 |
| **Kantarcioglu et al. 2016** | ASD | Yes | Yes | Unable to assess | Yes | No | Unable to assess | Unable to assess | Yes | Yes | Unable to assess | 14 |
| **Kang et al. 2017** | ASD | Yes | Yes | Yes | No | Yes | Yes | Unable to assess | No | Unable to assess | Yes | 14 |
| **Luna et al. 2017** | ASD | Yes | Yes | Yes | No | Unable to assess | Unable to assess | No | Yes | Yes | Yes | 14 |
| **Shaaban et al. 2018** | ASD | Yes | Yes | Yes | No | Yes | Yes | Yes | No | Unable to assess | Yes | 15 |
| **Liu et al. 2019** | ASD | Yes | Yes | Unable to assess | Unable to assess | Yes | Yes | No | Yes | Unable to assess | Yes | 15 |
| **Kushak et al. 2017** | ASD | Yes | Yes | Yes | Yes | Yes | Yes | No | Yes | Unable to assess | No | 15 |
| **Borghi et al. 2017** | Rett | Yes | Yes | No | No | Yes | Yes | Yes | Yes | Yes | Yes | 16 |
| **Rose et al. 2018** | ASD | Yes | Yes | Yes | Yes | No | Yes | No | Yes | Yes | Yes | 16 |
| **Thapa et al. 2021** | Rett | Yes | Yes | Unable to assess | Unable to assess | Yes | Yes | Yes | Yes | Yes | Unable to assess | 17 |
| **Pulikkan et al. 2018** | ASD | Yes | Yes | No | Yes | Yes | Yes | Yes | Yes | Unable to assess | Yes | 17 |
| **Kang et al. 2013** | ASD | Yes | Yes | Yes | Unable to assess | Yes | Yes | Yes | Yes | Yes | No | 17 |
| **Hughes et al. 2018** | ASD | Yes | Yes | Yes | Yes | Yes | Yes | Unable to assess | Yes | Yes | Unable to assess | 18 |
| **Yap et al. 2021** | ASD | Yes | Yes | Yes | Yes | Yes | Yes | Yes | Yes | Yes | No | 18 |
| **Kang et al. 2018** | ASD | Yes | Yes | Yes | Yes | Yes | Yes | Yes | Yes | Yes | Yes | 20 |
| **Tomova et al. 2014** | ASD | Yes | Yes | Yes | Yes | Yes | Yes | Yes | Yes | Yes | Yes | 20 |
| **Zhai et al. 2019** | ASD | Yes | Yes | Yes | Yes | Yes | Yes | Yes | Yes | Yes | Yes | 20 |
| **De Angelis et al. 2013** | ASD | Yes | Yes | Yes | Yes | Yes | Yes | Yes | Yes | Yes | Yes | 20 |
| **Strati et al. 2017** | ASD | Yes | Yes | Yes | Yes | Yes | Yes | Yes | Yes | Yes | Yes | 20 |
| **Wan et al. 2020** | ASD | Yes | Yes | Yes | Yes | Yes | Yes | Yes | Yes | Yes | Yes | 20 |
| **Son et al. 2015** | ASD | Yes | Yes | Yes | Yes | Yes | Yes | Yes | Yes | Yes | Yes | 20 |
