# Supplementary material for: Functional contribution of the intestinal microbiome in autism spectrum disorder, attention deficit hyperactivity disorder, and Rett syndrome: a systematic review of pediatric and adult studies
Source: Front Neurosci. 2024 Mar 7;18:1341656. doi: 10.3389/fnins.2024.1341656 (PMC10954784; doi:10.3389/fnins.2024.1341656)
Supplement: Supplementary file 3 [file Table_3.DOCX]

| **First**  **Author,**  **Year** | **CASP/Checklist questionnaire Cohort Studies** | | | | | | | | | | | | **Score /20** |
| --- | --- | --- | --- | --- | --- | --- | --- | --- | --- | --- | --- | --- | --- |
|  | **Neurodevelopmental disorder** | **Did the study address a clearly focused issue?** | **Was the cohort recruited in an acceptable way?** | **Was the exposure accurately measured to minimise bias?** | **Was the outcome accurately measured to minimise bias?** | **Have the authors identified all important confounding factors?** | **Have they taken account of the confounding factors in the design and/or analysis?** | **Was the follow up of subjects complete enough?** | **Was the follow up of subjects long enough?** | **Do you believe the results?** | **Can the results be applied to the local population?** | **Do the results of this study fit with other available evidence?** |  |
| **Jiang et al. 2018** | ADHD | Yes | No | Yes | Yes | Yes | Yes | Yes | Unable to assess | Yes | No | No | 15 |
| **Wang et al. 2020** | ADHD | Yes | Yes | Yes | Yes | No | Yes | Yes | Unable to assess | Yes | No | Yes | 17 |
| **Aarts et al. 2017** | ADHD | Yes | Yes | Yes | Yes | Yes | Unable to assess | Yes | Unable to assess | Yes | Unable to assess | Yes | 19 |
| **Prehn-Kristensen et al. 2018** | ADHD | Yes | Yes | Yes | Yes | Yes | Unable to assess | Yes | Unable to assess | Yes | Unable to assess | Yes | 19 |
| **Strati et al. 2016** | Rett syndrome | Yes | Yes | Yes | Yes | Yes | Yes | Yes | Yes | Yes | Yes | Yes | 20 |
