# Supplementary material for: Functional contribution of the intestinal microbiome in autism spectrum disorder, attention deficit hyperactivity disorder, and Rett syndrome: a systematic review of pediatric and adult studies
Source: Front Neurosci. 2024 Mar 7;18:1341656. doi: 10.3389/fnins.2024.1341656 (PMC10954784; doi:10.3389/fnins.2024.1341656)
Supplement: Supplementary file 4 [file Table_4.DOCX]

| **First**  **Author,**  **Year** | **CASP/Checklist questionnaire RCT studies** | | | | | | | | | | | **Score**  **/20** |
| --- | --- | --- | --- | --- | --- | --- | --- | --- | --- | --- | --- | --- |
|  | **Neuro**  **developmental disorder** | **Did the study address a clearly focused research question?** | **Was the assignment of participants to interventions randomised?** | **Were all participants who entered the study accounted for at its conclusion?** | **Was the study methodologically sound?** | **Apart from the experimental intervention, did each study group receive the same level of care (that is, were they treated equally)?** | **Were the effects of intervention reported comprehensively?** | **Was the precision of the estimate of the intervention or treatment effect reported?** | **Do the benefits of the experimental intervention outweigh the harms and costs?** | **Will the results help locally?** | **Would the experimental intervention provide greater value to the people in your care than any of the existing interventions?** |  |
| **Stevens et al. 2019** | ADHD | Yes | Yes | Yes | Yes | Yes | Yes | Yes | Yes | Unable to assess | Yes | 19 |
| **Pärtty et al. 2015** | AS and ADHD | Yes | Yes | Yes | Yes | Yes | Yes | Yes | Yes | Yes | Yes | 20 |
