# Supplementary material for: Functional contribution of the intestinal microbiome in autism spectrum disorder, attention deficit hyperactivity disorder, and Rett syndrome: a systematic review of pediatric and adult studies
Source: Front Neurosci. 2024 Mar 7;18:1341656. doi: 10.3389/fnins.2024.1341656 (PMC10954784; doi:10.3389/fnins.2024.1341656)
Supplement: Supplementary file 5 [file Table_5.DOCX]

| **Author,**  **Year,**  **Country** | **Objectives** | **Study Type, Population, Sample Size** | **Study Methodology** | **Key Findings** | **Strengths & Limitations** |
| --- | --- | --- | --- | --- | --- |
| Zhang et al. 2018  China | Measure alterations in fecal microbiota in pediatric ASD patients and effects on host metabolism | Study Type:  Case-control study in pediatric ASD and pediatric NT controls  Population:  Pediatric ASD patients were enrolled from one local family fraternity group-a group of unrelated autistic families in Beijing (China)  Control group children were recruited from two kindergartens through paediatricians  Sample size:  35 pediatric ASD patients (mean age 4.9±1.5 yrs)  6 pediatric NT controls (mean age 4.6±1.1 yrs) | Microbiota Analysis:  16S rRNA sequencing of stool samples  ASD Diagnosis:  DSM-V  GI Symptoms:  Not assessed | 1. Ratio of Bacteroidetes/Firmicutes higher in ASD 2. Relative abundances of Bacteroidetes higher in ASD 3. *Streptococcus, Veillonella* and *Escherichia* decreased in ASD 4. Shannon index (α-diversity) showed decreased diversity in ASD 5. β-diversity showed distinct group clustering between ASD and NT controls; these differences were at phylum level 6. Butyrate- and lactate-producers increased in NT controls 7. Mucin-degraders and other SCFA-producers increased in ASD; not statistically significant but in keeping with other literature suggesting altered mucus production and intestinal permeability in ASD | Strengths:  - Good pediatric ASD sample size  - Assessed diverse taxa  Limitations:  - Some findings were not statistically significant, although trending towards significance  - Challenges with determining clinical significance of findings  -Small sample size for NT controls |
| Liu et al. 2019  China | Measure differences between pediatric ASD patients and NT controls in bacterial diversity, SCFA levels and GI symptoms | Study Type:  Case-control study in pediatric ASD and pediatric NT controls  Population:  Pediatric ASD patients were enrolled from the Fifth and Third Affiliated Hospital of Zhengzhou University (China)  Sample Size:  30 pediatric ASD patients (mean age 4.43±1.47 yrs)  20 NT controls (mean age 4.28±1.00 yrs) | Microbiota Analysis:  - 16S rRNA sequencing of stool samples  - Stool SCFAs  ASD Diagnosis:  - DSM-V edition  - ICD-10  GI Symptoms:  Modified 6-GSI | 1. Constipation higher in ASD; correlated with *Fusobacterium, Barnesiella, Coprobacter, Olsenella, Allisonella* and *Actinomycetaceae* 2. Acetic acid and butyrate decreased in ASD 3. Valeric acid increased in ASD 4. Shannon and Shannon-even indices decreased in ASD (decreased α-diversity) 5. β-diversity showed separate clustering of ASD vs NT controls 6. Firmicutes (phylum) decreased in ASD 7. *Veillonellaceae, Enterobacteriaceae* increased in ASD (family); *Ruminococcaceae, Streptococcaceae, Peptostreptococcaceae, Erysipelotrichaceae* decreased in ASD (family)  - *Ruminococcaceae, Peptostreptococcaceae, Lactobacillales, Streptococcaceae, Eubacterium., Lachnospiraceae-NC2004-group* involved in butyrate production - *Erysipelotrichaceae* decreased in ASD and associated with butyrate production  1. Butyrate decreased in ASD 2. *Acidobacteria, Enterobacteriaceae, Pseudomonadaceae, Veillonellaceae, Megamonas* increased in ASD (genus) | Strengths:  - Age-matched NT controls  - Long follow-up duration  Limitations:  - Small sample size |
| Kang et al. 2013  USA | Intestinal microbiome compared between pediatric ASD patients and NT controls | Study Type:  Case-control study in pediatric ASD and pediatric NT controls  Population:  Pediatric ASD patients  Sample Size:  20 pediatric ASD patients (mean age 6.7±2.7 yrs)  20 NT controls (mean age 8.3±4.4 yrs) | Microbiota Analysis:  16S rRNA sequencing of stool samples  ASD Diagnosis:  - ADI-R  - ADOS  - ATEC  - PDD-BI  GI Symptoms:  6-GSI  Dietary patterns  Dietary patterns (gluten-free/casein-free diet, probiotics use, seafood consumption, and usage of nutrient supplements) were documented | 1. Changes in the microbiome in ASD children were not due to different dietary pattern 2. No correlations between 6-GSI and ASD behavioral outcomes 3. NT controls had greater Shannon index (α-diversity) 4. Autistic symptoms associated with decreased bacterial diversity 5. Decreased carbohydrate-degrading genera *Prevotella, Coprococcus,* unclassified *Veillonellaceae* in ASD 6. ASD had very high abundance of genus *Akkermansia* 7. *Veillonellaceae, Prevotella, Coprococcus, unclassified Prevotellaceae* increased in NT controls; *Veillonellaceae, Prevotella, Coprococcus* strongest predictors of ASD status 8. ASD have significantly lower *Prevotella copri* compared to NT controls 9. ASD shows absence of *Prevotella*, shift in *Prevotella/Bacteroides* gradient compared to NT controls; suggests presence of autism-associated gradient shifts in microbial communities 10. Microbial changes -> more closely linked to the presence of autistic symptoms rather than to the severity of GI symptoms or special diets | Strengths:  - Extensive microbial expertise  - Good follow-up experiments  Limitations:  - Small sample size |
| Kang et al. 2018  USA | Stool metabolites compared between pediatric ASD patients and NT controls to identify correlations between stool metabolites and bacterial phylotypes | Study Type:  Prospective, case-control study in pediatric ASD and pediatric NT controls  Population:  Pediatric ASD patients enrolled at Arizona State University  Sample Size:  23 pediatric ASD patients (mean age 10.1± 4.1 yrs)  21 pediatric NT controls (mean age 8.4± .4 yrs) | Microbiota Analysis:  - 16S rRNA sequencing of stool samples  - Stool metabolite profiles assessed using NMR spectroscopy    ASD Diagnosis:  - ATEC - PDD-BI  GI Symptoms:  6-GSI | 1. 59 metabolites detected; Only isopropanol concentrations significantly higher in feces of pediatric ASD patients compared to NT controls (p=0.022) 2. Higher stool p-cresol in ASD (p=0.04) 3. p-cresol concentrations were negatively correlated with age within the ASD group, not in the NT group (Spearman correlation, r=0.47, p=0.02) 4. *Streptococcus* significantly higher in feces of NT controls (p=0.03) 5. Caprate, nicotinate, glutamine, thymine, and aspartate metabolites may serve as potential biomarkers of ASD (78% sensitivity, 81% specificity) 6. ASD patients significantly lower bacterial diversity compared to NT controls (p<0.001) 7. ASD patients significantly different microbiome from NT controls (PCoA analysis, p<0.001) 8. ASD patients exhibit lower gut microbial diversity and reduced relative abundance of *Prevotella copri* (p=0.02), *Faecalibacterium* *prausnitzii* (p<0.01), and *Haemophilus parainfluenzae* (p<0.05) 9. ASD patients significantly more severe GI symptoms compared to NT controls (p<0.005); GI symptoms correlate with ATEC scale, particularly Health/Physical/Behavior subscale (Spearman rank correlation coefficient r=0.45, 0.54; p=0.03, <0.01 for ATEC Total, ATEC-Health/Physical/Behavior subscale) | Strengths:  - Broad recruitment strategy, contacting potentially eligibile participant families virtually, then assessing eligibility of interested participants (versus recruiting from a single hospital setting only)  - First-degree sibling NT controls of ASD patients were not considered due to potential confounding from shared microbiome  Limitations:  - Sample size small for cases and NT controls  - Age and gender matching of ASD cases and NT controls ineffective |
| Rose et al. 2018  USA | Assess immune dysfunction and microbial composition in ASD children with GI symptoms | Study Type:  Case-control study on ASD and NT control children  Population:  ASD and NT control children recruited through University California Davis MIND Institute and had all been previously enrolled in the Childhood Autism Risk from Genetics and Environment (CHARGE) study ^6^  Sample Size:  4 groups (pediatric patients aged 3-12 yrs):  1-ASD with GI symptoms (ASD-GI) 20 blood samples (median age 5.71 yrs) and 21 stool samples (median age 6.58 yrs)  2- ASD without GI symptoms (ASD-NO-GI) 26 blood samples (median age 7.83 yrs) and 21 stool samples (median age 7.75 yrs)  3-NT control children with GI symptoms (NT-GI; 6 blood samples (median age 5.17 yrs) and 7 stool samples (median age 5.08 yrs)  4- NT control children without current or previous GI symptoms (NT-NO GI; 35 blood samples (median age 6.75 yrs) and 34 stool samples (median age 7.09 yrs) | Microbiota Analysis:  16S rRNA sequencing of stool samples  ASD Diagnosis:  - DSM-IV  - ADI-R  - ADOS  - SCQ  - ABC  GI Symptoms:  QPGS-RIII  Other:  -PBMC were assessed for cytokine production after stimulation with bacterial-derived products | 1. TLR4 stimulation of PBMC increases mucosal cytokines (IL-5, IL-15, IL-17) in ASD-GI compared to ASD-NO GI 2. PHA stimulation in PBMC decreased TGFβ1 (regulatory cytokine) in ASD(GI) compared to ASD-NO GI and TD-NO GI. TGFβ1 has an important role in regulating neural migration, axonal outgrowths, synapse formation, synaptic plasticity in hippocampus 3. Microbial differences between ASD and neurotypical children, irrespective of GI symptoms 4. ASD-GI overexpress zonulin, therefore increased gut permeability 5. ASD-GI had more atypical behaviour, increased irritability, agitation, social withdrawal, lethargy, hyperactivity, noncompliance compared to ASD-NO GI 6. ASD-GI had higher *Bacteriodaceae, Lachnospiraceae, Prevotellaceae, Ruminococcaceae* compared to NT-GI | Strengths:  - 4 groups to investigate the interactions between ASD and GI symptoms  - Genetic and other neurodevelopmental/neurological disorders accounted for  Limitations:  - Small sample size |
| Finegold et al. 2017  USA | Assess the presence of *C. perfringens* and *C. perfringens* toxin genes in stool samples of children with ASD compared to NT control children | Study Type:  Case-control study on ASD and NT control children  Population:  ASD children were enrolled in this study under the guidelines of the Institutional Review Board of the VA Greater LA Health Center  Sample Size:  33 pediatric ASD patients (age range 2-9 yrs) with GI symptoms  13 NT control children (age range 2-9 yrs) without ASD and without GI symptoms | Microbiota Analysis:  - Comparison of all *Clostridium* species and *Clostridium perfringens* strains from stool samples using conventional, selective anaerobic culture methods. PCR analysis was done for the main *C. perfringens* toxin genes, α, β, β_2_, ε, ι and *C. perfringens* enterotoxin gene  - Previously collected stool from cases were used  ASD Diagnosis:  Not reported  GI Symptoms:  Not reported | 1.ASD samples had statistically significantly (p=0.014) higher incidence of *C. perfringens* beta2-toxin gene  2. Mean CFU of beta2-toxin gene-producing *C. perfringens* cell count obtained from stool samples of the ASD children was higher compared to NT controls (6.55x104 CFU/g dry weight versus 1.48x103 CFU/g dry weight; p=0.015)  3. *C. perfringens* CFU/g and the CFU/g of beta2-toxin gene-producing *C. perfringens* were moderately, positively correlated (Spearman's rho = 0.7125).  4. β, ε, and ι toxin genes were not detected from cases or controls | Strengths:  - Stool samples were standardized to CFU  Limitations:  - Due to study design of using previously collected samples, it is not possible to assess for confounders  - Similarly, it is not possible to assess how well the controls were matched to the cases (i.e., exact demographics, genders, anthropometrics, related or unrelated to the cases, etc.)  - Also, unable to assess any relevant exposures during time of sample collection  - Appropriate levels of sample were unable to be gathered from 3/33 of the cases and no explanation provided for why this may have occurred  - Sample size was very small, particularly for controls. There were much fewer controls than cases (n=13 vs n=33)  - Unable to tell how the cases were determined (i.e., which neurodevelopmental tests were performed to diagnose the cases, who performed these tests, or the characteristics of this population) |
| Zhai et al. 2019  China | To investigate potential association between the level of essential and toxic elements in the body and gut microbiota in ASD patients | Study Type:  Prospective case-control study on ASD and NT control children  Population:  ASD children were enrolled from the Beijing, Jiangsu and Shandong provinces of China  Sample Size:  78 ASD children (mean age 4.96 ± 1.01 yrs)  58 age and region-matched NT control children (mean age 4.90 ± 0.97 yrs) | Microbiota Analysis:  16S rRNA sequencing of stool samples  ASD Diagnosis:  - DSM-IV  - ATEC  - ICD-10  GI symptoms:  Not assessed  Other:  - Levels of Pb, Cd, As, Cu, Zn, and Fe analysed in hair samples by inductively coupled plasma mass spectrometry | 1. Seven elements (Pb, As, Cu, Zn, Mg, Ca and Hg) were significantly higher in the ASD group compared to the control group (p<0.001 for Pb, As, Cu, Zn, Mg and Ca; p<0.05 for Hg)  2. Pb and Cd correlated positively (Spearman's rho, p<0.001) in the ASD and control groups  3. Shannon index was significantly higher in the ASD group compared to the control group (p<0.001)  4. Ratio of Bacteroidetes to Firmicutes was significantly increased in the ASD group (p<0.001)  5. Linear discriminant analysis effect size analysis showed increased relative abundance of nine genera in ASD children: *Bacteroides, Parabacteroides, Sutterella, Lachnospira, Bacillus, Bilophila, Lactococcus, Lachnobacterium* and *Oscillospira* (p<0.01, Wilcoxon rank-sum test; logLDA>3.3)  6. Redundancy analysis showed that As and Hg were significantly associated with *Parabacteroides* and *Oscillospira* in the gut  7. 40 metabolic pathways differed statistically between the ASD and NT controls | Strengths:  - p-values demonstrate significance  - Confounders appropriately considered and reported  - Appropriate statistical and bioinformatics testing performed  - Developed a model assessing microbial and metabolite profiles  Limitations:  - Power calculations not performed |
| Strati et al. 2017  Italy | Assess changes in the intestinal microbiota profiles in ASD | Study Type:  Case-control study on ASD and NT control children  Population:  ASD patients were consecutively admitted to the Child Neuropsychiatry Unit of the University Hospital of Siena (Italy)  Sample Size:  40 ASD children (36 severe cases; mean age 11.1±6.8 yrs)  40 NT control children (mean age 9.2±7.9 yrs) | Microbiota Analysis:  - 16S rRNA sequencing  - ITS1 sequencing of stool samples  ASD Diagnosis:  - DSM-V  - ADOS  - ABC  - CARS  GI Symptoms:  QPGS-RIII  Other:  Calprotectin in stool with ELISA | 1. α-diversity: no difference between ASD and NT control 2. β-diversity: bacterial communities cluster separately between ASD and controls 3. ↑*Escherichia/Shigella* and *Clostridium cluster XVIII* in constipated ASD children compared to the non-constipated one 4. ↓*Gemmiger* in constipated NT compared to non-constipated NT 5. ASD higher Firmicutes/Bacteroidetes ratio. ASD had reduced relative abundance of Bacteroidetes 6. ASD had reduced *Prevotella*, but not statistically significant 7. ASD significant increase in *Collinsella, Corynebacterium, Dorea, Lactobacillus* taxa 8. ASD significant decrease in *Alistipes, Bilophila, Dialister, Parabacteroides, Veillonella* taxa 9. Constipation in ASD and NT controls is inverse correlated with *Gemmiger, Ruminococcus*, and positively correlated with *Escherichia/Shigella, Clostridium cluster XVIII* 10. Inflammation (FCal, ESR, IgA) had no difference in ASD vs controls, or constipated vs non-constipated 11. *Candida* abundance was two-fold greater is ASD 12. Positive correlation between *Aspergillus* and *Bifidobacterium* in NT | Strengths:  - Good sample size  - Assessed bacterial and fungal changes in gut  - Correlated with inflammatory markers  Limitations:  None |
| Hughes et al. 2018  USA | Determine if antibodies are over-represented in ASD children compared to their neurotypical controls | Study Type:  Case-control study on ASD and NT control children  Population:  ASD and NT control children were enrolled in this study as part of the larger population-based cohort CHARGE mentioned previously  Sample Size:  52 ASD children (median age 7.42 yrs)  28 NT control children (median age 6.5 yrs) | Microbiota Analysis:  Measured plasma anti-*Candida* IgG levels  ASD Diagnosis:  - ADI  - ADOS  - SCQ  - ABC  GI Symptoms:  QPGS-RIII | 1. Almost 40% of anti-C. albicans+ IgG-or anti-C. albicans-I IgG- ASD children have GI symptoms 2. Almost 20% of anti-C. albicans+ IgG-or anti-C. albicans-I IgG- children have GI symptoms 3. ASD had higher percent positivity of plasma anti-*C. albicans* antibodies 4. No significant differences in GI symptoms in ASD with/without anti-*Candida* IgG 5. ASD children had more *C. albicans* in the GI tract | Strengths:  Good sample size in ASD  Limitations:  - No follow-up experiments  - Small sample size for NT control |
| Serda Kantarcioglu et al. 2016  Turkey | Identify the species of yeasts isolated from stool samples of children diagnosed or suspected autism spectrum disorder, and subsequently determine the in vitro activity of nystatin and fluconazole against these isolates | Study Type:  Prospective case-control with additional retrospective analysis of ASD and NT control children  Population:  This is a retrospective analysis of ASD patient data accumulated between 1998 – 2014 (no information on patients’ recruitment)  Sample Size:  1555 ASD children (415 samples analysed from December 2011 and December 2014)  403 NT control children.  Age range 9 months-18 yrs (mean age per group not reported) | Microbiota Analysis:  - Yeast strains via direct microscopy and culture  - Retrospective analysis of patient data accumulated between 1998 – 2014 about *C. albicans* was also conducted by year  - Susceptibility patterns of the isolates strain to nystatin and fluconazole were determined by broth microdilution assays and MIC was determined  ASD Diagnosis:  Not reported  GI Symptoms:  Not assessed | 1. Isolated 338 yeast strains from 415 stool samples from ASD patients – *Candida* species were the most common yeasts (97.9%), of these species, *C. albicans* was significantly higher in number than any other (43.2%) 2. No yeast strains were cultured from most control samples, isolates were found in 19.6% and most species identified were *C. albicans* (58.2%). 3. The rate of yeasts found was significantly lower in NT patients (p<0.05) 4. In the retrospective analyses 1130 strains were isolated, *C. albicans* being the most common (57.4) every year of the 17 years included in the analysis 5. All *C. albicans*, *C. tropicalis* and *C. parapsilosis* strains were susceptible to fluconazole; resistance to fluconazole was found in *C. krusei* and high MIC values in *C. glabrata* strains 6. All *C. albicans*, *T. mucoides* and *S. cerivisiae* strains showed low MIC values against nystatin 7. Low MIC50 values were observed for *C. tropicalis* and *C. parapsilosis* | Strengths:  - Very large number of samples were analysed prospectively, and retrospectively  - One of few studies looking for presence of *Candida* species in paediatric population  - Presence of *C krusei* and C glabrata in only ASD patients have been corroborated  - Did not accept samples of those who drank kefir in previous 2 weeks to avoid misinterpretation of presence of *Candida* isolates  - Used several assays to characterize yeast isolates  Limitations:  Large p-values, statistical significance of findings unclear |
| Kushak et al. 2017  USA | Assess the gut microbiota composition in duodenal mucosa-associated biopsies from ASD children and to correlate gut microbiota alterations with the activity of digestive enzymes | Study Type:  Case-control study on ASD and NT controls  Population:  All ASD and NT controls had undergone an upper GI endoscopy under general anesthesia at the Pediatric GI and Nutrition Unit (Massachusetts, US) for evaluation of suspected GI disorders  Sample Size:  21 ASD children (mean age 14.43 ± 1.07 yrs)  19 NT controls (mean age 16.05 ± 1.25 yrs) | Microbiota Analysis:  16S rRNA sequencing of duodenum mucosal biopsies  ASD Diagnosis:  DSM-VI edition  GI symptoms  Upper GI endoscopy  (Constipation and gastroesophageal reflux in ASD children, but also in NT children (UC in two of them)  Other:  Disaccharidase activity assay on duodenal biopsies. | 1. All participants had GI symptoms (constipation, abdominal pain, diarrhea, or GERD) 2. ASD had higher constipation and GERD 3. No difference in disaccharidase, lactase, sucrase, maltase, and palatinase activities between ASD and NT children 4. No difference in intestinal microbiota diversity [richness (OTUs) or diversity (Shannon index)] 5. Age and sex did affect OTUs and Shannon index 6. Genus *Burkholderia* increased in ASD 7. Genus *Neisseria* decreased in ASD 8. ASD decrease in species *Bacteroides, Escherichia* 9. Positive correlation between ASD, disaccharidase activity, *Clostridium* species 10. ASD higher *Oscillospira, Actinomyces, Neisseria, Peptostreptococcus, Ralstonia* 11. ASD had lower *Devosia, Prevotella, Bacteroides, Streptococcus* | Strengths:  All participants underwent upper GI endoscopy for evaluation of GI disorders  Limitations:  - ASD group uneven distribution of sex (19 boys, 2 girls)  - Small sample size  - Dietary confounders; ASD only 29% on normal diet, neurotypical had 58% on normal diet  - Heterogenous age, sex, GI symptoms to control-match  - No control group without autism and GI symptoms |
| Luna et al. 2017  USA | Investigate the link between intestinal microbiome-neuroimmune signatures and functional abdominal pain in children with ASD | Study Type:  Case-control study on ASD and NT control children  Population:  Study participants were recruited from the outpatient paediatric GI procedure suite at Nationwide Children’s Hospital in Columbus, Ohio  Sample Size:  14 ASD children with FGID (median age 8.5 yrs)  15 NT with FGID (median age 10.5 yrs)  6 NT without FGID (median age 9 yrs) | Microbiota Analysis:  16S rRNA sequencing of rectum mucosal-associated bacteria  ASD Diagnosis:  - ADOS  - SRS  GI Symptoms:  QPGS-RIII  Other:  - Cytokines measurements in blood and biopsy supernatants  Neurotransmitters measurements in biopsy supernatants and biopsies specimens (with HPLC) | 1. Difference in the composition of bacteria communities between ASD-FGID and NT-FGID or NT 2. Increases in Clostridiales species such as *Clostridium lituseburense* (p=0.002), *Lachnoclostridium bolteae,* (p=0.017), *Lachnoclostridium hathewayi* (p=0.030), *Clostridium aldenense* (p=0.038), and *Flavonifractor plautii,* (p=0.038) in the ASD-FGID group compared with the NT-FGID and NT groups 3. Decreases in *Dorea formicigenerans* (p=0.006) and *Blautia luti* (p=0.020), *Sutterella species* (p=0.025), were also observed in the ASD-FGID group 4. Increases in *Faecalibacterium prausnitzii*, *Roseburia intestinalis*, *Oscillospira valericigenes*, and *Bilophila wadsworthia* in the NT-FGID group relative to the NT group 5. Specific bacterial species correlated with GI symptoms (independent of ASD) and there was no significant overlap between organisms associated with specific GI symptoms and organisms contributing to the separation of the ASD and NT groups 6. Six bacterial taxa were predominant in ASD-pain compared to ASD-no pain and NT (with or without visceral pain) 7. Lower tryptophan levels in the supernatant from ASD-FGID biopsies compared with either the NT-FGID (p<0.006) or the NT group (p< 0.009). 8. Higher 5-HIAA levels in ASD-FGID biopsies compared to NT (p<0.009; this increase was associated with visceral pain (p<0.039). 9. In the blood, lower GROα, IFN-a2 and higher MCP-1 and eotaxin levels were observed in ASD-GI patients compared with NT-GI and NT. Increases of MCP-1 and eotaxin were associated with abdominal pain in ASD-FDIG 10. Positive correlations between serotonin levels in ASD-FGID biopsies or biopsy supernatants and bacterial taxa 11. Positive correlations between blood/mucosa associated cytokines and bacteria associated with ASD-FGID and ASD-FGID-pain | Strengths:  - All participants underwent GI colonoscopy and filled the QPGS-RIII for evaluation of FGID  - Control group with and without GI symptoms  Limitations:  - Small sample size  - Large range of age  - All the groups include male participants except for the NT-FGID which includes 3 females  - Absence of ASD group without GI symptoms |

**Abbreviations:** ABC = Aberrant Behavior Checklist; ADI-R = Autism Diagnostics Interview Revised; ADOS-CSS = Autism Diagnostic Observation Schedule Calibrated Severity Score; AIIMS = All India Institute of Medical Science; ATEC = Autism Treatment Evaluation Checklist ; ASD = Autism Spectrum Disorder; ASD-GI = Autism Disease with GI symptoms; BMI = Body Mass Index; BSC = Bristol Stool Chart; bTEFAP = Bacterial tag-encoded FLX amplicon pyrosequencing; CARS = Childhood Autism Rating Scale; CBCL/6-18 = Child Behavior Checklist ages 6-18; CFU = Colony Forming Unit; DNA = Deoxyribonucleic Acid; DSM = Diagnostic and Statistical Manual of Mental Disorders; ELISA = Enzyme-Linked Immunosorbent Assay; ESR = Erythrocyte Sedimentation Rate; FCal = Faecal Calprotectin; FDA =Fisher Discriminant Analysis; FDR = False Discovery Rate; FGID = Functional Gastrointestinal Disorders; GI = Gastro Intestinal; GERD = Gastroesophageal Reflux Disease; GROα = Growth-Related Oncogene Alpha; 6-GSI = 6-Item Gastrointestinal Severity Index; 5-HIAA = 5-Hydroxyindoleacetic acid; HPLC = high-performance liquid chromatography; ICD-10 = International Classification of Diseases-10; IFN = Interferon; Ig= Immunoglobulin; IL = Interleukin; INDT-ASD = INCLEN Diagnostic Tool for Autism Spectrum Disorder; IQ-DQ = Intelligence Quotient-Developmental Quotient; IRB = Institutional Review Board; ISAA= Indian Scale For Assessment Of Autism; ITS1 = Internal Transcribed Spacer; MCP-1 = Monocyte Chemoattractant Protein; MIND = Medical Investigation Of Neurodevelopmental Disorders; NMR = Nuclear Magnetic Resonance; NT = Neurotypical; OTUs= Operational Taxonomic Units; PC = Principal Components; PcoA = Principal Coordinates Analysis; PBMC = Peripheral Blood Mononuclear Cells; qPCR = Quantitative Polymerase Chain Reaction;; PDD-BI = Pervasive Developmental Disorder Behavior Inventory; PHA= phytohemagglutinin (a T-cell activator); QPGS-RIII = Questionnaire On Pediatric Gastrointestinal Symptoms-Rome III; rRNA = Ribosomial Rybonucleic Acid; SCFAs = Short Chain Fatty Acids; SCQ = Social Communication Questionnaire; SRS = Social Responsiveness Scale; TGF = Transforming Growth Factor; TLR4 = Toll-Like Receptor 4; TNF = Tumor Necrosis Factor; Yrs = Years.

**Notes:** **Ruminococcaceae* was amended in *Oscillospiraceae* in 2019.
