# Supplementary material for: Functional contribution of the intestinal microbiome in autism spectrum disorder, attention deficit hyperactivity disorder, and Rett syndrome: a systematic review of pediatric and adult studies
Source: Front Neurosci. 2024 Mar 7;18:1341656. doi: 10.3389/fnins.2024.1341656 (PMC10954784; doi:10.3389/fnins.2024.1341656)
Supplement: Supplementary file 6 [file Table_6.DOCX]

| **Author,**  **Year,**  **Country** | **Objectives** | **Study Type, Population, Sample Size** | **Study Methodology** | **Key Findings** | **Strengths & Limitations** |
| --- | --- | --- | --- | --- | --- |
| Son et al. 2015  USA | Assess associations between ASD and FGID | Study Type:  Case-control study on ASD and NT first-degree siblings  Population:  Families were recruited via a registry called the Simons Simplex Community through the Interactive Autism Network (SSC@IAN), which is composed of families originally recruited to the Simons Simplex Collection that were willing to be contacted through the Interactive Autism Network for additional studies. Cases and controls were predominantly Caucasians  Sample Size:  59 pediatric ASD patients (mean age 10.3±1.8 yrs)  44 NT first-degree siblings (matched ASD and NT sibling pairs were from 37 families; mean age 10±1.8 yrs) | Microbiota Analysis:  - 16S rRNA sequencing of stool samples  - qPCR for targeted bacterial subgroups  ASD Diagnosis:  CBCL/6-18  GI Symptoms:  QPGS-RIII | 1. ASD children exhibited a higher prevalence of functional constipation in compared to NT first-degree siblings (p=0.035) when both matched and unmatched individuals were included in the analysis 2. No differences in ASD severity scores (ADOS-CSS and CBCL between ASD with FGID and ASD without FGID 3. No difference in the abundance of *Sutterella*, *Prevotella* and total Bacteroidetes (assessed trough targeted qPCR assays of stool samples) between ASD and NT siblings, with or without FGID 4. No significant effect of ASD, FGID or ASD-FGID on microbial α-and β-diversities 5. Associations between microbiota and FGDI:  - ASD and ASD*FGID with *Asteroleplasma* (Firmicutes) - ASD, FGID and ASD*FGID with *Thalassospira* (Proteobacteria) - FGID and ASD*FGID with *Burkholderia* (Proteobacteria) | Strengths:  Good sample size, use of first-degree siblings to eliminate some environmental differences of a traditional matched-case control design  Limitations:  -59 ASD, 44 NT first-degree siblings came from 66 families. Most participants were first-degree siblings. Concerns for bacterial diversity among family members, and how NT status of first-degree siblings was assessed  -ASD patients were predominantly male  -Participants were predominantly Caucasian; concerns regarding generalizability of results across populations |
| Gondalia et al. 2012  Australia | Identify differences and similarities in the microbiota of children with autism (with and without gastrointestinal dysfunction) and their NT first-degree siblings | Study Type:  Prospective case-control study on ASD and NT first-degree siblings  Population:  Participants were recruited through notices posted on  websites and newsletters of various local state and national bodies related to autism in Australia  Sample Size:  51 ASD children  (n=23 without GI dysfunction, n=28 with GI dysfunction)  53 NT first-degree siblings  Age range 2-12 yrs (mean age per group not reported) | Microbiota Analysis:  - bTEFAP sequencing in stool samples  ASD Diagnosis:  CARS  GI Symptoms:  - Family-filled questionnaire | 1. Pyrosequencing of all specimens resulted in a total of 4000 sequences per sample, and covered 19 bacterial phyla and 666 species 2. Found that the estimate of operational taxonomic unit richness compared between the intestinal microbiome of those with ASD versus the NT first-degree siblings was not significant 3. The relative abundance of the dominant phyla in NT vs. mild and severe ASD patients was not significant 4. No difference in the relative abundance at phylum and genus level between ASD and NT first-degree siblings with or without GI symptoms | Strengths:  -Use of more advanced bacterial sequencing technology compared to that used in similar previous studies  -Good sample size, and use of first-degree siblings to eliminate some environmental differences of a traditional matched-case control design  Limitations:  -Use of first-degree siblings as NT controls; not age and sex-matched (except for one case of identical twins)  -Use of only one method for prior assessment of autism (CARS) and associating this with GI dysfunction  -Did not account for any dietary differences  -No assessment of microbial end products/metabolites |
| De Angelis et al. 2013  Italy | Compare ASD and PDD-NOS to NT controls, stool microbiota and metabolome | Study Type:  Case-control study on ASD and NT first-degree siblings  Population:  This study was approved by the Institutional Review Board of Azienda Ospedaliero-Universitaria Consorziale Policlinico di Bari (Italy). ASD and PDD-NOS children referred to the Child Neurological and Psychiatric Unit of Bari University Hospital  Sample Size:  10 ASD children  10 PDD-NOS children  10 NT first-degree siblings  Age range 4-10 yrs (mean age per group not reported) | Microbiota Analysis:  - bTEFAP analysis in bacterial DNA and cDNA (from RNA) in stool samples  - enumeration of cultivable bacteria by using selected media  ASD and PDD-NOS diagnosis:  - ADI-R  - ADOS  - CARS  GI Symptoms:  Not assessed  Other  -Analysis of total and individual free amino acids contained in stool samples.  -Analysis of volatile compounds with gas-chromatography mass spectrometry | 1. Shannon index (α-diversity) higher in PDD-NOS and ASD compared to controls 2. Firmicutes lower in ASD compared to NT controls 3. Bacteroidetes higher in ASD compared to NT controls 4. Fusobacteria and Verrucomicrobia lower in ASD and PDD-NOS compared to NT controls 5. *Clostridiaceae* species were highest in ASD children and are associated with toxic effects and potentially ASD symptoms 6. Bacteroidetes genera (*Bacteroides, Barnesiella, Odoribacter and Parabacteroides*), *Prevotella* and *Alistipes* species were highest in ASD children. *Bacteroides* species are known propionate-produces, which may have neurotoxic effects 7. the average value of total short and medium chain fatty acids was significantly (p<0.05) higher in NT controls than in PDD-NOS and, especially, ASD 8. acetic and propionic acids were found at the highest levels in ASD and PDD-NOS 9. Analysis of cultivable bacteria: no statistical difference was found between PDD-NOS, ASD and NT for total microbes.   in ASD compared with NT  ↑presumptive *Clostridium, Bacteroides, Porphyromonas, Prevotella, Enterobacteriaceae, Pseudomonas, Aeromonas*  ↓presumptive *Enterococcus, Lactobacillus, Streptococcus, Lactococcus, Staphylococcus, Bifidobacterium* | Strengths:  - Extensive panel of bacteria studies  - Good follow-up experiments  Limitations:  Small sample size |
| Pulikkan et al. 2018  India | Study the gut microbiota of children with ASD | Study Type:  Case-control study on ASD and NT controls  Population:  ASD children were recruited from the Sunrise Hospital in Kerala (southern India)  All children were on native diet  Sample Size:  30 ASD children  24 age-matched and mostly NT first-degree siblings  Median age 9.5 yrs (age range 3-16 yrs) | Microbiota Analysis:  - 16S rRNA sequencing of stool samples  - Comparative analysis done used ASD dataset from a USA population database to compare key taxa associated with ASD children  ASD Diagnosis:  - AIIMS-modified - (DSM-V approved)  - INDT-ASD  - ISAA  GI Symptoms:  Not assessed | 1. BMI significantly lower in ASD children (p=0.02)  2. ASD group significantly higher males (p=0.0063)  3. Shannon index indicated that the stool microbial diversity of ASD group was like that of the NT controls. Phylogenetic diversity showed no significant β-diversity (p=0.8)  4. Autism covariate correlated significantly with PC3 (p<0.05), explaining the differences in microbiota between cases and controls. BMI showed no significant correlation with any of the principal components  5. Correlation analysis of families showed the high PC3 values positively correlated with increased abundances of *Lactobacillaceae, Mogibacteraceae,* and *Enterococcaceae* families and were negatively correlated with a higher abundance of Prevotellaceae family (p<0.1). *Prevotellaceae*, *Lactobacillaceae*, and *Mogibacteraceae* were also significant different in ASD versus NT controls  6. The proportion of Firmicutes (56 ± 37%; mean = 56%; n = 30) was higher in ASD children as compared to NT controls (37 ± 28%; mean = 37%; n = 24), which may be contributing to increased intestinal permeability  7. Higher abundances of *Prevotellaceae* (37.8%) in NT controls versus ASD children (28.04%). Higher abundance of *Veillonelleaceae* in ASD children (11.38%) versus NT controls (6.31%)  8. Significant difference included: higher relative abundance of *Lactobacillaceae* (p=0.018*), Bifidobacteraceae* (p=0.0054), and Veillonellaceae (p=0.008) in ASD children compared to NT controls. Significantly lower *Erysipelotrichaceae* (p=0.0005), *Enterococcaceae* (p=0.0127), and *Desulfovibrionaceae* (p=0.03) in ASD versus controls  9. Significant genera differences: higher relative abundance of *Bifidobacterium* (p=0.005), *Lactobacillus* (p=0.018), *Megasphaera* (p=0.0008), and *Mitsuokella* (p=0.007) in ASD children as compared to NT controls  10. Phylogenetic plot of the taxa of discriminatory OTUs displayed an abundance of *Prevotella* from family *Prevotellaceae, Faecalibacterium* from family *Clostridiaceae*, and *Roseburia* from family Lachnospiraceae in NT controls, whereas *Ruminococcus* from family *Ruminococcaceae,* *Coprococcus*, and *Butyrivibrio* from family *Lachnospiraceae*, and *Klebsiella* from family *Enterococcaceae* were found abundant in ASD children  11. *Lactobacillus* to be significantly higher (FDR adjusted p<0.01) in ASD children as compared to NT controls in both populations (India and USA) | Strengths:  Authors considered covariates such as autism, BMI, and age on the taxonomic profiles  Limitations:  - No power calculations were performed  - Cases were not representative of the ASD population as a whole, as the investigators included only severely affected children to understand the disorder at its extreme. But this does not consider the dietary restrictions or notable food preferences such children will have. It also does not consider the environmental exposures between the two groups, such as limited exposure to day-care or grade school among the cases  - NT controls may not have been the most appropriate choice as they were family members. It is known that co-habitants tend to share microbial signatures. Better controls could have been unrelated age-matched controls |
| Ahmed et al. 2020  Egypt | Study intended to investigate changes in intestinal microbiome and correlate with severity of ASD and GI symptoms | Study Type:  Case-control study on ASD and NT control children (both first-degree siblings and family unrelated)  Population:  ASD children were recruited from Autism Clinic of Alexandria University Children’s Hospital (Egypt).  Sample Size:  41 ASD children (6 severe, 35 mild and moderate ASD; mean age 5.55±1.9 yrs)  45 NT first-degree siblings (mean age 4.3±3.23 yrs)  45 unrelated NT individuals (mean age 5.36+2.61 yrs) | Microbiota Analysis:  Real-Time PCR for bacterial taxa in stool samples  ASD Diagnosis:  - DSM-V  - CARS  GI Symptoms:  Modified 6-GSI | 1. Mean GSI score was 3.37 + 2.12. Abnormal stool smell, flatulence, constipation, abdominal pain, abnormal stool consistency and diarrhea were the most common symptoms for affected patients 2. Phylum analysis showed no significant difference between relative abundance of Bacteroidetes and Firmicutes between all groups (p-0.456, p=0.233), although ratio between Firmicutes and Bacteroidetes was lower in ASD cases and first-degree siblings compared to NT controls (p=0.028, p=0.002) 3. Genus level analysis showed greater *Bacteroides* and *Ruminococcus* abundance in ASD and first-degree siblings compared to NT controls (p<0.001, p=0.003) 4. Pairwise comparison but not relative abundance comparisons revealed significant difference in *Prevotella* species between groups, and *Prevotella/Bacteroides* ratio was lower in ASD patients and first-degree siblings compared to NT controls (p<0.001, p=0.001). 5. On species level analysis, *Bifidobacterium spp*. relative abundance was higher in first-degree siblings than ASD and NT controls group (p=0.05). 6. *C. difficile* did not vary among groups, *Desulfovibrio spp.* was non-significantly higher in ASD cases compared to other groups, and *Sutterella spp.* relative abundance was higher in ASD group (p=0.07, p=0.801, p=0.49). 7. No statistical difference between mild/moderate ASD with severe ASD in age, gender, CARS score, GI symptoms, GSI score 8. Only weight was lower in severe cases (p=0.013). Severe ASD patients had greater abundance of *Firmicutes* compared to mild-moderate patients (p=0.027) but other bacterial species/ratios not significant 9. No significant correlation between relative abundance of  *Bifidobacterium spp*. and severity of autism (CARS) demonstrated (p>0.05). No significant difference between bacterial species and 6-GSI score (p>0.05). Shannon diversity measures and similar index not significant between cases and control group (p>0.05) | Strengths  - Patients with diabetes, inflammatory bowel disease, hepatic disorders and known immune deficiency, food intolerances were excluded from study and those taking probiotics  Limitations:  - Biopsies not employed, only single stool samples  - Small sample size especially for severe-ASD group (n=6) |
| Yap et al. 2021  Australia | Study undertook stool metagenomics study to quantify relationship between intestinal microbiome and ASD diagnosis | Study Type:  Case-control study on ASD and NT control children (both siblings and family unrelated)  Population:  ASD children referred to the Australian Autism Biobank and Queensland Twin Adolescent Brain Project (Australia)  Sample Size:  99 ASD children (mean age 8.7±3.8 yrs)  51 paired NT first-degree siblings (mean age 8.0±4.3 yrs)  97 unrelated NT controls (mean age 9.2±3.7 yrs) | Microbiota Analysis:  Metagenomic sequencing of stool samples  ASD Diagnosis:  - ADOS-2  - ADOS-G  GI Symptoms  Stool consistency measured with Bristol Stool Chart  Other:  Dietary records were collected from Australian Eating Survey | 1. Stool microbiome is not associated with ASD diagnostic status or IQ-DQ results, sleep problems and other neurodevelopmental traits associated with ASD 2. ↓diet quality in ASD compared to NT siblings and NT unrelated 3. ↓meat intake in ASD compared to NT siblings and NT unrelated 4. Limited evidence for associations of stool microbiome composition with taxonomic diversity, or microbiome-association index 5. Intestinal microbiome composition is strongly associated with age, dietary intake, and stool consistency features 6. ASD-related interests or behaviours (i.e., sensory preferences) are associated with less diverse diet, lower taxonomic diversity, and lower stool consistency (measured with BSC) | Strengths:  - 3 study groups were matched by age (p=0.2)  - Confounders considered  Limitations:  - Single stool samples taken at single time points, does not reflect chronic conditions.  - Lack of longitudinal design, intestinal microbiome contributions prior to ASD diagnosis unknown  - Sampling biases  - Stool samples used as proxy for intestinal microbiome proxy, not representative of mucosal microbiome  - Data on antibiotic intake not systematically collected, was not accounted for  - Per-feature tests do not reflect interactions/independence occurring in ecological/metabolic contexts |

**Abbreviations:** ABC = Aberrant Behavior Checklist; ADI-R = Autism Diagnostics Interview Revised; ADOS-CSS = Autism Diagnostic Observation Schedule Calibrated Severity Score; AIIMS = All India Institute of Medical Science; ATEC = Autism Treatment Evaluation Checklist ; ASD = Autism Spectrum Disorder; ASD-GI = Autism Disease with GI symptoms; BMI = Body Mass Index; BSC = Bristol Stool Chart; bTEFAP = Bacterial tag-encoded FLX amplicon pyrosequencing; CARS = Childhood Autism Rating Scale; CBCL/6-18 = Child Behavior Checklist ages 6-18; CFU = Colony Forming Unit; DNA = Deoxyribonucleic Acid; DSM = Diagnostic and Statistical Manual of Mental Disorders; ELISA = Enzyme-Linked Immunosorbent Assay; ESR = Erythrocyte Sedimentation Rate; FCal = Faecal Calprotectin; FDA =Fisher Discriminant Analysis; FDR = False Discovery Rate; FGID = Functional Gastrointestinal Disorders; GI = Gastro Intestinal; GERD = Gastroesophageal Reflux Disease; GROα = Growth-Related Oncogene Alpha; 6-GSI = 6-Item Gastrointestinal Severity Index; 5-HIAA = 5-Hydroxyindoleacetic acid; HPLC = high-performance liquid chromatography; ICD-10 = International Classification of Diseases-10; IFN = Interferon; Ig= Immunoglobulin; IL = Interleukin; INDT-ASD = INCLEN Diagnostic Tool for Autism Spectrum Disorder; IQ-DQ = Intelligence Quotient-Developmental Quotient; IRB = Institutional Review Board; ISAA= Indian Scale For Assessment Of Autism; ITS1 = Internal Transcribed Spacer; MCP-1 = Monocyte Chemoattractant Protein; MIND = Medical Investigation Of Neurodevelopmental Disorders; NMR = Nuclear Magnetic Resonance; NT = Neurotypical; OTUs= Operational Taxonomic Units; PC = Principal Components; PcoA = Principal Coordinates Analysis; PBMC = Peripheral Blood Mononuclear Cells; qPCR = Quantitative Polymerase Chain Reaction;; PDD-BI = Pervasive Developmental Disorder Behavior Inventory; PDD-NOS = Pervasive Developmental Disorder Not Otherwise Specified; QPGS-RIII = Questionnaire On Pediatric Gastrointestinal Symptoms-Rome III; rRNA = Ribosomial Rybonucleic Acid; SCFAs = Short Chain Fatty Acids; SCQ = Social Communication Questionnaire; SRS = Social Responsiveness Scale; TGF = Transforming Growth Factor; TLR4 = Toll-Like Receptor 4; TNF = Tumor Necrosis Factor; Yrs = Years.

**Notes:** **Ruminococcaceae* was amended in *Oscillospiraceae* in 2019.
