# Supplementary material for: Functional contribution of the intestinal microbiome in autism spectrum disorder, attention deficit hyperactivity disorder, and Rett syndrome: a systematic review of pediatric and adult studies
Source: Front Neurosci. 2024 Mar 7;18:1341656. doi: 10.3389/fnins.2024.1341656 (PMC10954784; doi:10.3389/fnins.2024.1341656)
Supplement: Supplementary file 7 [file Table_7.DOCX]

| **Author,**  **Year,**  **Country** | **Objectives** | **Study Type, Population, Sample Size** | **Study Methodology** | **Key Findings** | **Strengths & Limitations** |
| --- | --- | --- | --- | --- | --- |
| Jiang et al. 2018  China | Define the characteristics of gut microbiota in treatment-naive children with ADHD and to assess their relationship with the severity of ADHD symptoms | Study Type:  Prospective cohort study, of children with ADHD and NT controls  Population:  Patients recruited from a child and adolescent outpatient clinical center at Seventh People’s Hospital of Hangzhou, Zhejiang, China, from May 2015 to December 2016  Sample Size:  51 treatment-naive ADHD patients (mean age 8.47± 8.47 yrs  32 NT controls (mean age 8.5±8.47 yrs) | Microbiota Analysis:  16S rRNA sequencing of stool samples  ADHD Diagnosis:  -Kiddie-SADS-Present and Lifetime Version (Kiddie-SADS-PL)  -all ADHD patients were medication-naïve  GI Symptoms:  Not assessed | 1. There were no significant differences in β-diversity between the ADHD and NT group 2. route of delivery (vaginal or cesarean) and early nutrition (breast or formula milk) did not affect alpha and beta diversity of the ADHD microbiota 3. There were lower abundances of *Alcaligenaceae* and greater abundance of *Peptostreptococcaceae* in the ADHD group compared to the NT group 4. Three genera differed between the two groups: *Faecalibacterium*, *Lachnoclostridium*, and *Dialister* were significantly decreased in the ADHD groups | Strengths:  - Only treatment- naïve ADHD juveniles were included, which excluded the impact of treatment on the microbiota profiles  - Relatively large sample size  Limitations:  - Cross-sectional design prevents conclusions about causality |
| Prehn-Kristensen et al. 2018  Germany | Assess the microbiome composition in young ADHD patients | Study Type:  Prospective cohort study, of children with ADHD and NT controls  Population:  All participants and their parents were Caucasians. All family members were born and raised in Germany. The parents of ADHD patients and NT controls were also included  Sample Size:  *Patients*  14 ADHD patients (mean age 11.9±2.5 yrs)  17 NT controls (mean age 13.1± 1.7 yrs)  *Parents*  ADHD: 9 both parents, 4 only the mother, 1 only the father  NT controls: 12 both parents, 5 only the mother | Microbiota Analysis:  16S rRNA sequencing of stool samples  ADHD Diagnosis:  -German translation of the Revised K-SADS-PL  -CBCL  -FBB-HKS or German ADHD rating scale  -Ten ADHD patients had been taking medicine for more than one year to treat ADHD symptoms. Nine of them discontinued taking the medicine for at least 48h prior to stool sample collection.  GI Symptoms:  Not assessed | 1. α-diversity (Shannon index) was significantly reduced in ADHD patients compared to controls (p= 0.036) 2. β-diversity differences between ADHD and NT controls (p_ANOSIM_=0.033, p_ADONIS_=0.006) 3. α-diversity correlated with levels of hyperactivity (hyperactivity: r=-0.35, p=0.03) 4. Mothers of ADHD patients also showed reduced α-diversity, and self-reported greater ADHD symptoms than mothers of NT controls 5. No differences were found in α-diversity and ADHD self-ratings in fathers of ADHD patients or NT controls 6. Patients with ADHD showed elevated levels of *Bacteroidaceae, Neisseriaceae*, and *Neisseria spp*. compared to NT controls 7. Potential role for Bacteroides in producing complex mixtures of amyloids, lipopolysaccharides, enterotoxins and neurotoxins, which can affect the intestinal and the BBB as well as the enteric and central nervous systems | Strengths:  - Parents included in analysis  - Similarities between parents, and control groups with active group with respect to self-reported dietary intake  Limitations:  - Self-reported dietary intake affected by reporting biases  - Concomitant medication: Ten of 14 patients had taken methylphenidate, the first-line treatment of ADHD, for more than one year. Nine of them discontinued taking the medication at least 48h (approximately twelve half-lives) prior to sample collection  - Small sample size  - Female patients needed for inclusion to investigate possible gender effects as indicated by the parental microbiome (mothers) |
| Wang et al. 2020  Taiwan | Determine whether imbalanced gut microbiomes are involved in the pathophysiology of ADHD | Study Type:  Prospective cohort study of children with ADHD and NT controls  Population:  Eligible patients with ADHD treated in the outpatient Department of Child Psychiatry at Chang Gung Children’s Hospital in Taiwan and healthy NT children  Sample Size:  30 ADHD patients (mean age 8.4years±1.7 yrs)  30 NT controls (mean age 9.3±2.2 yrs)  Notes: iii) children with special dietary habits (vegetarian and non-vegetarian) were not included in the study | Microbiota Analysis:  16S rRNA sequencing of stool samples  ADHD Diagnosis:  -K-SADS-E  -WISC-IV  -SNAP-IV completed by teachers and parents  -all ADHD patients included in the study have never taken any medication  GI Symptoms:  Not assessed | 1. At the phylum level, the bacterial composition of both groups did not show any significant differences 2. Phylum *Fusobacteria* was higher in the ADHD group than NT controls (mean=0.28% vs. mean=0.02%; p=0.041) 3. *Bacteroides uniformis* *(B. uniformis), Bacteroides ovatus (B. ovatus),* and *Sutterella stercoricanis (S. stercoricanis)* were increased in the ADHD group 4. *Bacteroides coprocola* was decreased compared to NT controls 5. Compared to NT controls, children with ADHD had a higher intake of refined grains (p=0.027) and a lower proportion of dairy (p=0.020) and vitamin B2 (p=0.033) 6. *S. stercoricanis* was significantly associated with dairy, nut, seed, and legume intake 7. *B. uniformis* correlated with fat and carbohydrate intake 8. *S. stercoricanis* and *B. ovatus* positively correlated with ADHD symptoms | Strengths*:*  - Dietary patterns were assessed  Limitations*:*  - Food frequency questionnaires that involve self report often are subject to reporting biases  - Cross-sectional design, preventing assessment of past cumulative risk factors. Unable to depict the longitudinal change and associated factors of gut microbiota  - Low symptom severity compared to other populations of ADHD  - Vegetarians were excluded in the current study. Our study result may not be applicable in those with different diet patterns such as gluten free or vegan |
| Wan et al. 2020  China | Investigate the effects of gut microbiota composition on neurotransmitter metabolic pathways in children with ADHD | Study Type:  Case-control study of patients with ADHD and NT controls  Population:  Eligible patients were referred from Paediatric Outpatient Department of First Medical Center of Chinese PLA General Hospital between January and June 2019  Sample Size:  17 patients with ADHD (median age 8 yrs; 25th and 75th percentiles: 7,10)  17 NT controls (median age 8 yrs; 25th and 75th percentiles: 7,9.5) | Microbiota Analysis:  shotgun metagenomic sequencing of stool samples  ADHD Diagnosis:  -K-SADS-PL  -CPRS  -no information on use of medication by ADHD patients  GI Symptoms:  Not assessed | 1. No difference in alpha diversity of gut microbiota between ADHD groups and NT controls 2. At genus level, compared with the NT control group, *Faecalibacterium* and *Veillonellaceae* were lower in children with ADHD (p<0.05) and *Odoribacter* and *Enterococcus* increased in children with ADHD 3. At the species level, *Faecalibacterium prausnitzii, Lachnospiraceae bacterium*, and *Ruminococcus gnavus* were reduced in ADHD group and *Bacteroides, Odoribacter splanchnicus, Paraprevotella xylaniphila*, and *Veillonella parvula* increased (p<0.05) 4. KEGG analysis of microbiota metabolism identified significant differences in serotonin- and dopamine-related genes between ADHD and NT control groups (p<0.05) 5. association was reported between bacterial changes and constipation symptoms | Strengths:  - NT patients were excluded if on unique diets (i.e., vegetarian), or experiencing other depression/anxiety symptoms  - Age matched NT controls  Limitations:  - Single collection of stool samples  - Small sample size  - Did not perform fecal transplantation to confirm if gut microbiota composition affects ADHD symptoms |
| Richarte et al. 2020  Spain | Assess the intestinal microbiota composition in medication-naïve adults with ADHD | Study Type:  Case-control study of patients with ADHD (medication-naïve) and NT controls  Population:  ADHD patients were referred to ADHD program from primary care centres and adult community mental health services in Spain (Hospital Universitari Vall d’Hebron of Barcelon, Spain)  Sample Size:  100 medication-naïve ADHD patients (mean age 33±11 yrs)  100 NT controls (mean age 30 ±8 yrs) | Microbiota Analysis:  16S rRNA sequencing of stool samples  ADHD Diagnosis:  -DIVA 2.0  -ADHD-RS  -CGI  -WURS  -SDS  -SCID-I and II  -all ADHD patients were medication-naïve  GI Symptoms:  Not assessed | 1. No differences in intestinal microbial α-diversity or β-diversity measures were found between ADHD patients and NT controls 2. At family level, ADHD patients had lower abundance of *Gracilibacteraceae* and higher abundance of *Selenomonadaceae* and *Veillonellaceae* 3. At genus level, ADHD patients had higher abundance of *Dialister, Megamonas* and lower abundance of *Anaerotaenia* and *Gracilibacter* 4. No correlation between scores of ADHD rating scale or factors such as age, BMI, and others with bacterial genera | Strengths*:*  - Patients were excluded based on any other factors that could explain ADHD symptoms i.e., antibiotics or probiotics were not used in the 3 months before sample collection  - Controls were sex and ethnicity matched, and clinical sample isolated to ADHD medication-naïve adult patients  Limitations*:*  - Lifestyle factors such as smoking or dietary habits, stress were not considered |
| Aarts et al. 2017  The Netherlands | Investigate the relationship between gut microbiome and neural reward anticipation in ADHD | Study Type:  Prospective cohort study, of children with ADHD and NT controls.  Population:  ADHD cases derived from the follow-up of the NeuroIMAGE II study.^9^ The sample of NT individuals was compiled of two sub-samples: (i) NT unrelated (n =17) and NT siblings of ADHD probands (n=21) of the ADHD cohort (NeuroIMAGE II)  (ii) self-reported NT volunteers (n= 39) of the Brain Imaging Genetics (BIG) study ^10^.  Sample Size:  *Microbiota analysis*  19 ADHD patients (mean age 19.5±2.5 yrs)  77 NT controls (mean age 27.1±14.3 yrs)  *Imaging analysis*  24 ADHD patients (mean age 20.3±3.7 yrs)  63 NT controls (mean age 21.3±3.4 yrs)  *Microbiota and imaging analyses*  6 ADHD patients (mean age 18.6±2.5 yrs)  22 NT controls (mean age 21.1±3.3 yrs) | Microbiota Analysis:  16S rRNA sequencing of stool samples  ADHD Diagnosis:  -K-SADS  -no information on use of medication by ADHD patients  GI Symptoms:  Not assessed | 1. Actinobacteria (22.14% ADHD vs. 14.08% controls; p=0.002), and Firmicutes (70.29% ADHD vs. 79.80% controls; p=0.001) species differed significantly between ADHD and NT control groups, specifically within the genus *Bifidobacterium* (phylum: Actinobacteria) (20.47% ADHD vs. 12.66% controls; p=0.002). 2. *Bifidobacterium* abundance was still higher in ADHD patients after controlling for age using 15 ADHD and NT age-matched control pairs (18.90% ADHD vs. 13.77% controls; p=0.034). 3. Order Clostridiales (phylum: Firmicutes) was also significantly decreased in ADHD patients (69.02% ADHD vs. 77.37% controls; p=0.003). 4. Differences in relative abundance of genus *Bifidobacterium* strongly contributed to differences in the predicted phenylalanine production through the enzyme CDT 5. fMRI data showed significant association between the reward anticipation responses in the ventral striatum (p=0.048) and predicted CDT abundance 6. No significant association was found between fMRI reward anticipation responses and microbiota composition | Strengths*:*  - A substantial part of the control group consisted of unaffected NT siblings of ADHD cases (21/77), presumably living in the same household with similar diets  - Large sample size  - Functional linkages established between enzymatic pathways and fMRI findings  - Did not observe any differences in BMI between the groups  Limitations*:*  - Functional pathways determined based on inferred metagenome, versus proteomics or metabolomics approach  - 25% of NT participants were siblings of ADHD cases and another sub-sample of the NT group did not undergo clinical screening for ADHD (the BIG sample)  - NT control group was significantly older than the ADHD patients group |
| Szopinska-Tokov et al. 2021  The Netherlands | Identify differences in gut microbiota composition in patients with ADHD and their potential relationship with symptoms of inattention and hyperactivity/impulsivity | Study Type:  Case-control study of individuals with ADHD and NT controls  Population:  Participants were enrolled in the follow-up of the NeuroIMAGE II study (Ethnicity of all patients: Dutch/Caucasian) ^11^  Sample Size:  41 ADHD patients (mean age 20.2±4.1 yrs)  14 subthreshold ADHD patients (mean age 20.3±3.4 yrs)  48 NT controls (mean age 20.4±3.5 yrs) | Microbiota Analysis:  16S rRNA sequencing of stool samples  ADHD Diagnosis:  -K-SADS  -CAARS  -CTRS  -19 ADHD patients reported use of medication  GI Symptoms:  Not assessed | 1. No differences in α- and β-diversity measures between ADHD patients and NT controls 2. *Firmicutes, Bacteroidetes, Actinobacteria, Proteobacteria*, and *Verrucomicrobia* were the most abundant bacterial phyla and no differences were found between ADHD patients and NT controls 3. At the genus level, lower abundance of *Prevotella_9*, *Coprococcus_2* and higher abundance of *Intestinibacter*, were observed in ADHD patients (p<0.05) 4. Reduced abundances of *Lactobacilllus*, *Lachnospiraceae_ND3007_group*, *Ruminococcaceae_g*__, *Ruminococcaceae_UCG.014* (p<0.05) were observed in medicated-ADHD compared to not-medicated ADHD patients 5. ↓Prevotella and Coprococcus = potential reduction in SCFAs production with implications for the MGBA 6. ↑Intestinibacter = mucus-producing bacterium, involved in the pathogenesis of IBD | Strengths:  - Large sample size  - Age matched controls  - ADHD patients taking medication were considered as a separate group in post-hoc analysis  - High-quality clinical assessments of ADHD patients  Limitations:  - Limited sample size, when removal of medicated-ADHD patients; reduced strength of association between bacteria at genus level and ADHD symptoms)  - Self-reported information used for ADHD medication use, no other information available on lifestyle, dietary intake such as probiotics or antibiotics intake |

**Abbreviations:** ADHD = Attention Deficit Hyperactivity Disorder; ADHD-RS = ADHD Rating Scale; ADONIS = ANOSIM = Analysis Of Similarities; BMI = Body Mass Index; CAARS = Conners Adult ADHD Rating Scales; CBCL = Child Behavior Checklist; CDT = Cyclohexadienyl Dehydratase; CGI = Clinical Global Impression; CPRS = Conners' Parent Rating Scale; CTRS = Conners Teacher Rating Scale; DIVA 2.0 = Diagnostic Interview For ADHD In Adults; FBB-HKS = Fremdbeurteilungsbogen FRhyperkinetische StRungen; fMRI = Functional Magnetic Resonance Imaging; GI = Gastrointestinal; KEGG = Kyoto Encyclopedia of Genes and Genomes; K-SADS-E = Epidemiologic Version Of Kiddie-Schedule For Affective Disorders And Schizophrenia; K-SADS-PL = Kiddie-Schedule For Affective Disorders And Schizophrenia Present And Lifetime Version; NT = Neurotypical; rRNA = ribosomal Ribonucleic Acid; SCID-I And-II = The Structured Clinical Interview For DSM-IV Axis I And II Disorders; SDS = Sheehan Disability Inventory; SNAP-IV =Swanson, Nolan, And Pelham Version IV Scale; WISC-IV = Wechsler Intelligence Scale For Children—Fourth Edition; WURS = Wender Utah Rating Scale; Yrs = Years.
