# Supplementary material for: Functional contribution of the intestinal microbiome in autism spectrum disorder, attention deficit hyperactivity disorder, and Rett syndrome: a systematic review of pediatric and adult studies
Source: Front Neurosci. 2024 Mar 7;18:1341656. doi: 10.3389/fnins.2024.1341656 (PMC10954784; doi:10.3389/fnins.2024.1341656)
Supplement: Supplementary file 8 [file Table_8.DOCX]

| **Author, Year,**  **Country** | **Objective** | **Study Type, Population, Sample Size** | **Study Methodology** | **Key Findings** | **Strengths & Limitations** |
| --- | --- | --- | --- | --- | --- |
| Strati et al. 2016  Italy | Investigate the composition and function of the bacterial and fungal gut microbiota in a cohort of RETT subjects | Study type:  Cohort study of individuals with RETT and NT controls  Population:  Female subjects with clinical diagnosis of RETT genotyped for MeCP2 and CDKL5 gene mutations  Sample size:  50 young adult RETT patients (mean age 12±7.3 yrs.)  29 young adult NT controls (mean age 17 ± 9.6) | Microbiota Analysis:  16S rRNA sequencing of stool samples  Mycobiota Analysis:  Fungal ITS1 rDNA region sequencing in stool samples  Rett Diagnosis:  -genotyping for MECP2 and CDKL5 gene mutations.  -CSS with13 criteria  GI Symptoms:  Rome III criteria (constipation)  Others  -ESR, CRP and serum  IgA levels  -Fecal calprotectin | 1. RETT subjects have differences in bacterial and fungal microbiota relative abundances compared to NT controls 2. Reduced microbial richness was found in RETT subjects, and taxa showed higher abundance of Bifidobacterium, several Clostridia (including *Anaerostipes, Clostridium XIVa, Clostridium XIVb*), *Erysipelotrichaceae*, *Actinomyces, Lactobacillus*, *Enterococcus, Eggerthella, Escherichia/Shigella* and the fungal genus *Candida* 3. Alterations of the gut microbiota do not depend on the constipation status of RETT subjects 4. Dysbiotic microbiota resulted in altered production of SCFAs 5. Changes in fungal genera in RETT   (*Significant*)  ↑Candida  (*No significant*)  ↓*Penicillium, Malassezia, Mucor, Eremothecium, Debaryomyces, Pichia, Cyberlindnera*  ↑*Aspergillus*  *Trichosporon* | Strengths:   - All subjects were under a Mediterranean-based diet and no antibiotics, probiotics or prebiotics were taken in the 3 months prior to sample collection   Limitations:   - Small sample size |
| Thapa et al. 2021  USA | Characterize the composition of gut microbiome and metabolome in different RETT phenotypes. | Study type:  Cross-sectional observational study of individuals with RETT and NT controls  Population:  Female subjects with clinical diagnosis of RETT were enrolled in the study. All participants were Caucasian  Sample size:  44 young adult RETT patients (mean age 12.4 yrs.; age range 5.1-36.1 yrs.)  21 young adult NT controls (mean age 10.3 yrs; age range 4.9-27.8 yrs.) | Microbiota Analysis:  16S rRNA sequencing of stool samples  Rett Diagnosis:  CSS:  mild (CSS ≤ 19)  moderate (CSS 20–30)  severe (CSS ≥ 31)  GI Symptoms:  GHQ  Others:  Concentration of microbial metabolites in stool samples with LC-MS  Amino acid profiles in plasma samples with Biochrom Amino Acid Analyzer 30.  Formula:  ⦁ whole cow milk protein source  ⦁ a protein hydrolysate source  ⦁ a soy protein source  an amino acid source alternative plant, nut, and/or meat-based source | 1. Only RETT subjects display GI symptoms 2. ↓Height, weight, and BMI z-scores in the RETT cohort compared with NT 3. lower glutamate and glutamine plasma concentration in RETT-abdominal distention compared to RETT without abdominal distention 4. No differences in microbiome composition were detected between subjects with RETT and NT controls 5. Microbiota composition differed within the RETT cohort based on pubertal status, clinical disease severity scores and type of diet 6. Reduced microbial diversity correlated with increased RETT severity 7. Higher bacterial richness and α-diversity was found in those subjects who consumed table foods (vegetable, and fiber-rich diets) versus liquid-based formula 8. Increased abundance of *Prevotella* was found in subjects consuming table-foods, as compared to increased abundance of *Bifidobacterium* in subjects who received primarily liquid formula 9. GABA, tyrosine, and glutamate were lower in RETT cohort | Strengths:   - Metabolomics approaches along with microbiota sequencing   Limitations:   - Small sample size and many confounders: age, clinical severity of RETT, diet, etc., - Study design needs to be modified to assess differences in abundance of bacterial taxa against confounders - Additional sites of microbe sampling needed apart from single stool sample |
| Borghi et al. 2017    Italy | Assess gut and fecal microbial metabolites to understand relationship between RETT phenotypes and the microbiome | Study type:  Case-control study of individuals with RETT and NT controls  Population:  RETT patients with varying severity followed by Child Neuropsychiatry Department of Santi Paolo Carlo Hospital, Italy.  All subjects and NT controls were Caucasian, living in northern Italy  Sample size:  8 adult RETT patients (mean age 23 + 8.7 yrs.)  10 adult NT controls (mean age 23 + 8.7 yrs.) | Microbiota Analysis:  16S rRNA sequencing of stool samples  Rett Diagnosis:  -RETT diagnostic criteria defined in 2010^5^  -modified SGS  GI Symptoms:  Constipation was assessed but the methodology was not reported  Others:  SCFA concentrations in stool samples with gas liquid chromatography | 1. RETT microbiota had lower α-diversity, increased levels of *Bacteroidaceae*, *Clostridium spp*., and *Sutterella* spp., and reduced abundance of *Ruminococcacae** 2. *Bacteroidaceae, Enterobacteriacae, and Erysipelotrichaceae* increased with increasing severity of RETT; *Lachnospiraceae* and *Ruminococcaceae* decreased with RETT severity 3. Stool SCFA concentrations and acetate were similar between RETT patients and NT controls 4. RETT group showed higher butyrate and propionate levels and BCFAs 5. Higher protein intake was found in the diet of RETT patients 6. PICRUSt-KEGG analysis showed decreased enzymes for carbohydrate and lipid metabolism and increased amino acids pathway and butanoate and propanoate metabolism | Strengths:   - Participants did not take antibiotics or probiotics in 3 months prior to sample collection   Limitations:   - Insufficient sample size to reach statistical analysis for certain analyses (i.e., α-diversity analysis when grouping by disease severity) - Inverse correlation found between relative abundance of Bacteroidetes and BMI, suggesting cohort-specific BMI variations existed that were not controlled for during study design - RETT patients were on anti-epileptic therapies throughout study duration, which could impact microbiota composition |

**Abbreviations:** BCFAs= branched-chain fatty acids; BMI = Body mass index; CDKL5 = cyclin-dependent kinase-like 5; CRP = C-reactive protein; CSS = Clinical Severity Score; GABA = Gamma-aminobutyric acid; GI = gastrointestinal; GHQ = Gastrointestinal Health Questionnaire; ESR = erythrocyte sedimentation rate; IgA = Immunoglobulin A; KEGG = Kyoto Encyclopedia of Genes and Genomes; LC-MS= Liquid chromatography-mass spectrometry; MECP2 = methyl CpG binding protein 2; PCR = polymerase chain reaction; PICRUST = Phylogenetic Investigation of Communities by Reconstruction of Unobserved States; RETT = Rett syndrome; SCFA = short chain fatty acids; SGS= Severity Global Score; yrs. = years.

**Notes:** **Ruminococcaceae* was renamed to *Oscillospiraceae* in 2019.
