# Supplementary material for: Functional contribution of the intestinal microbiome in autism spectrum disorder, attention deficit hyperactivity disorder, and Rett syndrome: a systematic review of pediatric and adult studies
Source: Front Neurosci. 2024 Mar 7;18:1341656. doi: 10.3389/fnins.2024.1341656 (PMC10954784; doi:10.3389/fnins.2024.1341656)
Supplement: Supplementary file 9 [file Table_9.DOCX]

| **Author,**  **Year,**  **Country** | **Objectives** | **Study Type, Population, Sample Size** | **Study Methodology** | **Key Findings** | **Strengths & Limitations** |
| --- | --- | --- | --- | --- | --- |
| Kang et al. 2017  USA | Assess the effects of FMT from healthy donors to ASD children on intestinal microbiota composition and disease-related neurobehavioral outcome | Study type:  Open-label clinical trial of microbiota transfer therapy, after 2-week ABX pre-treatment  Population:  Participants were recruited primarily from the greater Phoenix, Arizona area; three were from outside that area. Healthy control families were recruited from friends of the ASD families and professionals who work with ASD families  Sample size:  18 ASD pediatric patients (mean age 10.8 ± 1.6 yrs.), with moderate to severe GI symptoms  20 NT paediatric control (mean age 11.4 ± 2.5  yrs.), with no GI symptoms | Treatment description:   - 18 weeks total study period (10 weeks FMT, 8 week follow-up). FMT oral vs rectal was compared. Random assignment, unblinded, participants were able to switch groups if they had strong preference for one, or otherwise intolerant to a treatment arm   Exclusion criteria:   - Antibiotic use in prior 6 months or probiotic use in prior 3 months - Dependence on tube feeding - Severe GI problems that require immediate treatment (life-threatening) - Recent/scheduled surgeries - Diagnosed as severely malnourished or underweight - Single-gene disorder - Major brain malformations - Inflammatory bowel disease - Celiac disease - Eosinophilic esophagitis   Other notes:   - ASD diagnosed with ADI-R - GI symptoms assessed with GSRS - Microbiota composition evaluated by 16S rRNA sequencing | 1. 80% reduction in GI complaints (constipation, diarrhea, indigestion, abdominal pain) 2. Improvements continued at 8-week follow-up 3. Improvements in behavioural symptoms 4. Partial engraftment of donor microbiome and virome, increased overall diversity 5. *Bifidobacterium, Prevotella*, and *Desulfovibrio* increased and persisted at 8-week follow-up period 6. Children with ASD had more C-section deliveries, non-standard formula feedings with shorter breastfeeding duration, food allergies, eczema, lower fibre intake (infants and mothers) 7. Dietary carbohydrat, fat, protein, calorie intake comparable across ASD and controls 8. Children with ASD had greater antibiotic use in first 4 years of life than controls | Strengths:   - Assessed oral vs rectal enemas - Clear description of NT children, including no first-degree relative with neurodevelopmental/neurological disorders - Clear inclusion/exclusion criteria - Good attention to excluding confounders (neuro and GI disorders) - Use of ABX pre-treatment, PPI, bowel prep, and 1-day fasting prior to increase donor engraftment - Age, gender, BMI matched controls   Limitations:   - Unblinded study design raises concerns for bias - Small sample size - Short follow-up period - Ability for ASD participants to swap groups based on “strong preference or intolerance” is concerning. These were not defined - Microbiota for FMT given in food/drink; concerns with reaching stomach/surviving stomach acidity without capsule formulation - No placebo - Results may be confounded by use of PPI, vancomycin, bowel prep, versus FMT alone |
| Shaaban et al. 2018  Egypt | Assess the efficacy and tolerability of probiotic supplementation in children with ASD in the context of restoring gut microbial dysbiosis, GI disturbances, and behavioral abnormalities | Study type:  Prospective cohort, and prospective case-control design  Population:  Children with ASD recruited from child psychiatry unit of Ain Shams University Hospital, and developmental pediatric clinic of the National Research Center, Cairo, Egypt  Sample:  30 ASD pediatric patients (mean age 7.06±1.36 yrs.)  30 NT pediatric controls (family relatives) mean age not reported | Treatment description:   - All children with ASD received probiotics in powder formulation, consisting of dried carrot with three probiotic strains (*Lactobacillus acidophilus, Lactobacillus rhamnosus* and *Bifidobacteria longum*). Daily dose was 5 g of the powder (each gram contains 10 × 10^6^ colony forming units of the three probiotic strains) dissolved in water, given once daily for 3 months   Exclusion criteria:   - Other neurodevelopmental disorders or psychiatric diseases - Chronic medical condition, including; anemia, brain malformations, metabolic diseases, epilepsy, organic - GI disorders (i.e. gastroesophageal reflux, food allergies, IBS, and celiac disease) - Concomitant use of anti-fungals, antibiotics, special diets - Use of psychiatric medications within preceding 3 months   Other notes:   - ASD severity assessed with the ATEC score prior to, and following probiotics supplementation - GI symptoms assessed by using a modified version of the 6-GSI - Microbiota composition assessed through real-time PCR | 1. After probiotic intake for 3 months, overweight children with ASD had significant decrease in body weight (P < 0.014) and BMI (P < 0.01) 2. At baseline, *Bifidobacteria* was significantly lower in children with ASD versus controls (P = 0.0001). After probiotic supplementation, significant increases were found in *Bifidobacteria* and *Lactobacillus* in stool PCR of children with ASD (P < 0.0001) 3. Total ATEC scores (ASD symptoms) were significantly decreased after probiotic supplementation among children with ASD (P = 0.0001): speech/language/communication (P < 0.017), sociability (P < 0.001), sensory/cognitive awareness (P < 0.026), and health/physical/behaviour domains (P < 0.0001) 4. Probiotic supplementation significantly improved total 6-GSI score (GI symptoms) (P < 0.0001). This included improvements in constipation (P < 0.01), stool consistency (P < 0.023), flatulence (P < 0.037), abdominal pain (P < 0.002) 5. No adverse effects reported. Side effects reported were diarrhea (one patient), bloating (two patients), abdominal cramps (two patients) and skin rash (one patient) (all mild, transient) | Strengths:   - Compliance to treatment done well - 3-month duration of treatment - Appropriate and rigorous testing of cases - Appropriate timing of assessments (baseline and 3 months)   Limitations:   - No power calculations performed - Controls were healthy age-matched relatives Relatives may not be ideal controls, particularly cohabitants, as microbial signatures tend to be similar within cohabitants - Baseline characteristics, anthropometric measures, and psychiatric assessments not performed on healthy controls. Therefore, unable to assess appropriateness of controls |
| Tomova et al. 2015  Slovakia | Assess intestinal microbiota of children with ASD before and after probiotic supplementation | Study type:  Prospective, case-control design  Population:  Children with ASD recruited from local pediatric Autism Centre in Bratislava, Slovakia  Sample size:  10 paediatric ASD patients (age range 2-9 yrs.)  9 paediatric NT controls siblings (age range 5-17 yrs.)  10 pediatric NT controls unrelated (age range 2-11 yrs.) | Treatment Description:   - Dietary supplementation comprised of one capsule of “Children Dophilus” containing 3 strains of Lactobacillus (60%), 2 strains of *Bifidobacteria* (25%) and one strain of *Streptococcus* (15%). One capsule was taken three times a day for a total of 4 months - RT-PCR performed on microbial genomic DNA - TNFα measured in stool supernatants - Plasma oxytocin, testosterone and DHEA-S measured in all subjects   Exclusion Criteria:   - No info on exclusion/inclusion criteria   Other Notes:   - Children with ASD underwent psychological evaluation using CARS, ADI, and a semi-structured interview with parents regarding child's behaviour - GI symptoms assessed using GSRS | 1. There was a strong positive correlation between the intensity of GI symptoms and the severity of ASD, scored on ADI scale (R = 0.78, P = 0.01) 2. The Bacteroidetes/Firmicutes ratio was significantly lower in children with ASD compared to controls (P < 0.05) 3. Strong correlation of *Desulfovibrio* with the ADI in restricted/repetitive behavior subscale score (R = 0.83, P < 0.05) 4. Probiotic supplementation increased the Bacteroidetes/Firmicutes ratio in children with ASD to the level of the healthy individuals 5. Probiotic supplementation decreased *Bifidobacterium* significantly in children with ASD and reached the level of the gastrointestinal content of the healthy subjects 6. Probiotic supplementation increased the relative amount of *Lactobacillus* by two-fold 7. Probiotic supplementation significantly decreased relative amount of *Desulfovibrio* (P < 0.05) 8. Healthy siblings had significantly higher Firmicutes and a trend to a decreased amount of Bacteroidetes, with a significantly decreased ratio of Bacteroidetes/Firmicutes compared to controls (P < 0.05) 9. Mean fecal TNFa was increased in children with ASD and their healthy siblings compared to healthy unrelated controls but was not significant 10. Strong correlation between TNFα levels and GI symptoms (R = 0.78, P < 0.05) 11. Probiotic supplementation significantly decreased the TNFα levels in the stool of children with autism (P < 0.05) 12. Plasma oxytocin was significantly lower in ASD and healthy siblings compared to neurotypical unrelated controls (P < 0.05). There was a significant positive correlation of plasma oxytocin levels and autism severity (ADI) (R = 0.71, P < 0.05) 13. Plasma level of DHEA-S was significantly lower in children with ASD, compared to healthy children and compared to autistic siblings (P < 0.05) 14. Testosterone levels did not differ between groups, but showed a significant positive correlation with autism severity (ADI) (R = 0.74, p < 0.05) | Strengths:   - Children with ASD were appropriately assessed (CARS, ADI, parent interviews) - GI symptoms were appropriately recording using a standardized questionnaire - Confounders were appropriately described   Limitations:   - Considerably more boys than girls across all three groups. Some expected discrepancies in age groups between children with ASD and healthy sibling controls. However, these may impact findings - P values appropriate for every test (P < 0.05) - Very small sample size in each group |
| Partty et al. 2015  Finland | Determine if probiotic supplementation in early life reduces the risk of development of ADHD or ASD in adolescence | Study type:  RCT assessing the predisposition to ADHD and AS in children treated with probiotics in early life.  Population:  Participants in an ongoing, randomized, double-blind, placebo-controlled prospective follow-up study involving perinatal Lactobacillus rhamnosus GG (ATCC 53103) intervention^5^  Sample size:  40 subjects treated with probiotic (age range: birth-13 yrs.)  35 subjects treated with placebo (age range: birth-13 yrs.) | Treatment description:   - Mothers received 1x10^10^ CFU of *Lactobacillus rhamnosus GG* or placebo (microcrystalline cellulose) daily for 4wk before expected delivery - Infants were randomized to receive either *Lactobacillus rhamnosus GG* or placebo for 6 months, and followed up for 13 years - No details on exclusion/inclusion criteria - Microbiota composition was assessed at following timepoints: 3 weeks, 3, 6, 12, 18, 24 months and 13 years through direct FISH/qPCR and indirectly through blood secretor type - Diagnoses of either ADHD or AS was made by neurologists/ psychiatrists | 1. At age 13 years, ADHD was diagnosed in 3 children in the placebo group, AS in 1 child, and both ADHD and AS in 2 children (4.0%, 1.3%, and 2.7%, respectively) (all male) 2. No children in probiotic group were diagnosed with ASD (P = 0.008) 3. *Bifidobacterium* species lower at 6-months in children with AS and ADHD compared to healthy controls (P = 0.03) 4. At age 13 years, there was no difference in gut microbiota composition (assessed with both FISH and qPCR techniques) for children with/without neuropsychiatric disorders | Strengths:   - 13 years follow up - Gut microbiota assessed through qPCR, FISH, and blood group secretor type   Limitations:   - Study is underpowered, originally designed for assessing the effect of the probiotic on eczema - Dropouts throughout 13-year period, affecting final results |
| Stevens et al. 2020  New Zealand | Characterize changes in human gut microbiota during a 10-week randomised controlled trial of micronutrient supplementation in children with attention deficit hyperactivity disorder | Study Type:  Pilot randomized, double-blind, placebo-controlled trial assessing the effect of micronutrient supplementation on ADHD symptoms  Population:  Subset of children who participated in a 10-week double-blind trial comparing a broad-spectrum micronutrient treatment with a placebo to investigate therapeutic effects on ADHD symptoms. 13 New Zealand Europeans and four New Zealand Māori participants were included.  Sample Size:  10 children with ADHD received study treatment  8 children with ADHD received placebo treatment (age range: 7-12 yrs.). | Treatment description:   - Administration of capsules (formulation containing a blend of vitamins, minerals, amino acids and antioxidants) or placebo to ADHD subject. - One capsule, three times each day, increasing the dose by three capsules every two days up to a target dose of 12 capsules per day: four taken at three different intervals. - No details on exclusion/inclusion criteria) - For each participant, a pre-RCT, and a post-RCT fecal sample was collected and sequenced for microbiota analysis (16S rRNA gene sequencing). - Participants were monitored for ADHD symptoms by a clinical psychologist or psychology graduate student, under the supervision of a psychologist (with CGAS and ADHD-RS IV). | 1. ↓Firmicutes/Bacteroidetes ratio because of ↑ in Bacteroidetes abundance and frequency in both groups between pre-RCT and post-RCT 2. Patients receiving micronutrient treatment had improved symptoms of attention, emotional regulation, and aggression relative to placebo 3. *Bifidobacterium* genera decreased by 25% in the micronutrient treatment group (p = 0.056) 4. Increase in bacteria from the order Coriobacteriales (p=0.057) in the micronutrient treatment group | Strengths:   - Intervention trial - Dietary supplement - Paired statistical analysis   Limitations:   - Small sample size - Further validation required |

**Abbreviations:** ABX = antibiotics; ADI-(R) = Autism Diagnostic Interview (Revised); ADHD = attention deficit hyperactivity disorder; ADHD-RS = ADHD rating scale; ASD = Autism Spectrum Disorder; AS = Asperger’s Syndrome; ATEC = Autism Treatment Evaluation Checklist; BMI = Body mass index; CARS = Childhood Autism Rating Scale; CFU = colony forming unit; CGAS = Children’s Global Assessment Scale; DHEA-S = dehydroepiandrosterone sulfate; FISH = Fluorescence In Situ Hybridization; FMT = Fecal Microbiota Transplantation; GI = gastrointestinal; 6-GSI = GI Severity Index; GSRS = Gastrointestinal Symptom Rating Scale; IBS = irritable bowel syndrome; PPI = proton pump inhibitor; qPCR = quantitative polymerase chain reaction; RCT = randomized control trial; TNFα = Tumor necrosis factor α; yrs. = years.
