# Supplementary material for: Functional contribution of the intestinal microbiome in autism spectrum disorder, attention deficit hyperactivity disorder, and Rett syndrome: a systematic review of pediatric and adult studies
Source: Front Neurosci. 2024 Mar 7;18:1341656. doi: 10.3389/fnins.2024.1341656 (PMC10954784; doi:10.3389/fnins.2024.1341656)
Supplement: Supplementary file 10 [file Table_10.DOCX]

| **Reference** | **Disorder** | **Sample** | **Sample Storage** | **DNA Extraction** | **Sequencing Technique/ Target** | **Sequence Corrections, Filtering** | **Reference Database** |
| --- | --- | --- | --- | --- | --- | --- | --- |
| Zhang et al. 2018 | ASD | Stool sample | - Collected at home, shipped to laboratory for freezing - Stored at −80 °C | NEXTflex Rapid DNA-Seq Kit (Bioo Scientific®, USA) | - 16S rRNA sequencing - V3-V4 hypervariable regions HiSeq 2500 sequencing platform (Illumina®, USA) | Human microbe-disease association database (HMDAD), microbe similarity between ASD and other disease | GreenGenes database  QIIME used to calculate alpha, beta diversity  PICRUSt software used for functional analyses |
| Liu et al. 2019 | ASD | Stool sample | - Collected in fecal bacteria DNA storage tubes (Tinygene®, China) - Stored at −80 °C | QIAamp Fast DNA Stool Mini Kit (QIAGEN®, USA) | - 16S rRNA sequencing - V3-V4 hypervariable regions - MiSeq sequencing platform (Illumina®, USA) | Silva (SSU123) reference database used to filter sequences (confidence threshold 70%)  Sequences classified using RDP Classifier algorithm  Chimeric sequences identified and removed using UCHIME  Rarefaction applied to OTUs | OTUs clustered with 97% similarity cutoff using UPARSE (v7.1)  Sequencing data computed using phyloseq R package (v2.15.3) |
| Kang et al. 2013 | ASD | Stool sample | - Shipped overnight to laboratory with cold pack - Stored at −80 °C | QIAamp Fast DNA Stool Mini Kit (QIAGEN®, Japan) | 16S rRNA sequencing  V2-V3 hypervariable regions  GS FLX Titanium sequencing platform (Roche®, USA) | Sequences removed using ChimeraSlayer  Sequences classified using RDP Classifier software at 50% (>200bp), 80%-confidence (>250bp) threshold  Rarefaction applied to OTUs | SSURef database  OTUs clustered with 90, 95, 97% similarity cutoff using UCLUST  QIIME used to calculate phylogenetic diversity  Classifications assigned by Python (SciPy stats library) and R packages |
| Kang et al. 2018 | ASD | Stool sample | NR | PowerSoil DNA Extraction Kit (Mobio®, USA) | 16S rRNA sequencing  V2-V3 hypervariable regions  GS FLX Titanium sequencing platform (Roche®, USA) | Sequences removed using ChimeraSlayer  Sequences classified using RDP Classifier | GreenGenes database  QIIME used to calculate phylogenetic diversity  OTUs clustered with 97% similarity cutoff using UCLUST  PICRUSt software used for functional analyses |
| Rose et al. 2018 | ASD | Stool sample | - Collected with RNAlater (Sigma-Aldrich®, USA) - Kept at −20 °C for <24 hrs - Stored at −80 °C | PowerSoil DNA Extraction Kit (Mobio®, USA) | 16S rRNA sequencing  V3-V4 hypervariable regions  MiSeq sequencing platform (Illumina®, USA) | *de novo* chimera detected conducted in UCHIME (v5.1) | GreenGenes database  QIIME (v1.8.0) used to calculate phylogenetic diversity  OTUs clustered with 97% similarity cutoff using USEARCH  Phyloseq R package used to calculate alpha, beta diversity  PICRUSt software used for functional analyses |
| Finegold et al. 2017 | ASD | Stool sample | Stored at −80 °C | NR (samples plated on culture media) | ABI 3130 Avant Sequencer (Thermo Fisher®, USA) | NR | GenBank |
| Zhai et al. 2019 | ASD | Stool sample | - Transported to laboratory same day at −4 °C - Stored at −80 °C | FastDNA SPIN Kit for Feces (MP Biomedicals®, USA) | 16S rRNA sequencing  V3-V4 hypervariable regions  MiSeq sequencing platform (Illumina®, USA) | Chimeras excluded using B2C2 | Database NR  QIIME (v1.9.1) used to calculate phylogenetic diversity  R vegan package used to calculate beta-diversity  PICRUSt software used for functional analyses |
| Strati et al. 2017 | ASD | Stool sample | Stored at −80 °C | FastDNA SPIN Kit for Feces (MP Biomedicals®, USA) | 16S rRNA sequencing  V3-V5 hypervariable regions  Fungal ITS1 rDNA region  GS FLX Titanium sequencing platform (Roche®, Switzerland) | Sequences classified using RDP Classifier algorithm (v2.7 16S data; v2.8 fungal ITS data)  Template-guided multiple sequence alignment (MSA) using PyNAST (v0.1 16S data); T-Coffee (fungal data)  Rarefaction applied to OTUs | GreenGenes (16S data) and GenBank (fungal data) databases  OTUs clustered with 97% pairwise identity  Phyloseq R package used to calculate alpha, beta diversity |
| Hughes et al. 2018 | ASD | NR | NR | NR | NR | NR | NR |
| Kantarcioglu et al. 2016 | ASD | Stool sample | - Kept at 4 °C for <24 hrs - Transported to laboratory within 8 hours - Stored at −80 °C | NR | NR | NR | Identification by classical morphological and biochemical tests |
| Son et al. 2015 | ASD | Stool sample | - Collected with with RNAlater (Sigma-Aldrich®, USA) - Transported overnight to laboratory with cold pack - Stored at −80 °C | ZR Fecal DNA MiniPrep (Zymo®, USA) | 16S rRNA sequencing  V1-V3 hypervariable regions  MiSeq sequencing platform (Illumina®, USA) | Chimeras identified in UCHIME  Sequences aligned and classified with SINA (v1.2.11) | Silva (115NR99) reference database  R vegan package (ADONIS function) used to calculate alpha, beta diversity |
| Gondalia et al. 2012 | ASD | Stool sample | - Shipped overnight to laboratory with frozen gel ice pack - Stored at −20 °C | QIAamp Fast DNA Stool Mini Kit (QIAGEN®, USA) | GS FLX Titanium sequencing platform (Roche®, USA) | Chimeras excluded using B2C2 | Database NR  QIIME, R vegan, R labdsv packages used to calculate phylogenetic diversity |
| De Angelis et al. 2013 | ASD | Stool sample  Overnight morning fasting sample  3-day pooled collection | - Collected under anaerobic conditions with RNAlater (Sigma-Aldrich®, USA), or Amies Transport Medium (Oxoid®, UK) - Stored at −80 °C | FastDNA Pro Soil Direct Kit (MP Biomedicals®, USA) | 16S rRNA sequencing  V1-V3 hypervariable regions  bTEFAP was performed by Research and Testing Laboratories (Lubbock, TX), according to standard laboratory procedures using a 454 FLX Sequencer (454 Life Sciences, Branford, CT, USA. | Chimeras excluded using B2C2  Sequences <250bp removed | GenBank database  QIIME used to calculate phylogenetic diversity  OTUs clustered with 77, 80, 85, 90, 95, 97% similarity cutoff using USEARCH  FASTA sequences evaluated using BLAST |
| Pulikkan et al. 2018 | ASD | Stool sample | Stored at −80 °C | QIAamp Fast DNA Stool Mini Kit (QIAGEN®, Germany) | 16S rRNA sequencing  V3 hypervariable regions  NextSeq500 (Illumina®, USA) | NR | GreenGenes database (v13.5)  OTUs clustered with 97% similarity cutoff  QIIME (v1.5.0) and R used to calculate phylogenetic diversity |
| Ahmed et al. 2020 | ASD | Stool sample | Stored at −20 °C | ISOLATE Fecal DNA Kit (Bioline, UK) | 16S rRNA sequencing  Rotor-Gene Q (QIAGEN, Germany) using a SensiFAST™ SYBR® No-ROX PCR kit (Bioline Co., UK). | Data analysis was carried out using the Statistical Package for Social Sciences version 20 (SPPS PASW Statistics, Chicago) | Alpha diversity calculated with Shannon diversity index |
| Yap et al. 2021 | ASD | Stool sample | Stored at −80 °C | QIAamp 96 PowerFecal QIAcube HT Kit (QIAGEN®, Japan) | Metagenomics sequencing  NovaSeq6000 platform (Illumina®, USA)  MGPP tool | Sequence trimming using Illumina BaseSpace Bcl2fastq2 (v2.20) and Trimmomatic (v0.39)  Alignments were further filtered using SAMtools v1.7 | MGENES v2.0.0 reference database  PERMANOVA used to calculate beta diversity  Shannon index used to calculate alpha diversity |
| Kushak et al. 2017 | ASD | Duodenal biopsies | - Snap frozen - Stored at −80 °C | Mo Bio  DNA Isolation kits (Mo Bio Laboratories, Inc, Carlsbad, CA), | 16S rRNA gene  Sequencing  Roche 454 FLX instrument with Titanium reagents, and Titanium procedures performed at the Research and Testing Laboratory (Lubbock, TX) | Chimeras checked using UCHIME | GreenGenes database (v12.10)  OTUs clustered with 97% similarity cutoff using USEARCH  R vegan (ADONIS function), labdsv, DESeq packages used to conduct statistical analyses and calculate phylogenetic diversity |
| Luna et al. 2017 | ASD | Rectum biopsies | - Snap frozen - Stored at −80 °C | MO BIO PowerSoil extraction  kit protocol (MO BIO Laboratories). | 16S rRNA gene  Sequencing  V1-V3 and V4 hypervariable regions  Illumina MiSeq platform (Illumina, San Diego, CA). | Sequence data were processed through the LotuS pipeline  Quality filtering was performed using a modified version of the UPARSE algorithm. | Taxonomic assignment was performed with RDP as the classifier and HitDB40 and SILVA41 as the selected databases. |
| Jiang et al. 2018 | ADHD | Stool sample | - Kept at −20 °C following collection - Transported to laboratory by patient - Stored at −80 °C | QIAamp Fast DNA Stool Mini Kit (QIAGEN®, USA) | 16S rRNA sequencing  V3-V4 hypervariable regions  MiSeq sequencing platform (Illumina®, USA) | NR | QIIME (v1.7) used to calculate phylogenetic diversity  OTUs clustered with 97% pairwise identity |
| Prehn-Kristensen et al. 2018 | ADHD | Stool sample | - Stored at 4 °C - DNA stored at −80 °C following extraction | FastDNA SPIN Kit for Feces (MP Biomedicals®, USA) | 16S rRNA sequencing  V1-V2 hypervariable regions  MiSeq sequencing platform (Illumina®, USA) | NR | Database NR  R vegan (ADONIS function) package (v2.4.1) used to calculate phylogenetic diversity  OTUs clustered with 97% pairwise identity |
| Wang et al. 2020 | ADHD | Stool sample | - Kept at 4 °C - Transported to laboratory within 24 hrs - Stored at −80 °C | QIAamp DNA Stool Mini Kit (QIAGEN®, Japan) | 16S rRNA sequencing  V3-V4 hypervariable regions  MiSeq sequencing platform (Illumina®, USA) | SILVA reference (release 132) used to filter, trim sequences  Chimeric sequences identified and removed using UCHIME (v4.2) | QIIME and R used to calculate phylogenetic diversity |
| Wan et al. 2020 | ADHD | Stool sample | Stored at −80 °C | HiPure Stool DNA kits (Angen Biotech Co., Ltd., Guangzhou, China) | Metagenomics  sequencing  Illumina NovaSeq platform (Illumina, San Diego, CA, United States) reading length of 150 bp (PE150) | NR | Gene Catalog databases  Kyoto Encyclopedia of Genes and Genomes (KEGG) |
| Richarte et al. 2020 | ADHD | Stool sample | Stored at −80 °C | QIAamp® PowerFecal® DNA extraction kit (QIAgen, Hilden, Germany) | 16S rRNA sequencing  V3−V4 hypervariable region  PicoGreen™ dsDNA Assay Kit | Reads without both primer sequences or with less than 200 bp were discarded with Cutadapt v.1.8.1  Chimeric sequences were removed using the UCHIME software  OTUs with nonzero values in less than 10% of the samples were removed | NCBI 16S rRNA reference database |
| Aarts et al. 2017 | ADHD | Stool sample | - Kept at 4 °C following collection - Transported to laboratory within 24 hrs - Stored at −80 °C | DNeasy Blood and Tissue Kit (QIAGEN®, The Netherlands) | 16S rRNA sequencing  V3-V4 hypervariable regions  GS FLX Titanium sequencing platform (Roche®, USA) | NR | QIIME (v1.2) used to calculate phylogenetic diversity  Functional metagenomics performed using PICRUSt |
| Szopinska-Tokov et al. 2021 | ADHD | Stool sample | Stored at −80 °C | Maxwell® 16 Instrument (Promega, Leiden, The Netherlands) | 16S rRNA sequencing  V1-V2 hypervariable regions  llumina HiSeq PE300 sequencing platform (GATC Biotech AG, Konstanz, Germany) | Two filtering steps on the output file (BIOM-file) of NG-Tax  Regression analyses were corrected for multiple testing using the false discovery rate and indicated as q-values. | SILVA reference database (v128)  OTUs clustered based on a sequence similarity ≥98.5% |
| Strati et al. 2016 | Rett syndrome | Stool sample | Stored at −80 °C | FastDNA SPIN Kit for Feces (MP Biomedicals®, USA) | 16S rRNA sequencing  V3-V5 hypervariable regions  Fungal ITS1 rDNA region  GS FLX Titanium sequencing platform (Roche®, Switzerland) |  | GreenGenes database (16S data)  GenBank database (fungal data)  OTUs clustered with 97% pairwise identity  Phyloseq R package used to calculate alpha, beta diversity |
| Thapa et al. 2021 | Rett syndrome | Stool sample | Stored at −80 °C | OMNIgene GUT kit (DNA Genotek Inc., Ontario, Canada) | 16S rRNA sequencing  V4 hypervariable regions  MiSeq sequencing platform (Illumina®, USA) | Samples with <1000 read counts were excluded | PERMANOVA used to calculate beta diversity  Phyloseq R package used to calculate beta diversity |
| Borghi et al. 2017 | Rett syndrome | Stool sample | NA | Spin stool DNA kit (Stratec Molecular, Berlin, Germany) | 16S rRNA sequencing  MiSeq sequencing platform (Illumina®, USA)  V3−V4 hypervariable region  Paired end 2x250 bp sequencing | Singleton OTUs were discarded as possible artifacts or unlikely bona fide bacterial sequences  Fragments were filtered using the “split_libraries_fastq.py” utility of the QIIME suite | Greengene bacterial 16S rRNA database (v13.8) by RDP classifier at 50% confidence  Sequences were grouped into OTUs by using UCLUST with 97% similarity |
| Kang et al. 2017 | Intervention for ASD (FMT) | Stool sample  Fecal swab | - Kept at −20 °C following collection - Shipped overnight to laboratory with cold packs - Stored at −80 °C | Bacterial DNA: PowerSoil DNA Extraction Kit (Mobio®, USA)  Viral DNA: DNeasy Blood and Tissue Kit (QIAGEN®, The Netherlands) | 16S rRNA sequencing  V4 hypervariable regions  MiSeq sequencing platform (Illumina®, USA) | Sequences classified using RDP Classifier algorithm  VirSorter used to generate non-redundant dataset of viral contigs | GreenGenes database (v13.5)  QIIME (v1.9.1) used to calculate phylogenetic diversity (bacterial populations)  OTUs clustered with 97% similarity cutoff  R vegan (v.3.2.3) used to calculate phylogenetic diversity (viral populations)  Prodigal, Viral Protein RefSeq used to predict viral genes |
| Shaaban et al. 2017 | Intervention for ASD (probiotic treatment) | Stool sample | NR | NR | NR | NR | NR |
| Tomova et al. 2014 | Intervention for ASD (probiotic treatment) | Stool sample | Delivered to laboratory within 4 hours  Stored at −80 °C | QIAamp Fast DNA Stool Mini Kit (QIAGEN®, Japan) | Specific PCR primers used (EuroFins MWG Operon®, Germany) | NR | Previously reported by authors(89) |
| Pärtty et al. 2015 | Intervention for AS and ADHD (probiotic treatment) | Stool sample | - Kept at 4 °C following collection - Transported to laboratory within 24 hrs - Stool stored at −80 °C until DNA extraction - DNA stored at −20 °C following extraction | KingFisher DNA Extraction System (Thermo Fisher®, USA)  InviMag Stool DNA Kit (Invitek Molecular®, Germany) | Targeted qPCR  ABI 3130 Avant Sequencer (Thermo Fisher®, USA) | NR | NR |
| Stevensen et al. 2019 | Intervention for ADHD (micronutrient) | Stool sample | - Kept at −4 °C - Transported to laboratory within 14 days - Stored at −80 °C | NucleoSpin SPIN Stool Isolation Kit (Macherey-Nagel®, Germany) | 16S rRNA sequencing  V3-V4 hypervariable regions  MiSeq sequencing platform (Illumina®, USA) | OTUs observed <100x removed | GreenGenes database (v13.8)  QIIME2 (v2017.12) used to calculate phylogenetic diversity  Functional metagenomics performed using Tax4Fun R package, and PICRUSt |

**Abbreviations:** B2C2 = Black box chimera check; DNA = Deoxyribonucleic acid; ITS = Internal transcribed spacer; MGPP = Microba Gene and Pathway Profiler; MGENES = Microba Genes; NR = Not reported; OTU = Operational taxonomic unit; PICRUSt = Phylogenetic Investigation of Communities by Reconstruction of Unobserved States; QIIME = Quantitative Insights Into Microbial Ecology; RDP = Ribosomal Database Project; rRNA = Ribosomal ribonucleic acid; PERMANOVA = Permutational multivariate analysis of variance; MGDB = Microbial Genome Database.
